# Supplementary material for: Young people’s preferences for the use of emerging technologies for asymptomatic regular chlamydia testing and management: a discrete choice experiment in England
Source: BMJ Open. 2019 Jan 29;9(1):e023663. doi: 10.1136/bmjopen-2018-023663 (PMC6352830; doi:10.1136/bmjopen-2018-023663)
Supplement: Supplementary file 1 [file bmjopen-2018-023663supp001.pdf]

Supplementary File 1 – Selection of Attributes and Levels

Four research stages were used to determine the attributes and associated levels included in the Discrete Choice Experiment.

1. LITERATURE REVIEWS

1.1. Overarching Objectives

- To identify a long list of attributes
- To identify potential levels
- To identify gaps in knowledge
- To inform the development of the focus groups

1.2. Literature Review 1 - Use of Stated Preference Studies for STI testing & treatment services

1.2.1. Objective

To identify and appraise published studies exploring which attributes influence patient and clinician preferences for the testing and treatment of STIs.

1.2.2. Inclusion/ Exclusion Criteria

The inclusion criteria were identified as:

- any stated preference study within the scope of STI testing and treatment services. This included but was not limited to products (e.g. tests, drugs, condoms, microbicides) and services (e.g. screening and screening programmes, and service providers e.g. GPs, CaSH clinics and GUM clinics);
- There was no date limiter, with all published studies included to end of 2014.

Exclusion criteria included studies:

- not related to humans;
- not published in English;
- from outside of the OECD High Income Countries;
- not related to the diagnosis or treatment of STIs (e.g. vaccinations).

1.2.3. Search Strategy

The following databases were searched on 28 April 2014 to identify studies published to the end of 2013, the saved database searches were re-run in April 2015 to search for any studies meeting the inclusion criteria published between 1 January and 31 December 2014 and no further studies were identified:

- Medline
- EMBASE
- CINAHL
- Web of Science
- Econlit

- PsycINFO
- Cochrane Library (incorporating the following databases):
    - Cochrane Database of Systematic Reviews
    - Cochrane Central Register of Controlled Trials
    - Cochrane Methodology Register
    - Database of Abstracts of Reviews of Effects
    - Health Technology Assessment Database
    - NHS Economic Evaluation Database

Searches were structured to meet the search requirements of the respective database and terms expanded where the facility existed to do this.

1.3. Literature Review 2 – Preferences for and acceptability of mainstream sexual health services

1.3.1. Objective

The objective of this literature review was to identify which factors might influence individuals' decisions to access testing and treatment services for STIs.

1.3.2. Inclusion/ Exclusion Criteria

The inclusion criteria were identified as:

- any study which indicates individuals' preferences or acceptability of STI testing and/ or treatment services;
- studies published between Jan 2004-Sept 2014;
- conference abstracts, where the abstract enabled the extraction of information on study focus and key findings.

The date range for the literature review was selected to limit the volume of results identified to the last 10 years. This recognised the fact that the previous literature review only identified one stated preference study which met the inclusion criteria pre-2004, and that none of the studies identified included relevant new technologies e.g. internet or smartphone based services.

The exclusion criteria were identified as any study:

- not published in English
- not related to humans
- not related to preferences for sexual health services
- from outside of the OECD High Income Country List
- not related directly to testing and/ or treatment provision e.g. drug characteristics, health promotion interventions
- not offering a perspective provided by service user/ potential service user e.g. clinician
- focused on non-mainstream service provision e.g. STI testing in A&E, dedicated service provision for specific high risk groups such as men recently released from prison, sex workers, injecting drug users.

1.3.3. Search Strategy

The key search terms included were:

- Sexually transmitted infections, sexually transmitted diseases, sexual health
- Test, treatment, service
- Patient preference, acceptability, choice, uptake, access.

Individual STIs (e.g. Chlamydia) were not included in the search strategy, as it was determined that attributes and levels could potentially be identified from any STI/ sexual health product or service.

A smaller selection of databases was chosen for this scoping review, with the databases selected being those which had generated a high return rate of relevant articles in the first literature review and encompassing journals where research relating to nursing, AHPs and psychology is published.
The three databases searched were:

- Medline
- CINAHL
- PsycINFO.

2. FOCUS GROUPS

2.1. Objectives

Primary Objective:

- To identify which the themes and factors young people consider important when choosing whether to test for sexually transmitted infections

Secondary Objectives:

- To gain insight into:
    - Reasons for importance of factors
    - Rationale for trading between factors and prioritisation
    - How participants articulate views and opinions on these themes/ factors.

2.2. Inclusion/ Exclusion Criteria

Participant inclusion criteria set for the focus groups were:

- Between the ages of 16 and 24
- Ability to speak English
- Able to consent.

Exclusion criteria were:

- People under the age of 16 and over the age of 25
- People requiring any form of interpreting/ translation services to participate
- People unable to give consent.

2.3. Methods

2.3.1. Sampling

Convenience sampling was ultimately chosen for this research due to the challenges with accessing the population and being able to undertake purposive sampling within the time constraints of this phase of the research.

2.3.2. Data Management & Analysis

Thematic analysis was selected as the core approach to analyse the focus group data recognising that this is one of the three ‘main methods’ for analysing focus group data and is the method most closely aligned to that used to design the focus group.

3. EXPERT GROUPS

3.1. Objectives

The objectives of the expert groups were to consider whether:

- The potential attributes met best practice requirements as defined by ISPOR Conjoint Analysis working group (Bridges et al., 2011) as “a subset of all possible attributes can be determined on the basis of three criteria:
    - Relevance to the research question;
    - Relevance to the decision context;
    - Whether attributes are related to one another (Bridges et al., 2011:406);
- Any key attributes omitted that may lead response bias;
- Levels are sufficiently reflective of current and potential future technology developments.

3.2. Methods

Expert groups were run as focus groups to enable discussion around a series of key questions. The key issues raised by each of the expert groups were summarised by attribute using a matrix. The expert groups were not recorded, fully transcribed or analysed thematically as their purpose was to build on the outputs of the focus groups and capture the salient points for consideration, which was achievable through the matrix coding.

4. NARRATIVE SYNTHESIS

4.1. Objectives

The purpose of the focus and expert groups was to inform the selection of attributes and levels for the DCE. Achieving a balance between attributes of importance to the study population and attributes which will deliver impact to services and technology developers was the key consideration in selection. This led to the decision that a formal ranking or consensus method should not be used with either the focus or expert groups as a combined view of both was required. Instead, the approach adopted was a narrative synthesis developed for implementation reviews to enable

the outputs from the focus groups, expert groups and literature review to be synthesised against each potential attribute (Pope et al., 2007).

4.2. Methods

The approach taken in the narrative synthesis is outlined below, adapted from the key elements of synthesis identified by Pope and colleagues (2007):

Element of Synthesis	Approach Taken
**Developing a Theoretical Model**	Identification of a checklist of properties against which attributes can be considered to inform the selection process
**Developing a Preliminary Synthesis**	Tabulation against the checklist – to create a matrix of checklist criteria against attributes to enable visual comparison of perspective
**Exploring Relationships in the Data**	Conceptual mapping and triangulation against the checklist
**Assessing the Robustness of the Synthesis Product**	Critical reflection on the synthesis process
