# Supplementary material for: Young people’s preferences for the use of emerging technologies for asymptomatic regular chlamydia testing and management: a discrete choice experiment in England
Source: BMJ Open. 2019 Jan 29;9(1):e023663. doi: 10.1136/bmjopen-2018-023663 (PMC6352830; doi:10.1136/bmjopen-2018-023663)
Supplement: Supplementary file 2 [file bmjopen-2018-023663supp002.pdf]

Supplementary File 2 – DCE Questionnaire Introduction and Background Information

Developing Chlamydia Testing & Treatment Services

This survey is designed to find out more about your views on what is important to you in accessing services to get tested for chlamydia, and treatment if your test result is positive (i.e. you have chlamydia). This forms part of a research project being undertaken at the University of Warwick, please click here to access further information about the study and how your information will be used [insert url to participant information leaflet]. By continuing to complete the survey you are consenting for your responses to be used for research in accordance with the information set out in the study information leaflet. Your responses will help us understand what is important to young people to shape how services are developed in the future.

Please read through the following background information before completing the survey. It contains information about chlamydia and the terms that are used in the survey.

What is Chlamydia?

Chlamydia is the most common sexually transmitted infection in England and the majority of infections are found in young people aged 15-24. Both men and women can get chlamydia, but most people with chlamydia have no symptoms and do not know they have an infection. The test for chlamydia is usually a urine sample for men and a vaginal swab for women. Once diagnosed, chlamydia can be treated with a single dose (2 or 4 tablets) of an antibiotic called Azithromycin.

In England there is a national chlamydia screening programme which recommends that people aged 16-24 are tested for chlamydia annually or when they change their sexual partner.

What happens if I don't get treatment?

If left undiagnosed chlamydia can lead to serious health problems.

In women this includes a condition called Pelvic Inflammatory Disease, which is an infection of the womb, fallopian tubes or ovaries. This can cause severe pain and in some cases (around 1 in 10) lead to infertility or ectopic pregnancy (where the pregnancy occurs outside of the womb in the fallopian tube). If you are pregnant and have chlamydia you can pass it on to your baby when you give birth and this can lead to the baby getting an eye infection or pneumonia.

In men chlamydia can lead to a condition called epididymitis, which causes soreness and swelling in the scrotum.

For further information

If you would like further information on chlamydia and how to prevent it, or any of the other conditions described please visit:
http://www.nhs.uk/conditions/chlamydia/Pages/Introduction.aspx

About the Survey…

In completing the survey you will be presented with a number of choices for getting tested and treatment for chlamydia. Please consider each set of choices and indicate whether you prefer option A or option B or whether you would not test, that is, you would not choose option A or option B.

Each choice involves two different scenarios for testing and getting treatment and there are differences between how you test, how long you have to wait for the result, how accurate the test is, how you have a consultation to get treatment for chlamydia, how you access a healthcare professional to get that treatment and how you get your antibiotic.

The following sections provide you with an explanation of the process of getting tested and treatment and the key terms used in the survey:

The diagram below shows the stages up until you find out our result:

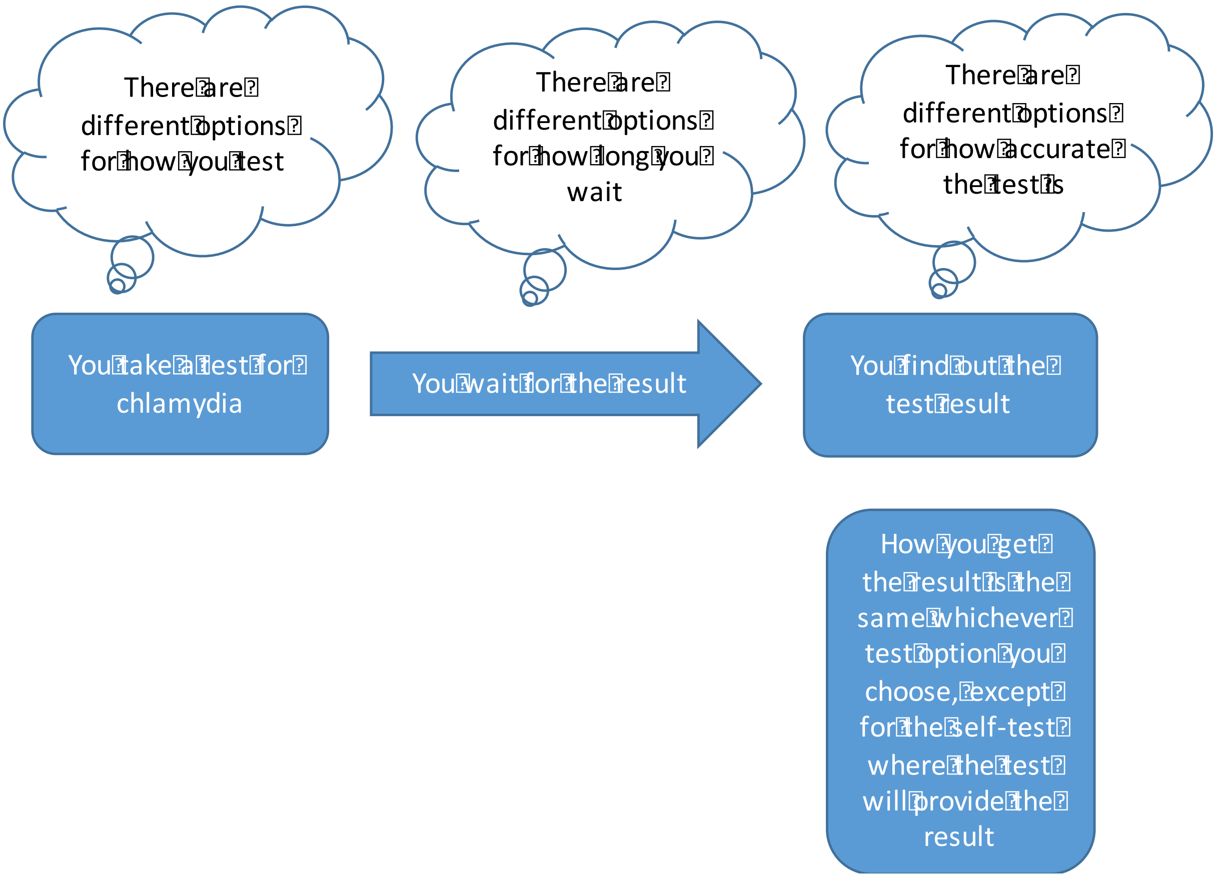

The following sections provide more information on how you test, how long you wait for the result and the accuracy of the test.

How you test for Chlamydia

This focuses on how you get your test, how the sample is taken and what happens to the sample once you've taken it. There are six options in the questionnaire, an explanation of these is provided below:

- **Self-Test -** Order a test kit online or collect one from a community location e.g. pharmacy or supermarket, provide the sample yourself and interpret the result yourself (like a pregnancy test). This is different to the self-sample options below because you interpret the result yourself rather than it being interpreted by a healthcare professional.
- **Self-Sample and post off for analysis -** Order a test kit online or collect one from a community location e.g. pharmacy or supermarket, provide the sample yourself and send the sample in the freepost envelope to the laboratory for analysis. This is different to the self-test option above because the test is interpreted by a healthcare professional.
- **Self-Sample and take to pharmacy for analysis -** Order a test kit online or collect one from a community location e.g. pharmacy or supermarket, provide the sample yourself and then take the sample to a pharmacy for analysis. This is different to the self-test option above because the test is interpreted by a

healthcare professional. Opening hours vary between pharmacies but many in towns and cities are open evenings and weekends as well as weekdays.

- **Self-Sample and take to your place of education/ workplace for analysis -** Order a test kit online or collect one from a community location e.g. pharmacy or supermarket, provide the sample yourself and take the sample to your place of education/ workplace for analysis by an outreach nurse. This is different to the self-test option above because the test is interpreted by a healthcare professional. Outreach services in education and workplaces are usually available one day a week.
- **Attend GP Practice, sample taken by a GP or Nurse -** Book an appointment at your GP practice with a GP or practice nurse. Your sample will be taken by the healthcare professional you see. GP practices are routinely open Monday to Friday 8am – 6.30pm.
- **Attend sexual health clinic, sample taken by a Doctor or Nurse -** Drop in or book an appointment with a doctor or nurse at a sexual health clinic. Your sample will be taken by the health care professional you see. Sexual Health Clinics are located in towns and cities, opening hours vary but clinics are generally open during the working day Monday to Friday, some evenings during the week and Saturdays.

Taking a sample yourself is straightforward and does not affect the accuracy of the test result.

Time to Result

How long it takes to get the test result. For the self-test option, this is how long it takes from you doing the test to you being able to read the result. For the other options this is how long it takes from you posting or dropping off your sample to when you are given your result. There are four options:

- 30 Mins
- 2 Hours
- 7 Days
- 14 Days

The time it takes to get the result does not affect the accuracy of the test.

Test Accuracy

This tells you how accurate your test result is. In this survey we want you to consider the likelihood of the test telling you that you don't have chlamydia when in fact you do. There are two options:

1. 2 in 100 people will be told that their test result is negative when they do have chlamydia
2. 5 in 100 people will be told their test result is negative when they do have chlamydia

Another way of describing this is shown below:

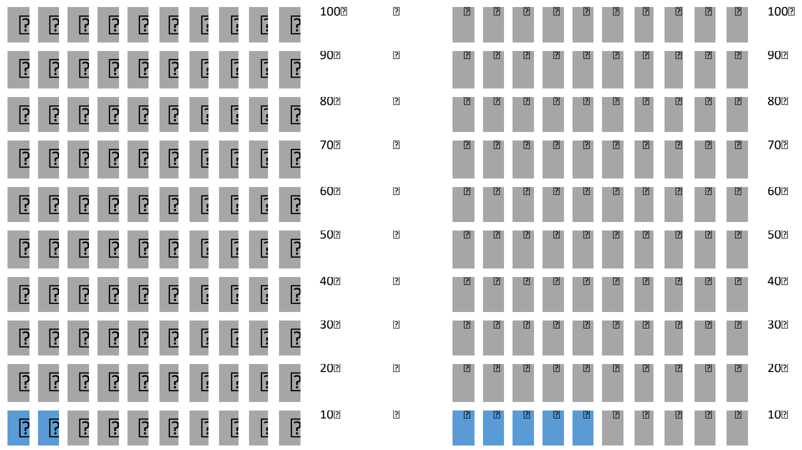

Each graph represents 100 people

= People who are told their test result is negative when they do have chlamydia

We would also like you to think about how you would choose to get treatment if your test result is positive. The diagram below shows the stages to get treatment:

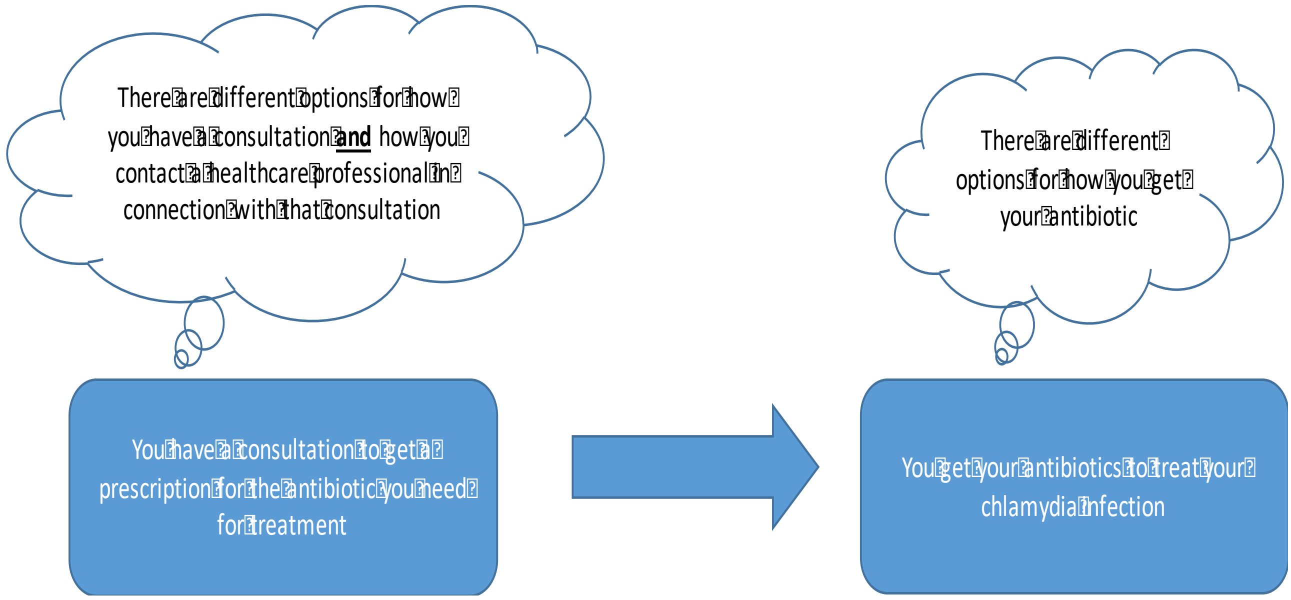

The following sections provide more information on how you get your treatment, how you contact a healthcare professional and how you get your antibiotics.

How you get your treatment

If your test result is positive there are a number of different options for you to get your treatment for chlamydia. There are four options in this survey:

- Complete an online consultation – this is available 24 hours a day, 7 days a week. You can access a healthcare professional if you need any help or advice between 9am and 5pm Monday to Friday.
- Have a consultation with a pharmacist - Opening hours vary between pharmacies but many in towns and cities are open evenings and weekends as well as weekdays.
- Have a consultation with a GP - GP practices are routinely open Monday to Friday 8am – 6.30pm.
- Have a consultation with a doctor or nurse at a Sexual Health Clinic - Sexual Health Clinics are located in towns and cities, opening hours vary but clinics are generally open during the working day Monday to Friday, some evenings during the week and Saturdays.

How you Contact a Healthcare Professional

New technology means that there are now more options for completing your consultation to get treatment, or in the case of the online consultation, accessing a healthcare professional for advice. There are four options within the questionnaire:

- Telephone – speak to a healthcare professional on the telephone.
- Instant Messaging – have a discussion with a healthcare professional via an instant messaging service online
- Email – have a discussion with a healthcare professional via email. During opening hours you will receive a response within 2 hours, and for emails sent out of hours you will receive a response the next day.
- Face-to-face - have a face-to-face discussion with a healthcare professional.

How you get your antibiotics

Once you've completed your consultation you need to get your antibiotic tablets. In this survey there are four options available to you:

- Deliver to home address – provide your home address for your antibiotic to be posted to you. Your antibiotic will arrive within 1-2 working days.
- Deliver to collection point – nominate an address other than your home address, for example a friend's address or a collection point for your antibiotic to be posted to. Your antibiotic will arrive within 1-2 working days.
- Collect from Pharmacy – attend a pharmacy of your choice with your prescription and the pharmacist gives you the antibiotic tablets
- Collect from Sexual Health Clinic – attend a sexual health clinic and the doctor or nurse gives you the antibiotic tablets

In all of the choices available to you there are a number of things that you should assume are the same:

- The service is provided to you free of charge although there may be a cost to you, for example in making phone calls, accessing the internet, or taking time off work to go to an appointment.

- The healthcare professional who is providing your treatment is trained to be able to provide the service.
- How you get your results is the same whichever option you choose, except for the self-test where the test will provide the result.
- Your personal data is managed securely in whichever option you choose

More choices for an option does not mean that it is more or less important, it just reflects that there are more choices within the scenarios you are being asked to consider.
