# Supplementary material for: Young people’s preferences for the use of emerging technologies for asymptomatic regular chlamydia testing and management: a discrete choice experiment in England
Source: BMJ Open. 2019 Jan 29;9(1):e023663. doi: 10.1136/bmjopen-2018-023663 (PMC6352830; doi:10.1136/bmjopen-2018-023663)
Supplement: Supplementary file 3 [file bmjopen-2018-023663supp003.pdf]

Supplementary File 3 – DCE Results

Part 1 - Demographic Characteristics for Sub-Group Analysis

Demographic Characteristic	n	%	National %[1]
Age			
16	8	1%	10%
17	113	9%	10%
18	294	24%	11%
19	183	15%	11%
20	132	11%	11%
21	91	7%	11%
22	162	13%	11%
23	135	11%	12%
24	112	9%	12%
Total	1230	100%	99.00%
**Gender**			
Male	607	49%	51%
Female	623	51%	49%
Total	1230	100%	100%
**Ethnicity**			
White – English, Welsh, Scottish, Northern Irish, British	932	76%	80%
White – Irish	7	1%	1%
White – Gypsy or Irish Traveller	1	0%	0%
White – Any other white background	37	3%	5%
Mixed/ Multiple Ethnic Groups – White & Black Caribbean	10	1%	1%
Mixed/ Multiple Ethnic Groups – White & Black African	6	0%	0%
Mixed/ Multiple Ethnic Groups – White & Asian	13	1%	1%
Mixed/ Multiple Ethnic Groups – Any other mixed/ multiple ethnic background	8	1%	1%
Asian/ Asian British – Indian	50	4%	3%
Asian/ Asian British - Pakistani	38	3%	2%
Asian/ Asian British - Bangladeshi	18	1%	1%
Asian/ Asian British - Chinese	22	2%	1%
Asian/ Asian British – Any other Asian background	21	2%	2%
Black/ African/ Caribbean/ Black British – African	26	2%	2%

[1] - National Data for Age and Region taken from ONS Mid-Year Population Estimates 2015 (ONS, 2016b). Percentages derived from the total 16-24 population. National Data for Ethnicity taken from Census 2011 data, percentages derived from total England population (ONS, 2012). Percentages may not sum due to rounding

Demographic Characteristic	n	%	National %[1]
Black/ African/ Caribbean/ Black British – Caribbean	10	1%	1%
Black/ African/ Caribbean/ Black British – Any other black/ African/ Caribbean background	2	0%	1%
Other – Arab	5	0%	0%
Other – Any other ethnic group	9	1%	1%
I would prefer not to say	15	1%	0%
Total	1230	100%	103.00%
**Region**			
East Midlands	117	10%	9%
London	225	18%	15%
North East	74	6%	5%
North West	123	10%	13%
Eastern	55	4%	10%
South East	222	18%	16%
South West	143	12%	10%
West Midlands	144	12%	11%
Yorkshire and The Humber	113	9%	11%
I would prefer not to say	14	1%	0%
Total	1230	100%	100.00%
**Sexual Preference**			
Heterosexual (partner of opposite sex)	1030	84%	
Homosexual (partner of same sex) & Male	13	1%	
Homosexual (partner of same sex) & Female	39	3%	
Bisexual (partner of either sex)	98	8%	
I would prefer not to say	50	4%	
Total	1230	100%	
**Previous STI Test**			
Yes	393	32%	
No	790	64%	
I would prefer not to say	47	4%	
Total	1230	100%	
**Relationship Status**			
Single	615	50%	
In a non-sexual relationship with one person	36	3%	
In a non-sexual relationship with more than one person	2	0%	
In a sexual relationship with one person	512	42%	
In a sexual relationship with more than one person	36	3%	
I would prefer not to say	29	2%	
Total	1230	100%	

Part 2 - Validity Checks

Table 2.1 – Comparison of Full Dataset Coefficients and Odds Ratios for the Full Dataset for Dataset including Choice Set 1 and Dataset including Choice Set 25. Data highlighted in blue indicate results which are not statistically significant.

	**Dataset Including Choice Set 1, *n=1,230***						**Dataset Including Choice Set 25, *n=1,230***					
	**Coefficient**	**Lower 95% CI**	**Upper 95% CI**	**Odds Ratio**	**Lower 95% CI**	**Upper 95% CI**	**Coefficient**	**Lower 95% CI**	**Upper 95% CI**	**Odds Ratio**	**Lower 95% CI**	**Upper 95% CI**
**How you Test**												
Self-Test	0.498	0.432	0.564	1.646	1.540	1.758	0.481	0.415	0.547	1.618	1.514	1.729
Post	0.308	0.239	0.376	1.360	1.270	1.456	0.306	0.239	0.373	1.358	1.271	1.452
Pharmacy	0.116	0.056	0.175	1.123	1.057	1.192	0.144	0.085	0.203	1.155	1.088	1.226
Education/ Work	-0.206	-0.266	-0.146	0.814	0.766	0.864	-0.197	-0.257	-0.137	0.821	0.773	0.872
GP Practice	0.011	-0.050	0.073	1.011	0.951	1.075	0.019	-0.042	0.081	1.019	0.959	1.084
Sexual Health Clinic	0.000	-	-	1.000	-	-	0.000	-	-	1.000	-	-
**Time to Result**												
30 Mins	0.579	0.524	0.633	1.784	1.688	1.884	0.591	0.537	0.645	1.806	1.711	1.906
2 Hours	0.340	0.283	0.398	1.406	1.328	1.488	0.338	0.281	0.395	1.402	1.324	1.485
7 Days	0.000	-	-	1.000	-	-	0.000	-	-	1.000	-	-
14 Days	-0.149	-0.200	-0.099	0.861	0.819	0.906	-0.148	-0.198	-0.098	0.862	0.820	0.907
**Accuracy**												
2 in 100 False Negative	1.192	1.157	1.226	3.292	3.179	3.409	1.176	1.141	1.212	3.242	3.130	3.359
5 in 100 False Negative	0.000	-	-	1.000	-	-	0.000	-	-	1.000	-	-

	Dataset Including Choice Set 1, *n=1,230*						Dataset Including Choice Set 25, *n=1,230*					
	**Coefficient**	**Lower 95% CI**	**Upper 95% CI**	**Odds Ratio**	**Lower 95% CI**	**Upper 95% CI**	**Coefficient**	**Lower 95% CI**	**Upper 95% CI**	**Odds Ratio**	**Lower 95% CI**	**Upper 95% CI**
**Consultation Method**												
Online Consultation	0.185	0.133	0.236	1.203	1.142	1.266	0.192	0.140	0.245	1.212	1.150	1.277
Pharmacy Consultation	0.150	0.099	0.202	1.162	1.104	1.224	0.147	0.095	0.199	1.158	1.100	1.220
GP Consultation	0.146	0.095	0.197	1.157	1.100	1.218	0.168	0.116	0.220	1.183	1.123	1.246
Sexual Health Clinic Consultation	0.000	-	-	1.000	-	-	0.000	-	-	1.000	-	-
**Access to Healthcare Professional**												
Phone	-0.058	-0.108	-0.008	0.943	0.897	0.992	-0.052	-0.102	-0.002	0.949	0.903	0.998
Instant Messenger	0.020	-0.029	0.070	1.020	0.971	1.072	0.027	-0.023	0.076	1.027	0.977	1.079
Email	0.027	-0.023	0.076	1.027	0.978	1.079	0.047	-0.002	0.096	1.048	0.998	1.101
Face to Face	0.000	-	-	1.000	-	-	0.000	-	-	1.000	-	-
**How you get Antibiotics**												
Post to Home	0.038	-0.013	0.089	1.039	0.987	1.093	0.011	-0.041	0.063	1.011	0.960	1.065
Post to Collection Point	0.052	0.002	0.101	1.053	1.002	1.106	0.030	-0.020	0.081	1.031	0.980	1.085
Collect from Pharmacy	0.091	0.038	0.143	1.095	1.039	1.154	0.072	0.018	0.126	1.075	1.018	1.134
Collect from Sexual Health Clinic	0.000	-	-	1.000	-	-	0.000	-	-	1.000	-	-
**Choice**												
Option A	0.000	-	-	1.000	-	-	0.000	-	-	1.000	-	-
Option B	-0.341	-0.381	-0.302	0.711	0.683	0.740	-0.360	-0.400	-0.320	0.698	0.670	0.726
I would not test	-1.697	-1.797	-1.596	0.183	0.166	0.203	-1.682	-1.781	-1.583	0.186	0.168	0.205

Table 2.2 – Coefficients and Odds Ratios for the Full Dataset and the Dataset Excluding Different Responses to Choice Sets 1 and 25. Data highlighted in blue indicate results which are not statistically significant.

	**Full Dataset *n=1,230***						**Excl Incorrect Answer to Validity Check *n=912***					
	**Coefficient**	**Lower 95% CI**	**Upper 95% CI**	**Odds Ratio**	**Lower 95% CI**	**Upper 95% CI**	**Coefficient**	**Lower 95% CI**	**Upper 95% CI**	**Odds Ratio**	**Lower 95% CI**	**Upper 95% CI**
**How you Test**												
Self-Test	0.481	0.415	0.547	1.618	1.514	1.729	0.507	0.429	0.584	1.660	1.535	1.794
Post	0.306	0.239	0.373	1.358	1.271	1.452	0.333	0.254	0.411	1.395	1.290	1.508
Pharmacy	0.144	0.085	0.203	1.155	1.088	1.226	0.124	0.054	0.194	1.132	1.056	1.214
Education/ Work	-0.197	-0.257	-0.137	0.821	0.773	0.872	-0.253	-0.323	-0.183	0.777	0.724	0.833
GP Practice	0.019	-0.042	0.081	1.019	0.959	1.084	0.019	-0.053	0.091	1.019	0.948	1.095
Sexual Health Clinic	0.000	-	-	1.000	-	-	0.000	-	-	1.000	-	-
**Time to Result**												
30 Mins	0.591	0.537	0.645	1.806	1.711	1.906	0.573	0.510	0.637	1.774	1.665	1.890
2 Hours	0.338	0.281	0.395	1.402	1.324	1.485	0.335	0.268	0.402	1.397	1.307	1.494
7 Days	0.000	-	-	1.000	-	-	0.000	-	-	1.000	-	-
14 Days	-0.148	-0.198	-0.098	0.862	0.820	0.907	-0.148	-0.177	-0.236	-0.117	0.790	0.889
**Accuracy**												
2 in 100 False Negative	1.176	1.141	1.212	3.242	3.130	3.359	1.323	1.282	1.364	3.755	3.602	3.913
5 in 100 False Negative	0.000	-	-	1.000	-	-	0.000	-	-	1.000	-	-
**Consultation Method**												
Online Consultation	0.192	0.140	0.245	1.212	1.150	1.277	0.197	0.136	0.258	1.218	1.145	1.294
Pharmacy Consultation	0.147	0.095	0.199	1.158	1.100	1.220	0.173	0.112	0.234	1.189	1.119	1.264
GP Consultation	0.168	0.116	0.220	1.183	1.123	1.246	0.179	0.119	0.240	1.196	1.126	1.271
Sexual Health Clinic Consultation	0.000	-	-	1.000	-	-	0.000	-	-	1.000	-	-

	Full Dataset *n=1,230*						Excl Incorrect Answer to Validity Check *n=912*					
	**Coefficient**	**Lower 95% CI**	**Upper 95% CI**	**Odds Ratio**	**Lower 95% CI**	**Upper 95% CI**	**Coefficient**	**Lower 95% CI**	**Upper 95% CI**	**Odds Ratio**	**Lower 95% CI**	**Upper 95% CI**
**Access to Healthcare Professional**												
Phone	-0.052	-0.102	-0.002	0.949	0.903	0.998	-0.084	-0.143	-0.026	0.919	0.867	0.975
Instant Messenger	0.027	-0.023	0.076	1.027	0.977	1.079	-0.026	-0.084	0.031	0.974	0.919	1.032
Email	0.047	-0.002	0.096	1.048	0.998	1.101	-0.005	-0.063	0.052	0.995	0.939	1.054
Face to Face	0.000	-	-	1.000	-	-	0.000	-	-	1.000	-	-
**How you get Antibiotics**												
Post to Home	0.011	-0.041	0.063	1.011	0.960	1.065	0.014	-0.047	0.075	1.014	0.954	1.078
Post to Collection Point	0.030	-0.020	0.081	1.031	0.980	1.085	0.023	-0.036	0.083	1.024	0.964	1.086
Collect from Pharmacy	0.072	0.018	0.126	1.075	1.018	1.134	0.065	0.001	0.128	1.067	1.001	1.137
Collect from Sexual Health Clinic	0.000	-	-	1.000	-	-	0.000	-	-	1.000	-	-
**Choice**												
Option A	0.000	-	-	1.000	-	-	0.000	-	-	1.000	-	-
Option B	-0.360	-0.400	-0.320	0.698	0.670	0.726	-0.327	-0.374	-0.279	0.721	0.688	0.756
I would not test	-1.682	-1.781	-1.583	0.186	0.168	0.205	-1.674	-1.790	-1.557	0.188	0.167	0.211

Table 2.3 – Coefficient and Odds Ratios for the Full Dataset and the Dataset Containing Five Minutes or Longer to Complete. Data highlighted in blue indicate results which are not statistically significant.

	**Full Dataset** ***n=1,230***						**Data Excluding Responses taking less than 5 min to complete** ***n= 997***					
	**Coefficient**	**Lower 95% CI**	**Upper 95% CI**	**Odds Ratio**	**Lower 95% CI**	**Upper 95% CI**	**Coefficient**	**Lower 95% CI**	**Upper 95% CI**	**Odds Ratio**	**Lower 95% CI**	**Upper 95% CI**
**How you Test**												
Self-Test	0.481	0.415	0.547	1.618	1.514	1.729	0.542	0.467	0.616	1.719	1.595	1.852
Post	0.306	0.239	0.373	1.358	1.271	1.452	0.344	0.269	0.419	1.411	1.309	1.521
Pharmacy	0.144	0.085	0.203	1.155	1.088	1.226	0.168	0.101	0.234	1.183	1.106	1.264
Education/ Work	-0.197	-0.257	-0.137	0.821	0.773	0.872	-0.245	-0.312	-0.177	0.783	0.732	0.837
GP Practice	0.019	-0.042	0.081	1.019	0.959	1.084	-0.008	-0.077	0.061	0.992	0.925	1.062
Sexual Health Clinic	0.000	-	-	1.000	-	-	0.000	-	-	1.000	-	-
**Time to Result**												
30 Mins	0.591	0.537	0.645	1.806	1.711	1.906	0.642	0.581	0.703	1.900	1.788	2.019
2 Hours	0.338	0.281	0.395	1.402	1.324	1.485	0.372	0.308	0.436	1.451	1.361	1.547
7 Days	0.000	-	-	1.000	-	-	0.000	-	-	1.000	-	-
14 Days	-0.148	-0.198	-0.098	0.862	0.820	0.907	-0.147	-0.204	-0.091	0.863	0.816	0.913
**Accuracy**												
2 in 100 False Negative	1.176	1.141	1.212	3.242	3.130	3.359	1.308	1.269	1.348	3.700	3.556	3.850
5 in 100 False Negative	0.000	-	-	1.000	-	-	0.000	-	-	1.000	-	-
**Consultation Method**												
Online Consultation	0.192	0.140	0.245	1.212	1.150	1.277	0.217	0.159	0.276	1.243	1.172	1.318
Pharmacy Consultation	0.147	0.095	0.199	1.158	1.100	1.220	0.160	0.102	0.219	1.174	1.108	1.245
GP Consultation	0.168	0.116	0.220	1.183	1.123	1.246	0.171	0.113	0.229	1.187	1.120	1.258
Sexual Health Clinic Consultation	0.000	-	-	1.000	-	-	0.000	-	-	1.000	-	-
**Access to Healthcare Professional**												
Phone	-0.052	-0.102	-0.002	0.949	0.903	0.998	-0.042	-0.098	0.014	0.959	0.906	1.014
Instant Messenger	0.027	-0.023	0.076	1.027	0.977	1.079	0.043	-0.013	0.098	1.044	0.987	1.103
Email	0.047	-0.002	0.096	1.048	0.998	1.101	0.042	-0.013	0.097	1.043	0.987	1.102
Face to Face	0.000	-	-	1.000	-	-	0.000	-	-	1.000	-	-

	Full Dataset *n=1,230*						Data Excluding Responses taking less than 5 min to complete *n= 997*					
	**Coefficient**	**Lower 95% CI**	**Upper 95% CI**	**Odds Ratio**	**Lower 95% CI**	**Upper 95% CI**	**Coefficient**	**Lower 95% CI**	**Upper 95% CI**	**Odds Ratio**	**Lower 95% CI**	**Upper 95% CI**
**How you get Antibiotics**												
Post to Home	0.011	-0.041	0.063	1.011	0.960	1.065	0.001	-0.057	0.060	1.001	0.944	1.062
Post to Collection Point	0.030	-0.020	0.081	1.031	0.980	1.085	0.013	-0.044	0.070	1.013	0.957	1.072
Collect from Pharmacy	0.072	0.018	0.126	1.075	1.018	1.134	0.085	0.024	0.145	1.089	1.025	1.157
Collect from Sexual Health Clinic	0.000	-	-	1.000	-	-	0.000	-	-	1.000	-	-
**Choice**												
Option A	0.000	-	-	1.000	-	-	0.000	-	-	1.000	-	-
Option B	-0.360	-0.400	-0.320	0.698	0.670	0.726	-0.368	-0.413	-0.323	0.692	0.662	0.724
I would not test	-1.682	-1.781	-1.583	0.186	0.168	0.205	-1.776	-1.889	-1.662	0.169	0.151	0.190

Table 2.4 – Comparison of Full Dataset and Dataset Excluding Opt Out Responses. Data highlighted in blue indicate results which are not statistically significant.

	**Full Dataset *n=29,520 Choice Responses***						**Removal of 'I would not test' Data *n=27,727 Choice Responses***					
	**Coefficient**	**Lower 95% CI**	**Upper 95% CI**	**Odds Ratio**	**Lower 95% CI**	**Upper 95% CI**	**Coefficient**	**Lower 95% CI**	**Upper 95% CI**	**Odds Ratio**	**Lower 95% CI**	**Upper 95% CI**
**How you Test**												
Self-Test	0.481	0.415	0.547	1.618	1.514	1.729	0.501	0.433	0.568	1.650	1.542	1.765
Post	0.306	0.239	0.373	1.358	1.271	1.452	0.320	0.252	0.387	1.377	1.287	1.473
Pharmacy	0.144	0.085	0.203	1.155	1.088	1.226	0.152	0.092	0.212	1.164	1.096	1.236
Education/ Work	-0.197	-0.257	-0.137	0.821	0.773	0.872	-0.202	-0.262	-0.141	0.817	0.769	0.868
GP Practice	0.019	-0.042	0.081	1.019	0.959	1.084	0.027	-0.035	0.089	1.027	0.965	1.093
Sexual Health Clinic	0.000	-	-	1.000	-	-	0.000	-	-	1.000	-	-
**Time to Result**												
30 Mins	0.591	0.537	0.645	1.806	1.711	1.906	0.612	0.558	0.667	1.845	1.746	1.949
2 Hours	0.338	0.281	0.395	1.402	1.324	1.485	0.350	0.292	0.408	1.419	1.339	1.503
7 Days	0.000	-	-	1.000	-	-	0.000	-	-	1.000	-	-
14 Days	-0.148	-0.198	-0.098	0.862	0.820	0.907	-0.145	-0.196	-0.094	0.865	0.822	0.910
**Accuracy**												
2 in 100 False Negative	1.176	1.141	1.212	3.242	3.130	3.359	1.205	1.170	1.241	3.338	3.221	3.460
5 in 100 False Negative	0.000	-	-	1.000	-	-	0.000	-	-	1.000	-	-
**Consultation Method**												
Online Consultation	0.192	0.140	0.245	1.212	1.150	1.277	0.200	0.147	0.253	1.222	1.159	1.288
Pharmacy Consultation	0.147	0.095	0.199	1.158	1.100	1.220	0.159	0.106	0.211	1.172	1.112	1.235
GP Consultation	0.168	0.116	0.220	1.183	1.123	1.246	0.174	0.122	0.227	1.190	1.130	1.254
Sexual Health Clinic Consultation	0.000	-	-	1.000	-	-	0.000	-	-	1.000	-	-
**Access to Healthcare Professional**												
Phone	-0.052	-0.102	-0.002	0.949	0.903	0.998	-0.046	-0.097	0.004	0.955	0.908	1.004
Instant Messenger	0.027	-0.023	0.076	1.027	0.977	1.079	0.029	-0.021	0.079	1.029	0.979	1.082
Email	0.047	-0.002	0.096	1.048	0.998	1.101	0.050	0.000	0.100	1.051	1.000	1.105
Face to Face	0.000	-	-	1.000	-	-	0.000	-	-	1.000	-	-

	Full Dataset *n=29,520 Choice Responses*						Removal of 'I would not test' Data *n=27,727 Choice Responses*					
	**Coefficient**	**Lower 95% CI**	**Upper 95% CI**	**Odds Ratio**	**Lower 95% CI**	**Upper 95% CI**	**Coefficient**	**Lower 95% CI**	**Upper 95% CI**	**Odds Ratio**	**Lower 95% CI**	**Upper 95% CI**
**How you get Antibiotics**												
Post to Home	0.011	-0.041	0.063	1.011	0.960	1.065	0.012	-0.041	0.065	1.012	0.960	1.067
Post to Collection Point	0.030	-0.020	0.081	1.031	0.980	1.085	0.035	-0.016	0.086	1.036	0.984	1.090
Collect from Pharmacy	0.072	0.018	0.126	1.075	1.018	1.134	0.076	0.021	0.131	1.079	1.022	1.139
Collect from Sexual Health Clinic	0.000	-	-	1.000	-	-	0.000	-	-	1.000	-	-
**Choice**												
Option A	0.000	-	-	1.000	-	-	0.000	-	-	1.000	-	-
Option B	-0.360	-0.400	-0.320	0.698	0.670	0.726	-0.368	-0.409	-0.327	0.692	0.664	0.721
I would not test	-1.682	-1.781	-1.583	0.186	0.168	0.205	-	-	-	1.000		

Part 3 – DCE Results

(Cells highlighted in blue indicate result which is not statistically significant)

Table 3.1- Full Dataset Coefficients, Odds Ratios and Respective 95% Confidence Interval

	**Full Dataset *n=1,230***					
	**Coefficient**	**Lower 95% CI**	**Upper 95% CI**	**Odds Ratio**	**Lower 95% CI**	**Upper 95% CI**
**How you Test**						
Self-Test	0.481	0.415	0.547	1.618	1.514	1.729
Post	0.306	0.239	0.373	1.358	1.271	1.452
Pharmacy	0.144	0.085	0.203	1.155	1.088	1.226
Education/ Work	-0.197	-0.257	-0.137	0.821	0.773	0.872
GP Practice	0.019	-0.042	0.081	1.019	0.959	1.084
Sexual Health Clinic	0.000	-	-	1.000	-	-
**Time to Result**						
30 Mins	0.591	0.537	0.645	1.806	1.711	1.906
2 Hours	0.338	0.281	0.395	1.402	1.324	1.485
7 Days	0.000	-	-	1.000	-	-
14 Days	-0.148	-0.198	-0.098	0.862	0.820	0.907
**Accuracy**						
2 in 100 False Negative	1.176	1.141	1.212	3.242	3.130	3.359
5 in 100 False Negative	0.000	-	-	1.000	-	-
**Consultation Method**						
Online Consultation	0.192	0.140	0.245	1.212	1.150	1.277
Pharmacy Consultation	0.147	0.095	0.199	1.158	1.100	1.220
GP Consultation	0.168	0.116	0.220	1.183	1.123	1.246
Sexual Health Clinic Consultation	0.000	-	-	1.000	-	-
**Access to Healthcare Professional**						
Phone	-0.052	-0.102	-0.002	0.949	0.903	0.998
Instant Messenger	0.027	-0.023	0.076	1.027	0.977	1.079
Email	0.047	-0.002	0.096	1.048	0.998	1.101
Face to Face	0.000	-	-	1.000	-	-
**How you get Antibiotics**						
Post to Home	0.011	-0.041	0.063	1.011	0.960	1.065
Post to Collection Point	0.030	-0.020	0.081	1.031	0.980	1.085
Collect from Pharmacy	0.072	0.018	0.126	1.075	1.018	1.134
Collect from Sexual Health Clinic	0.000	-	-	1.000	-	-

Table 3.2 - Gender Subgroup Analysis Coefficients and Odds Ratios with Associated 95% Confidence Intervals

	**Males *n=607***						**Females *n=623***					
	**Coefficient**	**Lower 95% CI**	**Upper 95% CI**	**Odds Ratio**	**Lower 95% CI**	**Upper 95% CI**	**Coefficient**	**Lower 95% CI**	**Upper 95% CI**	**Odds Ratio**	**Lower 95% CI**	**Upper 95% CI**
**How you Test**												
Self-Test	0.438	0.344	0.531	1.549	1.410	1.701	0.526	0.432	0.620	1.693	1.541	1.860
Post	0.269	0.174	0.363	1.308	1.190	1.438	0.344	0.250	0.439	1.411	1.284	1.551
Pharmacy	0.123	0.039	0.208	1.131	1.040	1.231	0.165	0.081	0.249	1.180	1.085	1.283
Education/ Work	-0.084	-0.169	0.001	0.919	0.845	1.001	-0.310	-0.395	-0.225	0.733	0.674	0.798
GP Practice	0.048	-0.039	0.135	1.049	0.962	1.145	-0.010	-0.097	0.077	0.990	0.908	1.080
Sexual Health Clinic	0.000	-	-	1.000	-	-	0.000	-	-	1.000	-	-
**Time to Result**												
30 Mins	0.603	0.527	0.680	1.828	1.694	1.973	0.580	0.503	0.656	1.786	1.654	1.928
2 Hours	0.311	0.230	0.392	1.365	1.259	1.479	0.366	0.285	0.447	1.442	1.329	1.563
7 Days	0.000	-	-	1.000	-	-	0.000	-	-	1.000	-	-
14 Days	-0.170	-0.241	-0.098	0.844	0.786	0.906	-0.127	-0.198	-0.056	0.881	0.820	0.945
**Accuracy**												
2 in 100 False Negative	1.082	1.032	1.132	2.951	2.807	3.101	1.273	1.223	1.322	3.570	3.396	3.753
5 in 100 False Negative	0.000	-	-	1.000	-	-	0.000	-	-	1.000	-	-
**Consultation Method**												
Online Consultation	0.171	0.097	0.245	1.187	1.102	1.278	0.214	0.140	0.288	1.239	1.151	1.334
Pharmacy Consultation	0.100	0.027	0.173	1.106	1.028	1.189	0.194	0.121	0.268	1.215	1.129	1.307
GP Consultation	0.159	0.085	0.232	1.172	1.089	1.262	0.178	0.104	0.251	1.194	1.110	1.285
Sexual Health Clinic Consultation	0.000	-	-	1.000	-	-	0.000	-	-	1.000	-	-
**Access to Healthcare Professional**												
Phone	-0.065	-0.136	0.005	0.937	0.873	1.005	-0.039	-0.110	0.032	0.962	0.896	1.032
Instant Messenger	-0.006	-0.076	0.063	0.994	0.927	1.065	0.060	-0.010	0.130	1.062	0.990	1.139
Email	0.033	-0.036	0.103	1.034	0.965	1.108	0.060	-0.009	0.130	1.062	0.991	1.139
Face to Face	0.000	-	-	1.000	-	-	0.000	-	-	1.000	-	-
**How you get Antibiotics**												
Post to Home	-0.004	-0.078	0.070	0.996	0.925	1.072	0.027	-0.046	0.101	1.028	0.955	1.106
Post to Collection Point	0.079	0.007	0.151	1.082	1.007	1.163	-0.017	-0.089	0.055	0.983	0.915	1.056
Collect from Pharmacy	0.062	-0.014	0.138	1.064	0.986	1.149	0.083	0.007	0.160	1.087	1.007	1.173
Collect from Sexual Health Clinic	0.000	-	-	1.000	-	-	0.000	-	-	1.000	-	-

Table 3.3 - Age Range Subgroup Analysis Coefficients and Odds Ratios, including Respective 95% Confidence Intervals

	16-18 *n=415*						19-21 *n=406*						22-24 *n=409*					
	**Coeffi-cient**	**Lower 95% CI**	**Upper 95% CI**	**Odds Ratio**	**Lower 95% CI**	**Upper 95% CI**	**Coeffi-cient**	**Lower 95% CI**	**Upper 95% CI**	**Odds Ratio**	**Lower 95% CI**	**Upper 95% CI**	**Coeffi-cient**	**Lower 95% CI**	**Upper 95% CI**	**Odds Ratio**	**Lower 95% CI**	**Upper 95% CI**
**How you Test**																		
Self-Test	0.509	0.395	0.623	1.664	1.485	1.865	0.297	0.182	0.412	1.346	1.200	1.510	0.639	0.523	0.755	1.895	1.687	2.128
Post	0.390	0.275	0.505	1.477	1.316	1.657	0.129	0.013	0.245	1.138	1.013	1.277	0.397	0.281	0.513	1.487	1.324	1.670
Pharmacy	0.239	0.137	0.342	1.270	1.147	1.408	0.021	-0.082	0.124	1.021	0.921	1.132	0.170	0.067	0.273	1.185	1.069	1.314
Education/ Work	-0.218	-0.322	-0.114	0.804	0.725	0.892	-0.215	-0.319	-0.112	0.806	0.727	0.894	-0.160	-0.265	-0.056	0.852	0.768	0.945
GP Practice	0.081	-0.025	0.187	1.085	0.975	1.206	0.035	-0.072	0.141	1.035	0.931	1.152	-0.061	-0.168	0.046	0.941	0.846	1.047
Sexual Health Clinic	0.000	-	-	1.000	-	-	0.000	-	-	1.000	-	-	0.000	-	-	1.000	-	-
**Time to Result**																		
30 Mins	0.567	0.474	0.661	1.763	1.606	1.936	0.636	0.542	0.730	1.888	1.719	2.074	0.572	0.478	0.666	1.772	1.613	1.947
2 Hours	0.282	0.183	0.381	1.326	1.201	1.464	0.437	0.338	0.536	1.548	1.402	1.709	0.295	0.196	0.395	1.344	1.216	1.484
7 Days	0.000	-	-	1.000	-	-	0.000	-	-	1.000	-	-	0.000	-	-	1.000	-	-
14 Days	-0.130	-0.217	-0.044	0.878	0.805	0.957	-0.125	-0.213	-0.038	0.882	0.808	0.963	-0.189	-0.277	-0.102	0.828	0.758	0.903
**Accuracy**																		
2 in 100 False Negative	1.196	1.135	1.257	3.307	3.112	3.514	1.151	1.090	1.212	3.161	2.974	3.361	1.188	1.127	1.250	3.282	3.086	3.489
5 in 100 False Negative	0.000	-	-	1.000	-	-	0.000	-	-	1.000	-	-	0.000	-	-	1.000	-	-
**Consultation Method**																		
Online Consultation	0.171	0.081	0.262	1.187	1.085	1.299	0.182	0.092	0.273	1.200	1.096	1.314	0.226	0.135	0.317	1.253	1.144	1.373
Pharmacy Consultation	0.154	0.065	0.244	1.167	1.067	1.276	0.190	0.100	0.279	1.209	1.105	1.322	0.098	0.007	0.188	1.102	1.007	1.206
GP Consultation	0.246	0.157	0.335	1.279	1.170	1.398	0.133	0.043	0.223	1.142	1.044	1.250	0.125	0.035	0.215	1.133	1.036	1.240
Sexual Health Clinic Consultation	0.000	-	-	1.000	-	-	0.000	-	-	1.000	-	-	0.000	-	-	1.000	-	-

	16-18 *n=415*						19-21 *n=406*						22-24 *n=409*					
	**Coeffi-cient**	**Lower 95% CI**	**Upper 95% CI**	**Odds Ratio**	**Lower 95% CI**	**Upper 95% CI**	**Coeffi-cient**	**Lower 95% CI**	**Upper 95% CI**	**Odds Ratio**	**Lower 95% CI**	**Upper 95% CI**	**Coeffi-cient**	**Lower 95% CI**	**Upper 95% CI**	**Odds Ratio**	**Lower 95% CI**	**Upper 95% CI**
**Access to Healthcare Professional**																		
Phone	0.001	-0.085	0.087	1.001	0.918	1.091	-0.104	-0.190	-0.017	0.902	0.827	0.983	-0.055	-0.142	0.031	0.946	0.868	1.032
Instant Messenger	0.102	0.017	0.188	1.108	1.017	1.207	-0.024	-0.109	0.062	0.977	0.897	1.064	0.001	-0.085	0.087	1.001	0.918	1.091
Email	0.075	-0.009	0.160	1.078	0.991	1.174	0.037	-0.048	0.122	1.038	0.954	1.130	0.029	-0.056	0.115	1.030	0.945	1.122
Face to Face	0.000	-	-	1.000	-	-	0.000	-	-	1.000	-	-	0.000	-	-	1.000	-	-
**How you get Antibiotics**																		
Post to Home	-0.024	-0.114	0.066	0.977	0.893	1.068	0.010	-0.081	0.100	1.010	0.922	1.106	0.047	-0.043	0.137	1.048	0.957	1.147
Post to Collection Point	0.010	-0.078	0.097	1.010	0.925	1.102	0.035	-0.053	0.123	1.036	0.948	1.131	0.046	-0.042	0.135	1.047	0.959	1.144
Collect from Pharmacy	0.039	-0.054	0.132	1.039	0.947	1.141	0.101	0.008	0.195	1.106	1.008	1.215	0.077	-0.017	0.171	1.080	0.983	1.186
Collect from Sexual Health Clinic	0.000	-	-	1.000	-	-	0.000	-	-	1.000	-	-	0.000	-	-	1.000	-	-

Table 3.4 - Relationship Status Subgroup Analysis Coefficients and Odds Ratios, including Respective 95% Confidence Intervals

	Single *n=615*						Sexual Relationship with One Partner *n=512*					
	**Coefficient**	**Lower 95% CI**	**Upper 95% CI**	**Odds Ratio**	**Lower 95% CI**	**Upper 95% CI**	**Coefficient**	**Lower 95% CI**	**Upper 95% CI**	**Odds Ratio**	**Lower 95% CI**	**Upper 95% CI**
**How you Test**												
Self-Test	0.490	0.396	0.584	1.632	1.486	1.792	0.538	0.434	0.641	1.712	1.543	1.899
Post	0.322	0.227	0.416	1.380	1.255	1.516	0.322	0.218	0.425	1.380	1.244	1.530
Pharmacy	0.199	0.115	0.283	1.220	1.122	1.327	0.104	0.011	0.196	1.109	1.011	1.217
Education/ Work	-0.162	-0.247	-0.077	0.851	0.781	0.926	-0.251	-0.344	-0.157	0.778	0.709	0.854
GP Practice	-0.006	-0.093	0.081	0.994	0.911	1.085	0.063	-0.032	0.159	1.066	0.969	1.172
Sexual Health Clinic	0.000	-	-	1.000	-	-	0.000	-	-	1.000	-	-
**Time to Result**												
30 Mins	0.548	0.471	0.625	1.730	1.602	1.867	0.640	0.556	0.724	1.896	1.743	2.062
2 Hours	0.271	0.190	0.352	1.311	1.209	1.422	0.416	0.327	0.505	1.516	1.387	1.657
7 Days	0.000	-	-	1.000	-	-	0.000	-	-	1.000	-	-
14 Days	-0.148	-0.220	-0.077	0.862	0.803	0.926	-0.149	-0.227	-0.071	0.862	0.797	0.932
**Accuracy**												
2 in 100 False Negative	1.186	1.137	1.236	3.275	3.116	3.443	1.196	1.141	1.250	3.305	3.129	3.492
5 in 100 False Negative	0.000	-	-	1.000	-	-	0.000	-	-	1.000	-	-
**Consultation Method**												
Online Consultation	0.145	0.071	0.219	1.156	1.074	1.245	0.251	0.169	0.332	1.285	1.185	1.394
Pharmacy Consultation	0.108	0.035	0.182	1.115	1.036	1.199	0.203	0.123	0.284	1.225	1.130	1.328
GP Consultation	0.150	0.077	0.223	1.162	1.080	1.250	0.173	0.092	0.253	1.189	1.097	1.288
Sexual Health Clinic Consultation	0.000	-	-	1.000	-	-	0.000	-	-	1.000	-	-
**Access to Healthcare Professional**												
Phone	-0.059	-0.130	0.011	0.942	0.878	1.011	-0.077	-0.155	0.000	0.926	0.856	1.000
Instant Messenger	0.025	-0.045	0.095	1.025	0.956	1.099	-0.009	-0.086	0.068	0.991	0.918	1.071
Email	0.066	-0.003	0.136	1.069	0.997	1.145	0.001	-0.076	0.077	1.001	0.927	1.080
Face to Face	0.000	-	-	1.000	-	-	0.000	-	-	1.000	-	-
**How you get Antibiotics**												
Post to Home	-0.044	-0.118	0.030	0.957	0.889	1.030	0.053	-0.028	0.134	1.055	0.973	1.144
Post to Collection Point	-0.032	-0.104	0.040	0.968	0.901	1.041	0.070	-0.009	0.149	1.072	0.991	1.160
Collect from Pharmacy	0.067	-0.009	0.143	1.070	0.991	1.154	0.055	-0.029	0.139	1.056	0.971	1.149
Collect from Sexual Health Clinic	0.000	-	-	1.000	-	-	0.000	-	-	1.000	-	-

Table 3.5 - Previous STI Testing Subgroup Analysis Coefficients and Odds Ratios, including Respective 95% Confidence Interval

	**Previous Test n=393**						**No Previous Test *n=790***					
	**Coefficient**	**Lower 95% CI**	**Upper 95% CI**	**Odds Ratio**	**Lower 95% CI**	**Upper 95% CI**	**Coefficient**	**Lower 95% CI**	**Upper 95% CI**	**Odds Ratio**	**Lower 95% CI**	**Upper 95% CI**
**How you Test**												
Self-Test	0.422	0.305	0.539	1.524	1.356	1.714	0.518	0.434	0.601	1.678	1.544	1.824
Post	0.164	0.047	0.280	1.178	1.048	1.323	0.382	0.298	0.466	1.465	1.347	1.594
Pharmacy	-0.056	-0.160	0.048	0.945	0.852	1.049	0.245	0.170	0.320	1.277	1.185	1.377
Education/ Work	-0.273	-0.377	-0.168	0.761	0.686	0.845	-0.150	-0.225	-0.074	0.861	0.798	0.928
GP Practice	-0.122	-0.229	-0.014	0.885	0.795	0.986	0.097	0.019	0.174	1.101	1.019	1.190
Sexual Health Clinic	0.000	-	-	1.000	-	-	0.000	-	-	1.000	-	-
**Time to Result**												
30 Mins	0.592	0.498	0.687	1.808	1.645	1.988	0.586	0.518	0.654	1.797	1.679	1.923
2 Hours	0.305	0.205	0.406	1.357	1.227	1.500	0.353	0.281	0.425	1.423	1.325	1.529
7 Days	0.000	-	-	1.000	-	-	0.000	-	-	1.000	-	-
14 Days	-0.125	-0.213	-0.037	0.882	0.808	0.963	-0.156	-0.219	-0.093	0.856	0.803	0.912
**Accuracy**												
2 in 100 False Negative	1.099	1.037	1.160	3.000	2.820	3.191	1.248	1.203	1.292	3.482	3.331	3.640
5 in 100 False Negative	0.000	-	-	1.000	-	-	0.000	-	-	1.000	-	-
**Consultation Method**												
Online Consultation	0.161	0.069	0.252	1.174	1.071	1.287	0.205	0.140	0.271	1.228	1.150	1.311
Pharmacy Consultation	0.094	0.003	0.184	1.098	1.003	1.203	0.168	0.103	0.233	1.183	1.108	1.262
GP Consultation	0.106	0.015	0.197	1.112	1.015	1.217	0.195	0.130	0.260	1.215	1.139	1.297
Sexual Health Clinic Consultation	0.000	-	-	1.000	-	-	0.000	-	-	1.000	-	-
**Access to Healthcare Professional**												
Phone	-0.054	-0.142	0.034	0.947	0.868	1.034	-0.048	-0.110	0.015	0.953	0.895	1.015
Instant Messenger	0.044	-0.042	0.131	1.045	0.958	1.140	0.025	-0.037	0.087	1.025	0.963	1.091
Email	0.048	-0.038	0.134	1.049	0.962	1.144	0.053	-0.009	0.114	1.054	0.991	1.121
Face to Face	0.000	-	-	1.000	-	-	0.000	-	-	1.000	-	-
**How you get Antibiotics**												
Post to Home	0.011	-0.081	0.102	1.011	0.922	1.107	0.020	-0.046	0.085	1.020	0.955	1.089
Post to Collection Point	0.034	-0.056	0.123	1.034	0.946	1.131	0.034	-0.029	0.098	1.035	0.971	1.103
Collect from Pharmacy	0.083	-0.012	0.177	1.086	0.988	1.194	0.075	0.008	0.143	1.078	1.008	1.153
Collect from Sexual Health Clinic	0.000	-	-	1.000	-	-	0.000	-	-	1.000	-	-
