# Supplementary material for: Young people’s preferences for the use of emerging technologies for asymptomatic regular chlamydia testing and management: a discrete choice experiment in England
Source: BMJ Open. 2019 Jan 29;9(1):e023663. doi: 10.1136/bmjopen-2018-023663 (PMC6352830; doi:10.1136/bmjopen-2018-023663)
Supplement: Author's manuscript [file bmjopen-2018-023663.draft_revisions.pdf]

BMJ Open is committed to open peer review. As part of this commitment we make the peer review history of every article we publish publicly available.

When an article is published we post the peer reviewers' comments and the authors' responses online. We also post the versions of the paper that were used during peer review. These are the versions that the peer review comments apply to.

The versions of the paper that follow are the versions that were submitted during the peer review process. They are not the versions of record or the final published versions. They should not be cited or distributed as the published version of this manuscript.

BMJ Open is an open access journal and the full, final, typeset and author-corrected version of record of the manuscript is available on our site with no access controls, subscription charges or pay-per-view fees (http://bmjopen.bmj.com).

If you have any questions on BMJ Open's open peer review process please email editorial.bmjopen@bmj.com

BMJ Open

Young people's preferences for the use of emerging technologies for asymptomatic regular chlamydia testing and management: A discrete choice experiment in England

Journal:	*BMJ Open*
Manuscript ID	bmjopen-2018-023663
Article Type:	Research
Date Submitted by the Author:	14-May-2018
Complete List of Authors:	Eaton, Sue; University of Warwick, Warwick Medical School<br>Biggerstaff, Deborah; University of Warwick Warwick Medical School<br>Petrou, Stavros; University of Warwick, Warwick Medical School<br>Osipenko, Leeza; National Institute for Health and Care Excellence, Scientific Advice<br>Gibbs, Jo; University College London, Research Department of Infection and Population Health<br>Estcourt, Claudia; Glasgow Caledonian University,<br>Sadiq, S. Tariq; St George's University of London, Institute for Infection and Immunity Research Institute<br>Szczepura, Ala; Coventry University, Enterprise & Innovation Group; University of Warwick Warwick Medical School
Keywords:	sexually transmitted infections, chlamydia trachomatis, patient preferences, discrete choice experiment, online clinical care pathway, digital health

SCHOLARONE™
Manuscripts

Title: Young people's preferences for the use of emerging technologies for asymptomatic regular chlamydia testing and management: A discrete choice experiment in England

Authors:
Dr Sue Eaton, Warwick Medical School, University of Warwick, Coventry, UK.
Dr Deborah Biggerstaff, Warwick Medical School, University of Warwick, Coventry, UK.
Prof Stavros Petrou, Warwick Medical School, University of Warwick, Coventry, UK.
Dr Leeza Osipenko, National Institute for Health and Care Excellence, Scientific Advice, London.
Dr Jo Gibbs, Research Department of Infection & Population Health, University College London, London, UK.
Prof Claudia S Estcourt, Glasgow Caledonian University, Glasgow, UK.
Dr S Tariq Sadiq, Applied Diagnostic Research and Evaluation Unit, Institute for Infection and Immunity, St George's, University of London, London, UK.
Prof Ala Szczepura, Enterprise & Innovation Group, Coventry University, UK.

Corresponding author
Prof Ala Szczepura, BA DPhil (Oxon)
Enterprise & Innovation Group
Coventry University
Coventry CV1 2TT
UK
Email: ala.szczepura@coventry.ac.uk
Mob: +44 (0)7557 425 463

Word Count *(excluding abstract, references, figures and tables)*: **3,987**

Key words: sexually transmitted infections, chlamydia trachomatis, patient preferences, discrete choice experiment, online clinical care pathway, digital health

For peer review only - http://bmjopen.bmj.com/site/about/guidelines.xhtml

ABSTRACT:

Objective: To undertake a comprehensive assessment of the strength of preferences among young people for attributes of emerging technologies for testing and treatment of asymptomatic chlamydia.

Design: Discrete choice experiment (DCE) with sequential mixed methods design. A staged approach to selection of attributes/ levels included two literature reviews, focus groups with young people aged 16-24 years (n = 21), experts' review (n=13), and narrative synthesis. Cognitive testing was undertaken to pilot and adapt the initial questionnaire. Online national panel was used for final DCE survey to maximise generalisability. Analysis of questionnaire responses used multinomial logit models and included validity checks.

Setting: England.

Participants: 1,230 young people aged 16-24 from a national online panel (completion rate 73%).

Outcome Measures: Odds Ratios (ORs) for service attributes in relation to reference levels.

Results: The strongest attribute influencing preferences was chlamydia test accuracy (OR 3.24, 95% CI 3.13–3.36), followed by time to result (OR 1.81, 95% CI 1.71–1.91). Respondents showed a preference for remote chlamydia testing options (self-testing, self-sampling and postal testing) over attendance at a testing location. For accessing treatment, a general preference for online (OR 1.21, 95% CI 1.15–1.28) versus traditional GP (OR 1.18, 95% CI 1.12–1.24) or pharmacy (OR 1.15, 95% CI 1.10–1.22)) services over clinic services was observed. For accessing a healthcare professional and receipt of antibiotics, there was little difference in preferences between attribute levels.

Conclusions:
Both test accuracy and very short intervals between testing and results were important factors for young people when deciding whether to undergo a routine test for asymptomatic chlamydia, with test accuracy being more important. These findings should assist technology developers, policy makers, commissioners and service providers to optimise technology adoption in service redesign, although use of an online panel may limit generalisability of findings to other populations.

For peer review only - http://bmjopen.bmj.com/site/about/guidelines.xhtml

Strengths and Limitations of the Study:

- To our knowledge, this is the first DCE study to examine the preferences of a large, nationally representative sample of young people for new approaches designed to improve screening for and treatment of chlamydia.
- Robust sequential methods were used to select final DCE attributes and levels, including two literature reviews, focus groups with young people, and review by expert groups.
- An online panel enabled access to the target population for screening, including young people with no personal experience of chlamydia testing and treatment.
- A limitation of this work is that the literature reviews inevitably identified more potential attributes than could be included in the DCE.
- The use of an online panel may limit generalisability of findings to other populations and over-represent the acceptability of online care.

For peer review only

For peer review only - http://bmjopen.bmj.com/site/about/guidelines.xhtml

INTRODUCTION:

Chlamydia is the most commonly reported sexually transmitted infection (STI) in England, with young people aged 15-24 accounting for 63% of diagnoses in 2016.[1] The estimated annual cost of chlamydia treatment in 2015 was £249.8 million.[2] Undetected infections and re-infections can lead to significant adverse health consequences, such as pelvic inflammatory disease, ectopic pregnancy and infertility, which have an impact on National Health Service (NHS) costs and health-related quality of life.[3] A National Chlamydia Screening Programme (NCSP) was therefore introduced in England for 16-24 year olds in 2003.[4] However, despite the introduction of this national programme, and the fact that uncomplicated infection is easy to treat with oral antibiotics, uptake of screening for chlamydia remains low.[5] There has been a decline over the last four years in the number of local authorities achieving the public health outcomes framework indicator of 2,300 diagnoses per 100,000 population.[1] A number of barriers have been identified for young people accessing STI testing and treatment services, including tangible service attributes, such as location of service, and personal factors, such as the stigma associated with attendance, embarrassment, fear of being recognised and privacy concerns.[6-8]

A range of options are currently available for young people to access chlamydia testing and treatment services. For testing, options include attending a genito-urinary medicine (GUM) clinic, testing via primary care, e.g. pharmacies/general practitioners (GPs), or internet testing services where a number of websites offer free self-sampling kits online with samples sent to laboratories for analysis and results communicated directly to patients. For individuals testing positive, options for accessing antibiotic treatment include attending a GUM/sexual health clinic, GP practice or pharmacy. Even though a national screening programme exists in England, large geographical variations exist in testing coverage, particularly in non-specialist sexual health services and in the proportion of positive cases treated.[9]

For many health services, digital technology is now widely regarded by policy makers as one approach to improve access and reduce costs.[10] For chlamydia testing and treatment, a range of digital options are already available, and more technologies are likely to enter the market within the next 3-5 years.[11] These include point-of-care tests[12], online postal self-sampling, eSexual health clinics[13], apps and non-face-to-face consultation methods[14 15], and self-tests networked through mobile phones[16]. Such innovations will provide the opportunity to redesign current services and potentially improve chlamydia testing and treatment uptake. However, any service redesign should be based on a sound understanding of the preferences of service-users and, amongst these, particularly young people for changing service attributes.

Research specifically measuring young people's preferences for chlamydia testing and treatment services is lacking. For STI testing services more broadly, relatively few studies have used a discrete choice experiment (DCE) design to assess preferences in UK populations.[17-20] In comparison with other preference elicitation methods, a DCE can quantify the relative importance of a range of attributes that characterise a new or existing product and/or service, identifying which attributes people may be willing to trade with the view to maximising their utility.[21]

For peer review only - http://bmjopen.bmj.com/site/about/guidelines.xhtml

A DCE requires respondents to choose between competing scenarios, e.g. service options, described in terms of their attributes (e.g. time to test result) and levels (e.g. 30min, 2hrs, 7 or 14 days*)* and compare these against at least one alternative scenario. Such studies are very useful because they allow a direct assessment of relative preferences for different existing and new service configurations or treatment approaches.[22]

The aim of the present study was to undertake a comprehensive assessment of young people's preferences for emerging technologies for chlamydia testing and treatment services in the context of a "check-up test" when remote care could be medically appropriate. To our knowledge, no previous study has disentangled strength of preference for attributes associated with new and emerging options for testing (such as self-testing) or treatment (such as online prescription) of STIs. Because STI services are "open access" and not subject to gatekeeping by referral from a clinician, they are directly dependent on individuals' preferences. Early insight into the attributes that influence whether individuals would use a new testing or treatment pathway should therefore be helpful in informing product development, pathway redesign and the assumptions used in economic models for future chlamydia testing and management.[23 24]

METHODS:

This study was conducted using an exploratory sequential mixed methods design, adopting recommended stages for undertaking a DCE[25], as shown in Figure 1. In selecting methods to design the questionnaire and conduct the DCE, reference was made to the International Society for Pharmacoeconomics and Outcomes Research good practice checklist for conjoint analysis.[26]

Patient and public involvement: Young people (with and without experience of STI services) participated in focus groups to inform priorities included in the DCE; in cognitive testing to finalise questionnaire design; and in questionnaire completion to identify preferences, as described in the Acknowledgements.

Selection of Attributes and Levels

The attributes and associated levels were determined using a four-stage approach.[27]

Literature Reviews

For peer review only - http://bmjopen.bmj.com/site/about/guidelines.xhtml

Two literature reviews were undertaken to produce a comprehensive list of potential themes and factors that might influence young people's choices. These included: (i) a systematic review of the use of stated preference studies for STI testing and treatment (PROSPERO Reg: CRD42014014862); and (ii) a scoping review of other research exploring preferences and acceptability of STI testing and treatment services.

Focus Groups

Focus groups were run with young people aged 16-24 years (3-7 per group; total n=21) to identify which themes and factors young people consider important when choosing to test for STIs. Convenience sampling was used to identify participants. Focus group topic guides incorporated typical vignettes of situations individuals might encounter. Sessions were recorded, transcribed and thematic analysis was performed to produce a list of potential attributes and levels.[28]

Expert Groups

Four expert groups were convened (n=13), including a range of professionals with expert knowledge of the service and technology context. Expert groups were asked to review the focus group findings in terms of clinical feasibility and practicality.[25]

Narrative Synthesis

Narrative synthesis[29] enabled outputs from the three prior stages to be synthesised for each potential attribute. This approach was adopted as it offered a clear approach to synthesis based on the following stages: (i) identification of a checklist of properties against which attributes can be considered; (ii) tabulation against the checklist; and (iii) conceptual mapping and triangulation against the checklist. The final synthesis highlighted a tension between young people's desire to be tested for a wide range of STIs irrespective of risk, versus clinical guidelines for selective testing of STIs based on population group prevalence and risk. The range of STIs presented in the DCE was consequently limited to Chlamydia. Because focus group findings indicated difficulty in understanding several dimensions of test performance, test accuracy was expressed in terms of the likelihood of a false negative result.

Questionnaire Design and Piloting

A generic pairwise choice with opt out question was selected for the questionnaire design. Respondents were presented with a series of choice sets for which there were 3 responses: 'option A', 'option B' or 'I would not test'. A sample choice set is illustrated in Figure 2. The questionnaire adopted a main effects design using full profiles (all attributes included in the study). Whilst some DCEs do include an attribute on economic costs, DCEs exploring preferences for STI testing services in England have generally excluded cost since the NHS provides treatment 'free at the point of delivery'[19 20] A cost attribute was therefore not included.

For peer review only - http://bmjopen.bmj.com/site/about/guidelines.xhtml

The questionnaire was developed using SAS 9.4 software to ensure that the design was D-efficient.[26] A full factorial design was ruled out in favour of a fractional factorial design because a full factorial design would have contained 3,072 possible alternatives, which would have been unmanageable in practice for individuals to complete or for a blocked questionnaire format to handle. The smallest 100% efficient design that could be created included 48 choice sets. These were blocked (halved) into two questionnaires each with 24 choice sets using SAS JMP Pro 9.2.0. Choice set 1 was repeated as choice set 25 in each questionnaire to provide an internal validity check.

Three rounds of cognitive testing (n=9) were undertaken to check respondents' comprehension of information when making choices. Some modifications were made to levels where reasons for choice selection demonstrated that one level (e.g. 8 in 100 false negative) dominated the reason for selection. Cognitive testing also identified that implausible combinations (e.g. a postal test providing a result in two hours) impacted on completion of the task, whilst unlikely (but feasible) combinations did not. Only implausible combinations were therefore excluded from the choice sets. The D-Efficiency of the design[26] calculated by SAS JMP Pro 9.2.0, prior to the removal of implausible combinations, was 98.06. The final combination selected for the questionnaire was the one which created no duplicate choices and which provided an equal balance of the number of choice sets containing overlap between questionnaires.

Respondents were asked to provide socio-demographic information, including their age, gender, ethnicity, region of residence, sexual preference, relationship status and whether they had previously been tested for an STI. An introduction to the DCE questionnaire provided background information and an explanation of the attributes and levels. The introduction was included in the cognitive testing rounds to check comprehension within the target age range. As a result, the text was modified and diagrams added to illustrate the chlamydia testing pathways. The final phase of testing demonstrated that the number of choice sets was acceptable to respondents. The six attributes and 24 levels selected for inclusion in the final DCE questionnaire are shown in table 1. The detailed information used to explain the attributes and levels is included in Supplementary Information File 1. The use of an online panel also provided completion time data to support the internal validity checks, and enabled an accurate record of time taken to complete the survey.

Participants and Recruitment Procedure

Participants were drawn from the general population rather than from health care settings, thereby providing access to young people aged 16-24 years who had not previously been tested for an STI. An online national panel (YouthSight)[30] was used to maximise geographical reach and generalisability. Ex-ante sub-group analysis was planned by three age bands (16-18, 19-21, 22-24) and by gender (male, female). For this, a sample size of 1,200 was required, 600 per questionnaire.[31] Consent was obtained online prior to questionnaire completion. Participants were offered a small reimbursement of one point (equivalent to £1) for completion of a survey of up to 20 minutes in length. Points could be exchanged for shopping vouchers.

For peer review only - http://bmjopen.bmj.com/site/about/guidelines.xhtml

Statistical Analyses

The multinomial logit (MNL) model developed by McFadden was used for analysing responses; this is recognised as the convention for three-response choice set studies ('option A', 'option B' or 'I would not test').[25 32] Analysis utilised STATA13 SE with the method and code outlined by Ryan *et al*[22] Analyses presented odds ratios (ORs), 95% confidence interval (CIs) and coefficients for each attribute level. Variables within the model were all treated as categorical variables for the analysis. Attribute levels were specified using dummy coding, the preferred form of coding where ORs are to be calculated. Within the model, the levels that were dropped to form the reference levels reflected those aligned to a 'typical' sexual health clinic pathway (summarised in table 2). To test the internal validity of questionnaire responses, analyses compared full results against: (i) removal of responses where participants did not answer the repeated choice set consistently; (ii) removal of any respondents who took less than the minimum time (five minutes) observed in cognitive testing to complete the questionnaire; and (iii) removal of responses containing the opt out question data. Further tests for internal consistency and rationality were not included, since excluding responses on this basis may be viewed as an inappropriate imposition of rationality.[33]

Demographic characteristics (gender, age and ethnicity) of respondents were compared with national Census data.[34] The influence of patient level characteristics (age, sex and STI testing history (yes/no)) on the likelihood of not choosing to test was examined. In addition to *ex-ante* planned sub-group analyses, if sufficient responses were received analysis was also planned to compare: (i) respondents who had, or had not, previously tested for an STI, and (ii) those who indicated their relationship status as 'single' versus those in a sexual relationship with one person.

Trade-off between accuracy and time to result was examined by considering the probability of uptake for tests with characteristics at the opposite ends of the spectrum i.e. 'lower accuracy (5% false negatives), faster time to result (30 min)' and 'higher accuracy (2% false negatives), longer time to result (14 days)'.

RESULTS:

In total, 1,230 fully completed questionnaires were received, whilst a further 490 people commenced the questionnaire but did not complete it, providing a completion rate of 73%. Time to complete the 25 choice sets ranged from one minute 19 seconds to 30 minutes 19 seconds with a median time to completion of seven minutes 51 seconds. Demographic characteristics of respondents are summarised in the Supplementary Information File 2. Comparing the DCE sample to the characteristics of 16-24 year olds in England indicates that gender, age and ethnicity are broadly in line with national population demographics, with a slight under-representation of the 16-18 year age group. In terms of the

For peer review only - http://bmjopen.bmj.com/site/about/guidelines.xhtml

geographical residence of participants, the sample was broadly in line with the geographical distribution of 16-24 year olds identified in the ONS Mid-Year Population Estimates 2015[35], with the exception of a lower proportion of respondents from the East of England.

Internal validity checks showed that, in comparison to the full dataset, all checks yielded very similar ORs with no change in the order of the strength of preference for a level within each attribute. Results of the internal validity checks are included in the Supplementary Information File 2 (Tables 2.1 – 2.4).

The ORs for the full dataset and subgroups analysed are presented in Table 3. Additional DCE results, including the 95% CIs for all sub-group analysis are presented in Supplementary Information File 2.

Looking across all attributes and levels, participants expressed the strongest preference for accuracy of test result (OR 3.242). Table 3 shows differences in the strength of preference between males and females (OR 2.950 and 3.570, respectively), and between those who had previously tested or not tested (OR 3.000 and 3.482, respectively). Time to result was the attribute showing the next strongest preference across all subgroups. These results are consistent with the logical expectation that people will prefer higher accuracy and a shorter waiting time. Looking specifically at the trade-off between accuracy and time to result, Table 4 indicates that participants are willing to wait noticeably longer in order to have a test result with a lower chance of a false negative result.

When considering how to test, all subgroups demonstrated a preference for self-testing (OR 1.618). Testing via an outreach service in an educational/ work setting was found to be the least preferred option (OR 0.821). Results showed a consistent strength of preference for those options that do not involve direct contact with healthcare professionals (self-testing and postal self-sampling), compared with those that do. For consultation and treatment methods following a positive test result, there was a preference for non-sexual health clinic pathways with online consultation (OR 1.212), treatment via general practice (OR 1.183) and treatment via pharmacy (OR 1.158) preferred in the full dataset. At subgroup level (age, gender, previous testing history and relationship status), more variation was found in the preference order for this attribute, with the exception of the sexual health clinic which was consistently the least preferred option across all subgroups (see table 3 and supplementary information file 2). The full dataset shows that, for accessing a healthcare professional, there was no statistically significant preference for instant messenger or email access compared with accessing the professional face-to-face. Telephone access to a healthcare professional was the least preferred access option (OR 0.949), and the only statistically significant result when compared with face-to-face access (the referent). There was, similarly, very little difference in preferences for how young people might access antibiotics.

For peer review only - http://bmjopen.bmj.com/site/about/guidelines.xhtml

DISCUSSION:

Our findings indicate that, based on the levels included in the DCE questionnaire, participants were willing to wait considerably longer in order to have a test result with a lower chance of a false negative result. The conclusion for new test developers is that time to result is less important than accuracy, and that test users are unlikely to prefer a point-of-care test or self-test with lower accuracy than the tests currently available to them. There was a strong preference for remote access to testing, consultation, and antibiotic prescriptions, although for accessing a health professional there was no preference between online and face-to-face methods. This suggests a remote online pathway is acceptable to young people, *as long as* test performance remains equivalent.

In the various hypothetical situations presented, respondents showed a preference for chlamydia self-testing, self-sampling and postal testing over attendance at a testing location. For accessing treatment, a general preference was exhibited for online versus traditional GP or pharmacy services over clinic services. For receipt of antibiotics, there was little difference in preferences. We were also able to identify which attributes people may be willing to trade to maximise their utility. Looking at the trade-off between accuracy and time to result, we found that young people are willing to wait noticeably longer in order to have a test result with a lower chance of a false negative result, reinforcing the need for test equivalence.

The strengths of this DCE study include, firstly, the robust methods employed to select attributes and levels, which aligned with the recommendations of Coast *et al*, ensuring attributes are "manipulable in policy".[36] Secondly, the fact that participants were drawn from the general population targeted by the National Chlamydia Screening Programme, rather than from healthcare settings. This enabled us to access a demographically representative national sample of young people, including those who had not had previous contact with STI services. Finally, the use of an online panel enabled large scale data to be collected at a reasonable cost and allowed validity checks that would not otherwise have been possible with written questionnaire responses. The large sample size also allowed comparison between several subgroups identifying any differences based on age, gender, previous testing history and relationship category.

However, the study does have a few limitations. The first relates to the selection of attributes and levels. Whilst the selection process employed was very rigorous, this cannot detract from the fact that further attributes were identified, which might impact on individuals' choices. To mitigate against this, where such an attribute was excluded, information was provided in the survey background section to minimise respondents forming their own views on the impact of this attribute on the pathway.

Use of an online panel also provided accurate records of the time taken to complete the survey, permitting additional validity checks, which would not otherwise have been possible with a written questionnaire. On the other hand, use of an online panel did exclude populations who

For peer review only - http://bmjopen.bmj.com/site/about/guidelines.xhtml

do not have access to the internet, thereby potentially over-representing the acceptability of online care. However, given the extremely high proportion (97%) of 15-24 year-olds accessing the internet daily via a mobile device[37] and owning a smartphone[38], it is evident that the vast majority of the target population can have access to online care pathways if they choose to do so. The question of whether these individuals have the degree of digital and health literacy needed for online self-sampling, testing and treatment was not explored in this study.

Identified barriers to accessing current sexual health services, such as embarrassment[6]; access and convenience[7]; stigma;[39] and privacy and anonymity concerns,[40 41] should be lower for an online service. Balanced against this, non-face-to-face access to testing and treatment services was valued less strongly than attributes such as test accuracy and time to result. In such cases a young person's preferences for remote versus face-to-face testing and treatment might be different.

Finally, it is difficult to compare the present results to other published DCEs because of key differences in focus. Miners *et al* (sample n=3,358)[19] and Llewellyn *et al* (sample n=233)[20] both only focussed on preferences within existing traditional service delivery models, and did not incorporate hypothetical future scenarios (e.g. point-of-care testing, self-testing or treatment via eHealth/mHealth solutions). Two other DCE studies, which did consider self-sampling at home for chlamydia screening[17 18], sample size 174 & 126 respectively, both described sending the sample to a laboratory for analysis rather than a self-test. One of these studies[18] did identify a stronger preference for attendance at a family planning clinic, rather than self-sampling, but since participants were recruited from the waiting room of such a clinic they cannot be considered representative of the population targeted by the NCSP. A fifth study, which examined preferences for point-of-care testing, only surveyed clinicians undertaking STI testing (sample n=218), thus excluding the population targeted for chlamydia testing.[42] The only stated preference study that has considered patients' preferences for STI self-testing focussed on HIV (sample n=365).[43] The authors reported results in line with our findings, with respondents exhibiting a preference for tests which are accurate, timely and private/ anonymous. However, this study was undertaken in 2002 prior to smartphones and at a time when self-testing for HIV was still under development. Importantly for screening tests, only one DCE study to date[20] has sought to include non-service users (i.e. populations with no experience of STI testing). The authors identified a preference for testing for all STIs, in settings with healthcare professionals with specialist knowledge present, and for receipt of negative as well as positive results. This study used a convenience sample of 233 students from two universities which was unlikely to be geographically or socio-economically representative. All other studies have drawn their DCE samples from people who were either current service users, or attendees for other, linked services. Use of such samples represents a significant short-coming when considering the introduction of new digital technologies for asymptomatic chlamydia testing and management. Sample size is also generally smaller than the present study.

For peer review only - http://bmjopen.bmj.com/site/about/guidelines.xhtml

A number of questions remain which our research does not address. Firstly, recognising that the range of STIs included is an important consideration for young people in choosing to test[19 20], further research is required to understand this better in the context of potential new pathways which incorporate other STIs (e.g. gonorrhoea). Secondly, the study highlights a number of methodological considerations where there is an absence of consensus that may warrant further exploration to improve consistency. These include the number of choice sets to include in a DCE and the use of repeated choice sets as an internal validity measure. Finally, given that cost was not included as an attribute in this study, it is not possible to provide an indication of willingness to pay, or the monetary benefits of potential service changes, from this DCE.[44]

The present study is, to our knowledge, the first to present large scale, quantitative evidence of young people's preferences for attributes of new pathways and emerging technologies in the testing and treatment of chlamydia (based on a demographically representative national sample). The DCE methodology applied also produces a measure of the relative strength of preference between different attributes and levels, and potential trade-offs. This can provide useful evidence to technology developers, policy makers, commissioners and service providers. In particular, it provides a first insight into preferences for technologies currently under development, and those which might be available for use in the near future, compared to the features of existing products and services. This can indicate how young people may respond to changes in pathways and to the introduction of new technologies.

Within the context of current UK sexual health policy and commissioning of sexual health services[11], this DCE provides supportive evidence for the policy direction of remote chlamydia testing and treatment. However, whilst young people overall expressed a stronger preference for attributes such as self-test, online consultation etc, a small proportion still preferred existing pathways. This suggests that, in order to maximise benefit, face-to-face services should continue to be available in addition to any online care service. This will ensure that services are inclusive and accessible irrespective of digital / health literacy, while recognising that people's needs and preferences may change depending on their personal circumstances.

Acknowledgements

We thank Dr Joshua Pink, National Institute for Health and Care Excellence (NICE), Manchester, UK for providing specialist advice on DCE design and analysis. We would also like to thank academics in the Studies in Adolescent Sexual Health (SASH) Group at Coventry University who provided advice on how to access the young people who participated in focus groups; and YouthSight for their expertise in converting the questionnaire into an online format. Finally, the authors wish to thank all the young people who took the time to participate in the focus groups, cognitive testing and complete a detailed questionnaire describing their preferences, and the experts who contributed their views in the expert groups.

For peer review only - http://bmjopen.bmj.com/site/about/guidelines.xhtml

Contributors:
SE conceived and undertook the study as part of her PhD research. AS was lead supervisor and takes responsibility for the integrity of the data and the accuracy of the data analysis. SP, LO and AS oversaw the DCE design and analysis; DB guided the qualitative research. CSE and JG provided clinical input into the development of the DCE materials and assisted with interpretation of findings. SE, AS, SP, LO and DB wrote the first draft of the article. STS (Principal Investigator), AS and CSE were applicants on the eSTI2 Consortium grant, wrote the initial protocol and obtained funding. All authors contributed to and agreed the final version of the manuscript.

Funding:
The Electronic Self-Testing Instruments for Sexually Transmitted Infection Control (eSTI^2) Consortium (which funded SE's studentship and also AS, JG, CSE and STS) was funded under the UKCRC Translational Infection Research (TIR) Initiative supported by the Medical Research Council (Grant Number G0901608) with contributions to the Grant from the Biotechnology and Biological Sciences Research Council, the National Institute for Health Research on behalf of the Department of Health, the Chief Scientist Office of the Scottish Government Health Directorates and the Wellcome Trust.

The funding body had no role in the design of the study, analyses, interpretation, or decision to submit the manuscript for publication.

Competing Interests:
None Declared

Ethics Approval
Ethical approval was granted by the Biomedical & Scientific Research Ethics Committee at the University of Warwick (ref: REGO-2015-1647).

Data Sharing Statement:
Requests for access to data should be addressed to the corresponding author

For peer review only - http://bmjopen.bmj.com/site/about/guidelines.xhtml

References

1. Public Health England. Sexually Transmitted Infections and Chlamydia Screening in England, 2016. Secondary Sexually Transmitted Infections and Chlamydia Screening in England, 2016 2017. https://assets.publishing.service.gov.uk/government/uploads/system/uploads/attachment_data/file/617025/Health_Protection_Report_STIs_NCSP_2017.pdf

2. Development Economics. Unprotected Nation - The Financial and Economic Impacts of Restricted Contraceptive and Sexual Health Services. Secondary Unprotected Nation - The Financial and Economic Impacts of Restricted Contraceptive and Sexual Health Services 2013. https://www.fpa.org.uk/sites/default/files/unprotected-nation-sexual-health-full-report.pdf.
3. Public Health England. Opportunistic Chlamydia Screening of Young Adults in England: An Evidence Summary Secondary Opportunistic Chlamydia Screening of Young Adults in England: An Evidence Summary 2014. https://assets.publishing.service.gov.uk/government/uploads/system/uploads/attachment_data/file/497371/Opportunistic_Chlamydia_Screening_Evidence_Summary_April_2014.pdf.
4. Public Health England. Guidance: NCSP: programme overview. Secondary Guidance: NCSP: programme overview 2013. https://www.gov.uk/government/publications/ncsp-programme-overview/ncsp-programme-overview.
5. Public Health England. Health protection report, volume 10, issue 22. Secondary Health protection report, volume 10, issue 22 2016. https://www.gov.uk/government/publications/health-protection-report-volume-10-2016/hpr-volume-10-issue-22-news-8-july
6. Booth A R, Harris P R, Goyder E, et al. Beliefs about chlamydia testing amongst young people living in relatively deprived areas. J Public Helath (Oxf) 2013;**35**:213-22.
7. Friedman AL, Bloodgood B. Exploring the feasibility of alternative STD-testing venues and results delivery channels for a national screening campaign. Health Promot Pract 2013;**14**(1):96-104.
8. Balfe M, Brugha R. What prompts young adults in Ireland to attend health services for STI testing? BMC Public Health 2009;**9**:311.
9. NCSP. NCSP Scorecard 2011-12. Secondary NCSP Scorecard 2011-12 2012. http://www.chlamydiascreening.nhs.uk/ps/data.asp.
10. National Information Board & Department of Health. Personalised Health and Care 2020 - Using Data and Technology to Transform Outcomes for Patients and Citizens, A Framework for Action. Secondary Personalised Health and Care 2020 - Using Data and Technology to Transform Outcomes for Patients and Citizens, A Framework for Action 2014. https://www.gov.uk/government/uploads/system/uploads/attachment_data/file/384650/NIB_Report.pdf.
11. Eaton S OL, Petrou S, Biggerstaff D, Szczepura A,. National Telemedicine and Telehealth Policy Context in England and Introduction of Digital Innovations for Detection and Treatment of Sexually Transmitted Infections. SM J Clin Med 2017;**3**(1).

For peer review only - http://bmjopen.bmj.com/site/about/guidelines.xhtml

12. Harding-Esch EM, Nori AV, Hegazi A, et al. Impact of deploying multiple point-of-care tests with a 'sample first' approach on a sexual health clinical care pathway. A service evaluation. Sex Transm Infect 2017;**93**(6):424-29.
13. Estcourt C GJ, Sutcliffe L, Gkatzidou V, Tickle L, Hone K, Aicken C, Lowndes C, Harding-Esch E, Sue Eaton S, Oakeshott P, Szczepura A, Ashcroft R, Copas A, Nettleship A, Sadiq SJ, Sonnenberg P,. The eSexual Health Clinic System for Sexually Transmitted Infection Management, Prevention and Control: Exploratory studies demonstrating safety, feasibility and public health utility. The Lancet Public Health 2017.
14. Lloyds Pharmacy. Online Doctor. Secondary Online Doctor 2018. https://onlinedoctor.lloydspharmacy.com.
15. City Health Care Partnership CIC. Sexual & Reproductive Healthcare Services. Secondary Sexual & Reproductive Healthcare Services 2018. www.sexualhealthvirtualclinic.co.uk.
16. UK Research and Innovation. Enabling and translating advances in diagnostic and communication technologies to reduce the burden of STIs. Secondary Enabling and translating advances in diagnostic and communication technologies to reduce the burden of STIs 2018. http://gtr.ukri.org/projects?ref=G0901608.
17. Ryan M, Watson V. Comparing welfare estimates from payment card contingent valuation and discrete choice experiments. Health Econ 2009;**18**(4):389-401.
18. Watson V, Ryan M, Watson E. Valuing experience factors in the provision of Chlamydia screening: an application to women attending the family planning clinic. Value Health 2009;**12**(4):621-3.
19. Miners A, Llewellyn C, Pollard A, et al. Assessing user preferences for sexually transmitted infection testing services: a discrete choice experiment. Sex Transm Infect 2012;**88**(7):510-6.
20. Llewellyn CD, Sakal C, Lagarde M, et al. Testing for sexually transmitted infections among students: a discrete choice experiment of service preferences. BMJ open 2013;**3**(10):e003240.
21. Lancsar E, Louviere J. Conducting discrete choice experiments to inform healthcare decision making: a user's guide. Pharmacoeconomics 2008;**26**(8):661-77.
22. Ryan M, Watson V, Krucien N, et al. Using Discrete Choice Experiments in Health Economics: Theoretical and Practical Issues. University of Aberdeen, UK: Health Economics Research Unit, 2014.
23. Hall J, Viney R, Haas M, et al. Using stated preference discrete choice modeling to evaluate health care programs. Journal of Business Research 2004;**57**:1026-32.
24. Ryan M. Discrete choice experiments in health care. BMJ 2004;**328**(7436):360-1.
25. Ryan M, Gerard K, Amaya-Amaya ME. *Using Discrete Choice Experiments to Value Health and Health Care.* Dordrecht, The Netherlands: Springer, 2008.

For peer review only - http://bmjopen.bmj.com/site/about/guidelines.xhtml

26. Bridges JF, Hauber AB, Marshall D, et al. Conjoint analysis applications in health--a checklist: a report of the ISPOR Good Research Practices for Conjoint Analysis Task Force. Value Health 2011;**14**(4):403-13.
27. Eaton S. Integrating Novel Digital Technology for the Testing & Treatment of Chlamydia into Mainstream Sexual Health Services in England. University of Warwick, 2017.
28. Silverman D. *Interpreting Qualitative Data, London, Sage Publications Ltd.* London: Sage Publications Ltd., 2014.
29. Pope C, Mays N, J. P. *Synthesising Qualitative and Quantitative Health Evidence A Guide to Methods*. Maidenhead, England: Open University Press, 2007.
30. Youthsight. YouthSight Panel Services. Secondary YouthSight Panel Services 2018. http://www.youthsight.com/expertise/panel-services/.
31. Orme B. *Getting Started with Conjoint Analysis: Strategies for Product Design and Pricing Research, Madison, Wisconsin, Research Publishers LLC.* Madison, USA: Research Publishers LLC, 2010.
32. Louviere J, Hensher DA, Swait JD. *Stated Choice Methods Analysis and Application.* Cambridge, England.: Cambridge University Press, 2003.
33. Clark MD, Determann D, Petrou S, et al. Discrete choice experiments in health economics: a review of the literature. Pharmacoeconomics 2014;**32**(9):883-902.
34. Office for National Statistics. 2011 Census, Key Statistics for Local Authorities in England and Wales . Secondary 2011 Census, Key Statistics for Local Authorities in England and Wales . 2012. http://webarchive.nationalarchives.gov.uk/20160107112030/http://www.ons.gov.uk/ons/publications/re-reference-tables.html?edition=tcm%3A77-286262.
35. Office for National Statistics. Population Estimates for UK, England and Wales, Scotland and Northern Ireland: mid-2015. . Secondary Population Estimates for UK, England and Wales, Scotland and Northern Ireland: mid-2015. 2016. https://www.ons.gov.uk/peoplepopulationandcommunity/populationandmigration/populationestimates/bulletins/annualmidyearpopulationestimates/mid2015.
36. Coast J, Al-Janabi H, Sutton EJ, et al. Using qualitative methods for attribute development for discrete choice experiments: issues and recommendations. Health Econ 2012;**21**(6):730-41.
37. Office for National Statistics. Internet access – households and individuals: 2016. Secondary Internet access – households and individuals: 2016. 2016. https://www.ons.gov.uk/peoplepopulationandcommunity/householdcharacteristics/homeinternetandsocialmediausage/bulletins/internetaccesshouseholdsandindividuals/2016.
38. Ipsos Connect. Technology Tracker. Quarterly Release: Q2 2016. Secondary Technology Tracker. Quarterly Release: Q2 2016. 2016. https://www.ipsos.com/sites/default/files/2016-10/Ipsos_Tech_Tracker_Q2_2016.pdf.

For peer review only - http://bmjopen.bmj.com/site/about/guidelines.xhtml

39. Balfe M, R. B. What concerns do young adults in Ireland have about attending health services for STD testing? Deviant Behav 2011;**32**:320-50.
40. Baraitser P, Brown KC, Gleisner Z, et al. 'Do it yourself' sexual health care: the user experience. Sex Health 2011;**8**(1):23-9.
41. Lorimer K, McDaid L. Young men's views toward the barriers and facilitators of Internet-based Chlamydia trachomatis screening: qualitative study. Journal of medical Internet research 2013;**15**(12):e265.
42. Hsieh YH, Gaydos CA, Hogan MT, et al. What qualities are most important to making a point of care test desirable for clinicians and others offering sexually transmitted infection testing? PloS one 2011;**6**(4):e19263.
43. Phillips KA, Maddala T, Johnson FR. Measuring preferences for health care interventions using conjoint analysis: an application to HIV testing. Health Serv Res 2002;**37**(6):1681-705.
44. de Bekker-Grob EW, Ryan M, Gerard K. Discrete choice experiments in health economics: a review of the literature. Health Econ 2012;**21**(2):145-72.

For peer review only

For peer review only - http://bmjopen.bmj.com/site/about/guidelines.xhtml

FIGURE LEGENDS

Figure 1 – Stages in Conducting a Discrete Choice Experiment

Figure 2 – Sample Choice Set from the Discrete Choice Experiment

For peer review only

Table 1 – Summary of Attributes and Levels Included in the Study

Attribute / Level	A1. How you Test	A2. Time to Result	A3. Accuracy	A4. Consultation Method	A5. Access to HCP	A6. How you get Antibiotics
L1.	Self-test	30 mins	2 in 100 (False Negative)	Online Consultation	Phone	Post to Home
L2.	Self-Sample & Post	2 Hours	5 in 100 (False Negative)	Pharmacy	IM	Post to Collection Point
L3.	Self- Sample & Pharmacy	7 Days		GP	Email	Collect from Pharmacy
L4.	Self-Sample & Education/ Work	14 Days		Sexual Health Clinic	Face to Face	Collect from Sexual Health Clinic
L5.	GP Practice – Sample taken by healthcare professional					
L6.	Sexual Health Clinic – Sample taken by healthcare professional					

For peer review only - http://bmjopen.bmj.com/site/about/guidelines.xhtml

Table 2 – Dummy Coding Reference Level

Attribute	Reference Level
How you test	Sexual Health Clinic
Time to result	7 Days
Accuracy	5 in 100 (False Negative)
Consultation method	Sexual Health Clinic
Access to HCP	Face to Face
How you get antibiotics	Collect from Sexual Health Clinic

For peer review only - http://bmjopen.bmj.com/site/about/guidelines.xhtml

Table 3 – Odds Ratios for all Sub-Groups Included within the Analysis.

*For further information regarding the explanation of attributes and levels to young people in the questionnaire please see **Supplementary File 1**. Odds Ratio values shaded blue are not statistically significant.*

	Full Dataset	Male	Female	16-18	19-21	22-24	Single	One Partner	Prev Test	No Prev Test
n	1,230	607	623	415	406	409	615	512	393	790
	Odds Ratio	Odds Ratio	Odds Ratio	Odds Ratio	Odds Ratio	Odds Ratio	Odds Ratio	Odds Ratio	Odds Ratio	Odds Ratio
**How you Test**										
Self-Test	1.618	1.549	1.693	1.664	1.346	1.895	1.632	1.712	1.524	1.678
Self-Sample and post to a laboratory for analysis	1.358	1.308	1.411	1.477	1.138	1.487	1.380	1.380	1.178	1.465
Self-Sample and take to a pharmacy for analysis	1.155	1.131	1.180	1.270	1.021	1.185	1.220	1.109	0.945	1.277
Self-Sample and take to place of education/work for analysis	0.821	0.919	0.733	0.804	0.806	0.852	0.851	0.778	0.761	0.861
GP Practice – sample taken by a healthcare professional	1.019	1.049	0.990	1.085	1.035	0.941	0.994	1.066	0.885	1.101
Sexual Health Clinic – sample taken by a healthcare professional	1.000	1.000	1.000	1.000	1.000	1.000	1.000	1.000	1.000	1.000
**Time to Result**										
30 Mins	1.806	1.828	1.786	1.763	1.888	1.772	1.730	1.896	1.808	1.797
2 Hours	1.402	1.365	1.442	1.326	1.548	1.344	1.311	1.516	1.357	1.423
7 Days	1.000	1.000	1.000	1.000	1.000	1.000	1.000	1.000	1.000	1.000
14 Days	0.862	0.844	0.881	0.878	0.882	0.828	0.862	0.862	0.882	0.856
**Accuracy**										
2 in 100 False Negative	3.242	2.951	3.570	3.307	3.161	3.282	3.275	3.305	3.000	3.482
5 in 100 False Negative	1.000	1.000	1.000	1.000	1.000	1.000	1.000	1.000	1.000	1.000
**Consultation Method**										
Online Consultation	1.212	1.187	1.239	1.187	1.200	1.253	1.156	1.285	1.174	1.228
Pharmacy Consultation	1.158	1.106	1.215	1.167	1.209	1.102	1.115	1.225	1.098	1.183
GP Consultation	1.183	1.172	1.194	1.279	1.142	1.133	1.162	1.189	1.112	1.215
Sexual Health Clinic Consultation	1.000	1.000	1.000	1.000	1.000	1.000	1.000	1.000	1.000	1.000
**Access to Healthcare Professional**										
Phone	0.949	0.937	0.962	1.001	0.902	0.946	0.942	0.926	0.947	0.953
Instant Messenger	1.027	0.994	1.062	1.108	0.977	1.001	1.025	0.991	1.045	1.025
Email	1.048	1.034	1.062	1.078	1.038	1.030	1.069	1.001	1.049	1.054
Face to Face	1.000	1.000	1.000	1.000	1.000	1.000	1.000	1.000	1.000	1.000
**How you get Antibiotics**										
Post to Home	1.011	0.996	1.028	0.977	1.010	1.048	0.957	1.055	1.011	1.020
Post to Collection Point	1.031	1.082	0.983	1.010	1.036	1.047	0.968	1.072	1.034	1.035
Collect from Pharmacy	1.075	1.064	1.087	1.039	1.106	1.080	1.070	1.056	1.086	1.078
Collect from Sexual Health Clinic	1.000	1.000	1.000	1.000	1.000	1.000	1.000	1.000	1.000	1.000

Table 4 – Trade-off between Accuracy and Time (Probability of Uptake)

For peer review only - http://bmjopen.bmj.com/site/about/guidelines.xhtml

	Full Dataset	Male	Female	16-18	19-21	22-24	Single	One Partner	Prev Test	No Prev Test
Pathways: (n in 100[1] / time[2])										
5 in 100[1]/ 30min[2]	38%	40%	35%	36%	39%	38%	36%	39%	40%	36%
2 in 100/ 14 days	58%	55%	62%	59%	58%	59%	59%	58%	58%	59%
Not Test	4%	5%	3%	5%	3%	3%	4%	3%	2%	5%
Total	100%	100%	100%	100%	100%	100%	100%	100%	100%	100%

[1] Test accuracy = n in 100 people will be told they don't have chlamydia when they do
[2] Time to receipt of result

For peer review only

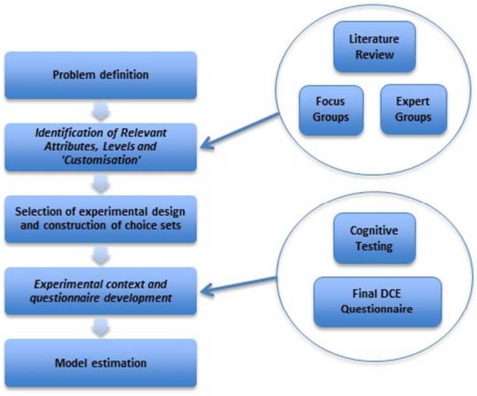

Figure 1 – Stages in Conducting a Discrete Choice Experiment

254x190mm (96 x 96 DPI)

For peer review only - http://bmjopen.bmj.com/site/about/guidelines.xhtml

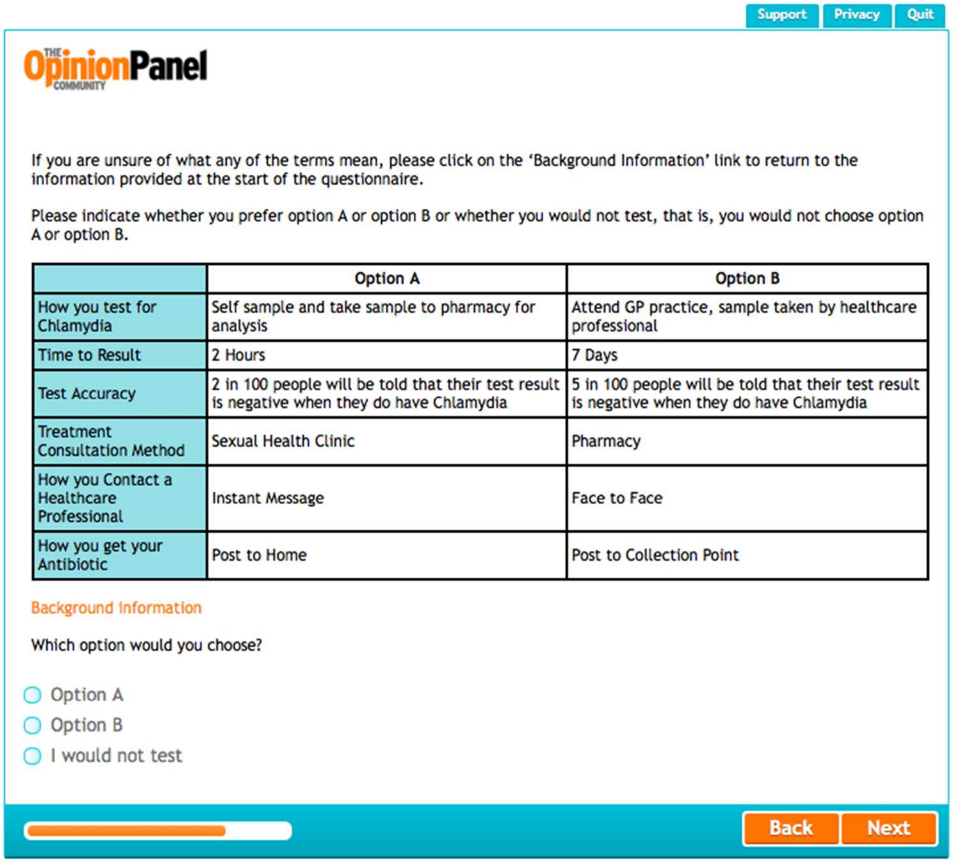

Support Privacy Quit

The Opinion Panel Community

If you are unsure of what any of the terms mean, please click on the 'Background Information' link to return to the information provided at the start of the questionnaire.

Please indicate whether you prefer option A or option B or whether you would not test, that is, you would not choose option A or option B.

	Option A	Option B
How you test for Chlamydia	Self sample and take sample to pharmacy for analysis	Attend GP practice, sample taken by healthcare professional
Time to Result	2 Hours	7 Days
Test Accuracy	2 in 100 people will be told that their test result is negative when they do have Chlamydia	5 in 100 people will be told that their test result is negative when they do have Chlamydia
Treatment Consultation Method	Sexual Health Clinic	Pharmacy
How you Contact a Healthcare Professional	Instant Message	Face to Face
How you get your Antibiotic	Post to Home	Post to Collection Point

Background information

Which option would you choose?

- Option A
- Option B
- I would not test

Back Next

Figure 2 – Sample Choice Set from the Discrete Choice Experiment

265x240mm (72 x 72 DPI)

only

Supplementary File 1 – DCE Questionnaire Introduction and Background Information

Developing Chlamydia Testing & Treatment Services

This survey is designed to find out more about your views on what is important to you in accessing services to get tested for chlamydia, and treatment if your test result is positive (i.e. you have chlamydia). This forms part of a research project being undertaken at the University of Warwick, please click here to access further information about the study and how your information will be used [insert url to participant information leaflet]. By continuing to complete the survey you are consenting for your responses to be used for research in accordance with the information set out in the study information leaflet. Your responses will help us understand what is important to young people to shape how services are developed in the future.

Please read through the following background information before completing the survey. It contains information about chlamydia and the terms that are used in the survey.

What is Chlamydia?

Chlamydia is the most common sexually transmitted infection in England and the majority of infections are found in young people aged 15-24. Both men and women can get chlamydia, but most people with chlamydia have no symptoms and do not know they have an infection. The test for chlamydia is usually a urine sample for men and a vaginal swab for women. Once diagnosed, chlamydia can be treated with a single dose (2 or 4 tablets) of an antibiotic called Azithromycin.

In England there is a national chlamydia screening programme which recommends that people aged 16-24 are tested for chlamydia annually or when they change their sexual partner.

What happens if I don't get treatment?

If left undiagnosed chlamydia can lead to serious health problems.

In women this includes a condition called Pelvic Inflammatory Disease, which is an infection of the womb, fallopian tubes or ovaries. This can cause severe pain and in some cases (around 1 in 10) lead to infertility or ectopic pregnancy (where the pregnancy occurs outside of the womb in the fallopian tube). If you are pregnant and have chlamydia you can pass it on to your baby when you give birth and this can lead to the baby getting an eye infection or pneumonia.

In men chlamydia can lead to a condition called epididymitis, which causes soreness and swelling in the scrotum.

For peer review only

For further information

If you would like further information on chlamydia and how to prevent it, or any of the other conditions described please visit:
http://www.nhs.uk/conditions/chlamydia/Pages/Introduction.aspx

About the Survey...

In completing the survey you will be presented with a number of choices for getting tested and treatment for chlamydia. Please consider each set of choices and indicate whether you prefer option A or option B or whether you would not test, that is, you would not choose option A or option B.

Each choice involves two different scenarios for testing and getting treatment and there are differences between how you test, how long you have to wait for the result, how accurate the test is, how you have a consultation to get treatment for chlamydia, how you access a healthcare professional to get that treatment and how you get your antibiotic.

The following sections provide you with an explanation of the process of getting tested and treatment and the key terms used in the survey:

The diagram below shows the stages up until you find out our result:

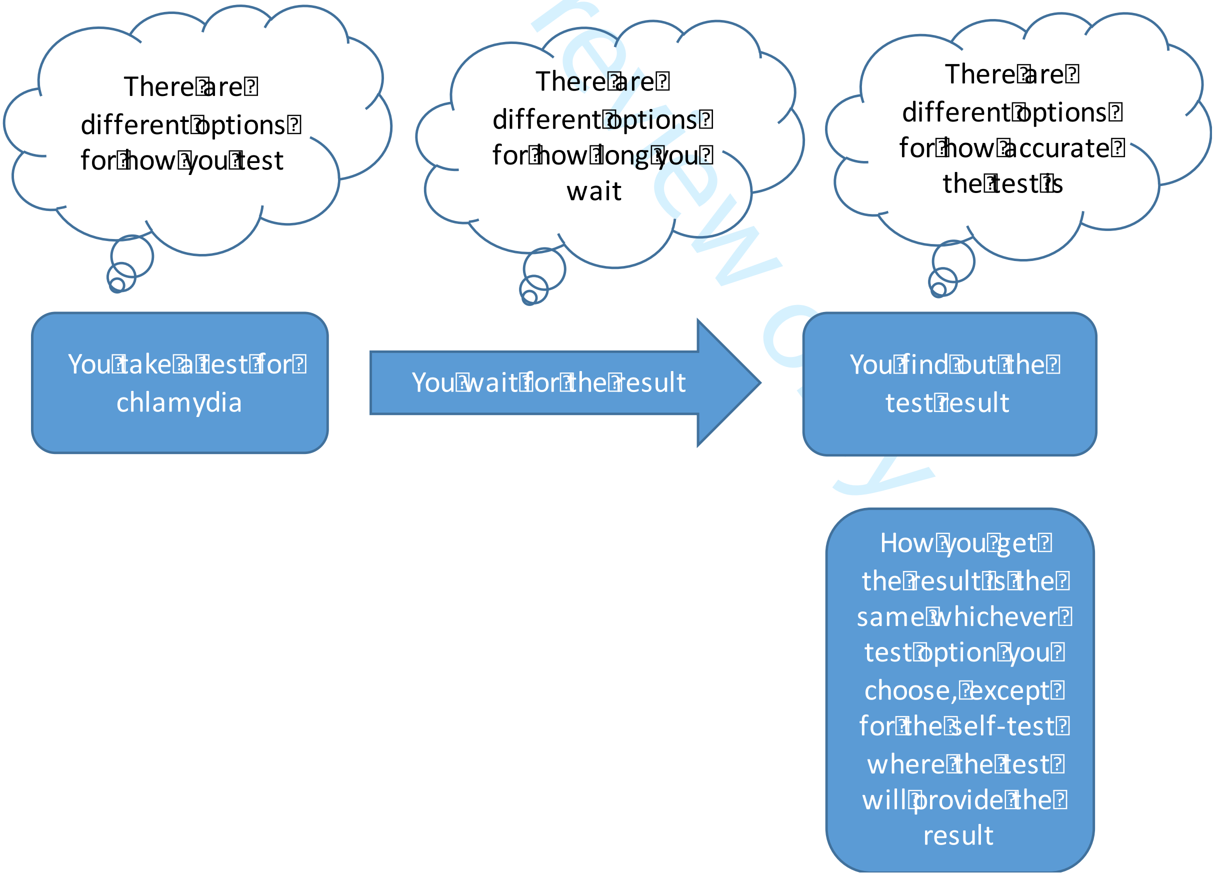

The following sections provide more information on how you test, how long you wait for the result and the accuracy of the test.

For peer review only

How you test for Chlamydia

This focuses on how you get your test, how the sample is taken and what happens to the sample once you've taken it. There are six options in the questionnaire, an explanation of these is provided below:

- **Self-Test -** Order a test kit online or collect one from a community location e.g. pharmacy or supermarket, provide the sample yourself and interpret the result yourself (like a pregnancy test). This is different to the self-sample options below because you interpret the result yourself rather than it being interpreted by a healthcare professional.
- **Self-Sample and post off for analysis -** Order a test kit online or collect one from a community location e.g. pharmacy or supermarket, provide the sample yourself and send the sample in the freepost envelope to the laboratory for analysis. This is different to the self-test option above because the test is interpreted by a healthcare professional.
- **Self-Sample and take to pharmacy for analysis -** Order a test kit online or collect one from a community location e.g. pharmacy or supermarket, provide the sample yourself and then take the sample to a pharmacy for analysis. This is different to the self-test option above because the test is interpreted by a healthcare professional. Opening hours vary between pharmacies but many in towns and cities are open evenings and weekends as well as weekdays.
- **Self-Sample and take to your place of education/ workplace for analysis -** Order a test kit online or collect one from a community location e.g. pharmacy or supermarket, provide the sample yourself and take the sample to your place of education/ workplace for analysis by an outreach nurse. This is different to the self-test option above because the test is interpreted by a healthcare professional. Outreach services in education and workplaces are usually available one day a week.
- **Attend GP Practice, sample taken by a GP or Nurse -** Book an appointment at your GP practice with a GP or practice nurse. Your sample will be taken by the healthcare professional you see. GP practices are routinely open Monday to Friday 8am – 6.30pm.
- **Attend sexual health clinic, sample taken by a Doctor or Nurse -** Drop in or book an appointment with a doctor or nurse at a sexual health clinic. Your sample will be taken by the health care professional you see. Sexual Health Clinics are located in towns and cities, opening hours vary but clinics are generally open during the working day Monday to Friday, some evenings during the week and Saturdays.

Taking a sample yourself is straightforward and does not affect the accuracy of the test result.

Time to Result

How long it takes to get the test result. For the self-test option, this is how long it takes from you doing the test to you being able to read the result. For the other options this is how

For peer review only

long it takes from you posting or dropping off your sample to when you are given your result. There are four options:

- 30 Mins
- 2 Hours
- 7 Days
- 14 Days

The time it takes to get the result does not affect the accuracy of the test.

Test Accuracy

This tells you how accurate your test result is. In this survey we want you to consider the likelihood of the test telling you that you don't have chlamydia when in fact you do. There are two options:

1. 2 in 100 people will be told that their test result is negative when they do have chlamydia
2. 5 in 100 people will be told their test result is negative when they do have chlamydia

Another way of describing this is shown below:

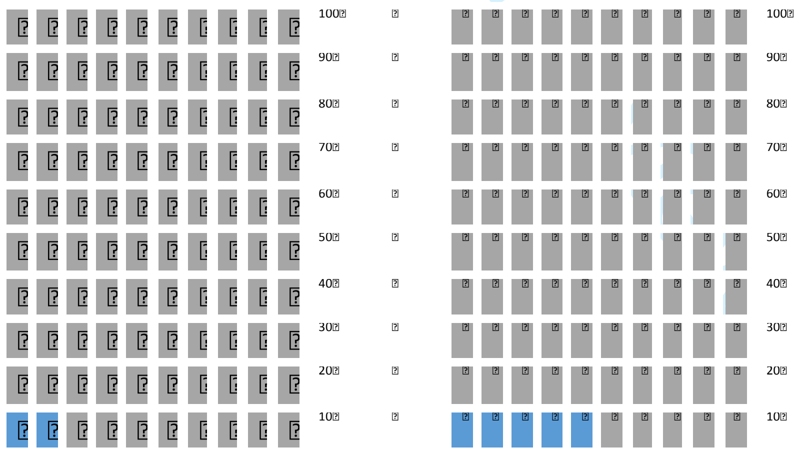

Each graph represents 100 people

= People who are told their test result is negative when they do have chlamydia

We would also like you to think about how you would choose to get treatment if your test result is positive. The diagram below shows the stages to get treatment:

For peer review only

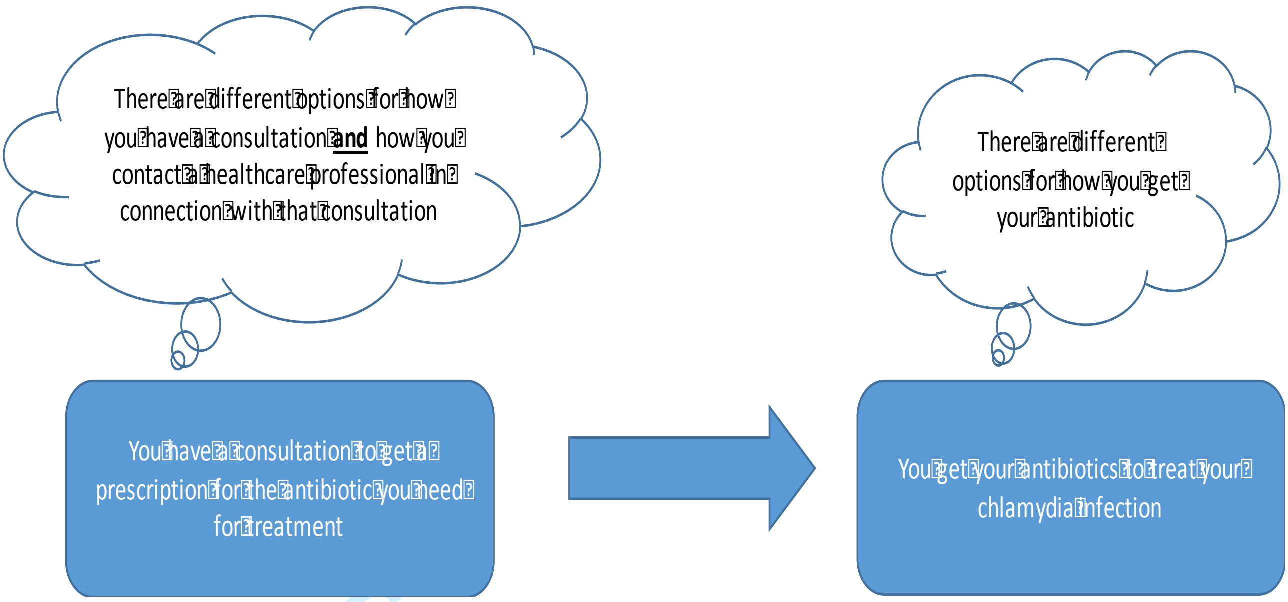

The following sections provide more information on how you get your treatment, how you contact a healthcare professional and how you get your antibiotics.

How you get your treatment

If your test result is positive there are a number of different options for you to get your treatment for chlamydia. There are four options in this survey:

- Complete an online consultation – this is available 24 hours a day, 7 days a week. You can access a healthcare professional if you need any help or advice between 9am and 5pm Monday to Friday.
- Have a consultation with a pharmacist - Opening hours vary between pharmacies but many in towns and cities are open evenings and weekends as well as weekdays.
- Have a consultation with a GP - GP practices are routinely open Monday to Friday 8am – 6.30pm.
- Have a consultation with a doctor or nurse at a Sexual Health Clinic - Sexual Health Clinics are located in towns and cities, opening hours vary but clinics are generally open during the working day Monday to Friday, some evenings during the week and Saturdays.

How you Contact a Healthcare Professional

New technology means that there are now more options for completing your consultation to get treatment, or in the case of the online consultation, accessing a healthcare professional for advice. There are four options within the questionnaire:

- Telephone – speak to a healthcare professional on the telephone.
- Instant Messaging – have a discussion with a healthcare professional via an instant messaging service online
- Email – have a discussion with a healthcare professional via email. During opening hours you will receive a response within 2 hours, and for emails sent out of hours you will receive a response the next day.
- Face-to-face - have a face-to-face discussion with a healthcare professional.

For peer review only

How you get your antibiotics

Once you've completed your consultation you need to get your antibiotic tablets. In this survey there are four options available to you:

- Deliver to home address – provide your home address for your antibiotic to be posted to you. Your antibiotic will arrive within 1-2 working days.
- Deliver to collection point – nominate an address other than your home address, for example a friend's address or a collection point for your antibiotic to be posted to. Your antibiotic will arrive within 1-2 working days.
- Collect from Pharmacy – attend a pharmacy of your choice with your prescription and the pharmacist gives you the antibiotic tablets
- Collect from Sexual Health Clinic – attend a sexual health clinic and the doctor or nurse gives you the antibiotic tablets

In all of the choices available to you there are a number of things that you should assume are the same:

- The service is provided to you free of charge although there may be a cost to you, for example in making phone calls, accessing the internet, or taking time off work to go to an appointment.
- The healthcare professional who is providing your treatment is trained to be able to provide the service.
- How you get your results is the same whichever option you choose, except for the self-test where the test will provide the result.
- Your personal data is managed securely in whichever option you choose

More choices for an option does not mean that it is more or less important, it just reflects that there are more choices within the scenarios you are being asked to consider.

For peer review only

Supplementary File 2 – DCE Results

Part 1 - Demographic Characteristics for Sub-Group Analysis

Demographic Characteristic	n	%	National %[1]
**Age**			
16	8	1%	10%
17	113	9%	10%
18	294	24%	11%
19	183	15%	11%
20	132	11%	11%
21	91	7%	11%
22	162	13%	11%
23	135	11%	12%
24	112	9%	12%
Total	1230	100%	99.00%
**Gender**			
Male	607	49%	51%
Female	623	51%	49%
Total	1230	100%	100%
**Ethnicity**			
White – English, Welsh, Scottish, Northern Irish, British	932	76%	80%
White – Irish	7	1%	1%
White – Gypsy or Irish Traveller	1	0%	0%
White – Any other white background	37	3%	5%
Mixed/ Multiple Ethnic Groups – White & Black Caribbean	10	1%	1%
Mixed/ Multiple Ethnic Groups – White & Black African	6	0%	0%
Mixed/ Multiple Ethnic Groups – White & Asian	13	1%	1%
Mixed/ Multiple Ethnic Groups – Any other mixed/ multiple ethnic background	8	1%	1%
Asian/ Asian British – Indian	50	4%	3%
Asian/ Asian British - Pakistani	38	3%	2%
Asian/ Asian British - Bangladeshi	18	1%	1%
Asian/ Asian British - Chinese	22	2%	1%
Asian/ Asian British – Any other Asian background	21	2%	2%
Black/ African/ Caribbean/ Black British – African	26	2%	2%
Black/ African/ Caribbean/ Black British – Caribbean	10	1%	1%

[1] - National Data for Age and Region taken from ONS Mid-Year Population Estimates 2015 (ONS, 2016b). Percentages derived from the total 16-24 population. National Data for Ethnicity taken from Census 2011 data, percentages derived from total England population (ONS, 2012). Percentages may not sum due to rounding

Demographic Characteristic	n	%	National %[1]
Black/ African/ Caribbean/ Black British – Any other black/ African/ Caribbean background	2	0%	1%
Other – Arab	5	0%	0%
Other – Any other ethnic group	9	1%	1%
I would prefer not to say	15	1%	0%
Total	1230	100%	103.00%
**Region**			
East Midlands	117	10%	9%
London	225	18%	15%
North East	74	6%	5%
North West	123	10%	13%
Eastern	55	4%	10%
South East	222	18%	16%
South West	143	12%	10%
West Midlands	144	12%	11%
Yorkshire and The Humber	113	9%	11%
I would prefer not to say	14	1%	0%
Total	1230	100%	100.00%
**Sexual Preference**			
Heterosexual (partner of opposite sex)	1030	84%	
Homosexual (partner of same sex) & Male	13	1%	
Homosexual (partner of same sex) & Female	39	3%	
Bisexual (partner of either sex)	98	8%	
I would prefer not to say	50	4%	
Total	1230	100%	
**Previous STI Test**			
Yes	393	32%	
No	790	64%	
I would prefer not to say	47	4%	
Total	1230	100%	
**Relationship Status**			
Single	615	50%	
In a non-sexual relationship with one person	36	3%	
In a non-sexual relationship with more than one person	2	0%	
In a sexual relationship with one person	512	42%	
In a sexual relationship with more than one person	36	3%	
I would prefer not to say	29	2%	
Total	1230	100%	

Part 2 - Validity Checks

Table 2.1 – Comparison of Full Dataset Coefficients and Odds Ratios for the Full Dataset for Dataset including Choice Set 1 and Dataset including Choice Set 25. Data highlighted in blue indicate results which are not statistically significant.

	**Dataset Including Choice Set 1, *n=1,230***						**Dataset Including Choice Set 25, *n=1,230***					
	**Coefficient**	**Lower 95% CI**	**Upper 95% CI**	**Odds Ratio**	**Lower 95% CI**	**Upper 95% CI**	**Coefficient**	**Lower 95% CI**	**Upper 95% CI**	**Odds Ratio**	**Lower 95% CI**	**Upper 95% CI**
**How you Test**												
Self-Test	0.498	0.432	0.564	1.646	1.540	1.758	0.481	0.415	0.547	1.618	1.514	1.729
Post	0.308	0.239	0.376	1.360	1.270	1.456	0.306	0.239	0.373	1.358	1.271	1.452
Pharmacy	0.116	0.056	0.175	1.123	1.057	1.192	0.144	0.085	0.203	1.155	1.088	1.226
Education/ Work	-0.206	-0.266	-0.146	0.814	0.766	0.864	-0.197	-0.257	-0.137	0.821	0.773	0.872
GP Practice	0.011	-0.050	0.073	1.011	0.951	1.075	0.019	-0.042	0.081	1.019	0.959	1.084
Sexual Health Clinic	0.000	-	-	1.000	-	-	0.000	-	-	1.000	-	-
**Time to Result**												
30 Mins	0.579	0.524	0.633	1.784	1.688	1.884	0.591	0.537	0.645	1.806	1.711	1.906
2 Hours	0.340	0.283	0.398	1.406	1.328	1.488	0.338	0.281	0.395	1.402	1.324	1.485
7 Days	0.000	-	-	1.000	-	-	0.000	-	-	1.000	-	-
14 Days	-0.149	-0.200	-0.099	0.861	0.819	0.906	-0.148	-0.198	-0.098	0.862	0.820	0.907
**Accuracy**												
2 in 100 False Negative	1.192	1.157	1.226	3.292	3.179	3.409	1.176	1.141	1.212	3.242	3.130	3.359
5 in 100 False Negative	0.000	-	-	1.000	-	-	0.000	-	-	1.000	-	-

For peer review only

	Dataset Including Choice Set 1, *n=1,230*						Dataset Including Choice Set 25, *n=1,230*					
	**Coefficient**	**Lower 95% CI**	**Upper 95% CI**	**Odds Ratio**	**Lower 95% CI**	**Upper 95% CI**	**Coefficient**	**Lower 95% CI**	**Upper 95% CI**	**Odds Ratio**	**Lower 95% CI**	**Upper 95% CI**
**Consultation Method**												
Online Consultation	0.185	0.133	0.236	1.203	1.142	1.266	0.192	0.140	0.245	1.212	1.150	1.277
Pharmacy Consultation	0.150	0.099	0.202	1.162	1.104	1.224	0.147	0.095	0.199	1.158	1.100	1.220
GP Consultation	0.146	0.095	0.197	1.157	1.100	1.218	0.168	0.116	0.220	1.183	1.123	1.246
Sexual Health Clinic Consultation	0.000	-	-	1.000	-	-	0.000	-	-	1.000	-	-
**Access to Healthcare Professional**												
Phone	-0.058	-0.108	-0.008	0.943	0.897	0.992	-0.052	-0.102	-0.002	0.949	0.903	0.998
Instant Messenger	0.020	-0.029	0.070	1.020	0.971	1.072	0.027	-0.023	0.076	1.027	0.977	1.079
Email	0.027	-0.023	0.076	1.027	0.978	1.079	0.047	-0.002	0.096	1.048	0.998	1.101
Face to Face	0.000	-	-	1.000	-	-	0.000	-	-	1.000	-	-
**How you get Antibiotics**												
Post to Home	0.038	-0.013	0.089	1.039	0.987	1.093	0.011	-0.041	0.063	1.011	0.960	1.065
Post to Collection Point	0.052	0.002	0.101	1.053	1.002	1.106	0.030	-0.020	0.081	1.031	0.980	1.085
Collect from Pharmacy	0.091	0.038	0.143	1.095	1.039	1.154	0.072	0.018	0.126	1.075	1.018	1.134
Collect from Sexual Health Clinic	0.000	-	-	1.000	-	-	0.000	-	-	1.000	-	-
**Choice**												
Option A	0.000	-	-	1.000	-	-	0.000	-	-	1.000	-	-
Option B	-0.341	-0.381	-0.302	0.711	0.683	0.740	-0.360	-0.400	-0.320	0.698	0.670	0.726
I would not test	-1.697	-1.797	-1.596	0.183	0.166	0.203	-1.682	-1.781	-1.583	0.186	0.168	0.205

Table 2.2 – Coefficients and Odds Ratios for the Full Dataset and the Dataset Excluding Different Responses to Choice Sets 1 and 25. Data highlighted in blue indicate results which are not statistically significant.

	**Full Dataset *n=1,230***						**Excl Incorrect Answer to Validity Check *n=912***					
	**Coefficient**	**Lower 95% CI**	**Upper 95% CI**	**Odds Ratio**	**Lower 95% CI**	**Upper 95% CI**	**Coefficient**	**Lower 95% CI**	**Upper 95% CI**	**Odds Ratio**	**Lower 95% CI**	**Upper 95% CI**
**How you Test**												
Self-Test	0.481	0.415	0.547	1.618	1.514	1.729	0.507	0.429	0.584	1.660	1.535	1.794
Post	0.306	0.239	0.373	1.358	1.271	1.452	0.333	0.254	0.411	1.395	1.290	1.508
Pharmacy	0.144	0.085	0.203	1.155	1.088	1.226	0.124	0.054	0.194	1.132	1.056	1.214
Education/ Work	-0.197	-0.257	-0.137	0.821	0.773	0.872	-0.253	-0.323	-0.183	0.777	0.724	0.833
GP Practice	0.019	-0.042	0.081	1.019	0.959	1.084	0.019	-0.053	0.091	1.019	0.948	1.095
Sexual Health Clinic	0.000	-	-	1.000	-	-	0.000	-	-	1.000	-	-
**Time to Result**												
30 Mins	0.591	0.537	0.645	1.806	1.711	1.906	0.573	0.510	0.637	1.774	1.665	1.890
2 Hours	0.338	0.281	0.395	1.402	1.324	1.485	0.335	0.268	0.402	1.397	1.307	1.494
7 Days	0.000	-	-	1.000	-	-	0.000	-	-	1.000	-	-
14 Days	-0.148	-0.198	-0.098	0.862	0.820	0.907	-0.148	-0.177	-0.236	-0.117	0.790	0.889
**Accuracy**												
2 in 100 False Negative	1.176	1.141	1.212	3.242	3.130	3.359	1.323	1.282	1.364	3.755	3.602	3.913
5 in 100 False Negative	0.000	-	-	1.000	-	-	0.000	-	-	1.000	-	-
**Consultation Method**												
Online Consultation	0.192	0.140	0.245	1.212	1.150	1.277	0.197	0.136	0.258	1.218	1.145	1.294
Pharmacy Consultation	0.147	0.095	0.199	1.158	1.100	1.220	0.173	0.112	0.234	1.189	1.119	1.264
GP Consultation	0.168	0.116	0.220	1.183	1.123	1.246	0.179	0.119	0.240	1.196	1.126	1.271
Sexual Health Clinic Consultation	0.000	-	-	1.000	-	-	0.000	-	-	1.000	-	-

For peer review only

	**Full Dataset** ***n=1,230***						**Excl Incorrect Answer to Validity Check** ***n=912***					
	**Coefficient**	**Lower 95% CI**	**Upper 95% CI**	**Odds Ratio**	**Lower 95% CI**	**Upper 95% CI**	**Coefficient**	**Lower 95% CI**	**Upper 95% CI**	**Odds Ratio**	**Lower 95% CI**	**Upper 95% CI**
**Access to Healthcare Professional**												
Phone	-0.052	-0.102	-0.002	0.949	0.903	0.998	-0.084	-0.143	-0.026	0.919	0.867	0.975
Instant Messenger	0.027	-0.023	0.076	1.027	0.977	1.079	-0.026	-0.084	0.031	0.974	0.919	1.032
Email	0.047	-0.002	0.096	1.048	0.998	1.101	-0.005	-0.063	0.052	0.995	0.939	1.054
Face to Face	0.000	-	-	1.000	-	-	0.000	-	-	1.000	-	-
**How you get Antibiotics**												
Post to Home	0.011	-0.041	0.063	1.011	0.960	1.065	0.014	-0.047	0.075	1.014	0.954	1.078
Post to Collection Point	0.030	-0.020	0.081	1.031	0.980	1.085	0.023	-0.036	0.083	1.024	0.964	1.086
Collect from Pharmacy	0.072	0.018	0.126	1.075	1.018	1.134	0.065	0.001	0.128	1.067	1.001	1.137
Collect from Sexual Health Clinic	0.000	-	-	1.000	-	-	0.000	-	-	1.000	-	-
**Choice**												
Option A	0.000	-	-	1.000	-	-	0.000	-	-	1.000	-	-
Option B	-0.360	-0.400	-0.320	0.698	0.670	0.726	-0.327	-0.374	-0.279	0.721	0.688	0.756
I would not test	-1.682	-1.781	-1.583	0.186	0.168	0.205	-1.674	-1.790	-1.557	0.188	0.167	0.211

For peer review only

Table 2.3 – Coefficient and Odds Ratios for the Full Dataset and the Dataset Containing Five Minutes or Longer to Complete. Data highlighted in blue indicate results which are not statistically significant.

	**Full Dataset *n=1,230***						**Data Excluding Responses taking less than 5 min to complete *n= 997***					
	**Coefficient**	**Lower 95% CI**	**Upper 95% CI**	**Odds Ratio**	**Lower 95% CI**	**Upper 95% CI**	**Coefficient**	**Lower 95% CI**	**Upper 95% CI**	**Odds Ratio**	**Lower 95% CI**	**Upper 95% CI**
**How you Test**												
Self-Test	0.481	0.415	0.547	1.618	1.514	1.729	0.542	0.467	0.616	1.719	1.595	1.852
Post	0.306	0.239	0.373	1.358	1.271	1.452	0.344	0.269	0.419	1.411	1.309	1.521
Pharmacy	0.144	0.085	0.203	1.155	1.088	1.226	0.168	0.101	0.234	1.183	1.106	1.264
Education/ Work	-0.197	-0.257	-0.137	0.821	0.773	0.872	-0.245	-0.312	-0.177	0.783	0.732	0.837
GP Practice	0.019	-0.042	0.081	1.019	0.959	1.084	-0.008	-0.077	0.061	0.992	0.925	1.062
Sexual Health Clinic	0.000	-	-	1.000	-	-	0.000	-	-	1.000	-	-
**Time to Result**												
30 Mins	0.591	0.537	0.645	1.806	1.711	1.906	0.642	0.581	0.703	1.900	1.788	2.019
2 Hours	0.338	0.281	0.395	1.402	1.324	1.485	0.372	0.308	0.436	1.451	1.361	1.547
7 Days	0.000	-	-	1.000	-	-	0.000	-	-	1.000	-	-
14 Days	-0.148	-0.198	-0.098	0.862	0.820	0.907	-0.147	-0.204	-0.091	0.863	0.816	0.913
**Accuracy**												
2 in 100 False Negative	1.176	1.141	1.212	3.242	3.130	3.359	1.308	1.269	1.348	3.700	3.556	3.850
5 in 100 False Negative	0.000	-	-	1.000	-	-	0.000	-	-	1.000	-	-
**Consultation Method**												
Online Consultation	0.192	0.140	0.245	1.212	1.150	1.277	0.217	0.159	0.276	1.243	1.172	1.318
Pharmacy Consultation	0.147	0.095	0.199	1.158	1.100	1.220	0.160	0.102	0.219	1.174	1.108	1.245
GP Consultation	0.168	0.116	0.220	1.183	1.123	1.246	0.171	0.113	0.229	1.187	1.120	1.258
Sexual Health Clinic Consultation	0.000	-	-	1.000	-	-	0.000	-	-	1.000	-	-
**Access to Healthcare Professional**												
Phone	-0.052	-0.102	-0.002	0.949	0.903	0.998	-0.042	-0.098	0.014	0.959	0.906	1.014
Instant Messenger	0.027	-0.023	0.076	1.027	0.977	1.079	0.043	-0.013	0.098	1.044	0.987	1.103
Email	0.047	-0.002	0.096	1.048	0.998	1.101	0.042	-0.013	0.097	1.043	0.987	1.102
Face to Face	0.000	-	-	1.000	-	-	0.000	-	-	1.000	-	-

For peer review only

	Full Dataset *n=1,230*						Data Excluding Responses taking less than 5 min to complete *n= 997*					
	Coefficient	Lower 95% CI	Upper 95% CI	Odds Ratio	Lower 95% CI	Upper 95% CI	Coefficient	Lower 95% CI	Upper 95% CI	Odds Ratio	Lower 95% CI	Upper 95% CI
**How you get Antibiotics**												
Post to Home	0.011	-0.041	0.063	1.011	0.960	1.065	0.001	-0.057	0.060	1.001	0.944	1.062
Post to Collection Point	0.030	-0.020	0.081	1.031	0.980	1.085	0.013	-0.044	0.070	1.013	0.957	1.072
Collect from Pharmacy	0.072	0.018	0.126	1.075	1.018	1.134	0.085	0.024	0.145	1.089	1.025	1.157
Collect from Sexual Health Clinic	0.000	-	-	1.000	-	-	0.000	-	-	1.000	-	-
**Choice**												
Option A	0.000	-	-	1.000	-	-	0.000	-	-	1.000	-	-
Option B	-0.360	-0.400	-0.320	0.698	0.670	0.726	-0.368	-0.413	-0.323	0.692	0.662	0.724
I would not test	-1.682	-1.781	-1.583	0.186	0.168	0.205	-1.776	-1.889	-1.662	0.169	0.151	0.190

For peer review only

Table 2.4 – Comparison of Full Dataset and Dataset Excluding Opt Out Responses. Data highlighted in blue indicate results which are not statistically significant.

	**Full Dataset** ***n=29,520 Choice Responses***						**Removal of 'I would not test' Data** ***n=27,727 Choice Responses***					
	**Coefficient**	**Lower 95% CI**	**Upper 95% CI**	**Odds Ratio**	**Lower 95% CI**	**Upper 95% CI**	**Coefficient**	**Lower 95% CI**	**Upper 95% CI**	**Odds Ratio**	**Lower 95% CI**	**Upper 95% CI**
**How you Test**												
Self-Test	0.481	0.415	0.547	1.618	1.514	1.729	0.501	0.433	0.568	1.650	1.542	1.765
Post	0.306	0.239	0.373	1.358	1.271	1.452	0.320	0.252	0.387	1.377	1.287	1.473
Pharmacy	0.144	0.085	0.203	1.155	1.088	1.226	0.152	0.092	0.212	1.164	1.096	1.236
Education/ Work	-0.197	-0.257	-0.137	0.821	0.773	0.872	-0.202	-0.262	-0.141	0.817	0.769	0.868
GP Practice	0.019	-0.042	0.081	1.019	0.959	1.084	0.027	-0.035	0.089	1.027	0.965	1.093
Sexual Health Clinic	0.000	-	-	1.000	-	-	0.000	-	-	1.000	-	-
**Time to Result**												
30 Mins	0.591	0.537	0.645	1.806	1.711	1.906	0.612	0.558	0.667	1.845	1.746	1.949
2 Hours	0.338	0.281	0.395	1.402	1.324	1.485	0.350	0.292	0.408	1.419	1.339	1.503
7 Days	0.000	-	-	1.000	-	-	0.000	-	-	1.000	-	-
14 Days	-0.148	-0.198	-0.098	0.862	0.820	0.907	-0.145	-0.196	-0.094	0.865	0.822	0.910
**Accuracy**												
2 in 100 False Negative	1.176	1.141	1.212	3.242	3.130	3.359	1.205	1.170	1.241	3.338	3.221	3.460
5 in 100 False Negative	0.000	-	-	1.000	-	-	0.000	-	-	1.000	-	-
**Consultation Method**												
Online Consultation	0.192	0.140	0.245	1.212	1.150	1.277	0.200	0.147	0.253	1.222	1.159	1.288
Pharmacy Consultation	0.147	0.095	0.199	1.158	1.100	1.220	0.159	0.106	0.211	1.172	1.112	1.235
GP Consultation	0.168	0.116	0.220	1.183	1.123	1.246	0.174	0.122	0.227	1.190	1.130	1.254
Sexual Health Clinic Consultation	0.000	-	-	1.000	-	-	0.000	-	-	1.000	-	-
**Access to Healthcare Professional**												
Phone	-0.052	-0.102	-0.002	0.949	0.903	0.998	-0.046	-0.097	0.004	0.955	0.908	1.004
Instant Messenger	0.027	-0.023	0.076	1.027	0.977	1.079	0.029	-0.021	0.079	1.029	0.979	1.082
Email	0.047	-0.002	0.096	1.048	0.998	1.101	0.050	0.000	0.100	1.051	1.000	1.105
Face to Face	0.000	-	-	1.000	-	-	0.000	-	-	1.000	-	-

For peer review only

	Full Dataset *n=29,520 Choice Responses*						Removal of 'I would not test' Data *n=27,727 Choice Responses*					
	**Coefficient**	**Lower 95% CI**	**Upper 95% CI**	**Odds Ratio**	**Lower 95% CI**	**Upper 95% CI**	**Coefficient**	**Lower 95% CI**	**Upper 95% CI**	**Odds Ratio**	**Lower 95% CI**	**Upper 95% CI**
**How you get Antibiotics**												
Post to Home	0.011	-0.041	0.063	1.011	0.960	1.065	0.012	-0.041	0.065	1.012	0.960	1.067
Post to Collection Point	0.030	-0.020	0.081	1.031	0.980	1.085	0.035	-0.016	0.086	1.036	0.984	1.090
Collect from Pharmacy	0.072	0.018	0.126	1.075	1.018	1.134	0.076	0.021	0.131	1.079	1.022	1.139
Collect from Sexual Health Clinic	0.000	-	-	1.000	-	-	0.000	-	-	1.000	-	-
**Choice**												
Option A	0.000	-	-	1.000	-	-	0.000	-	-	1.000	-	-
Option B	-0.360	-0.400	-0.320	0.698	0.670	0.726	-0.368	-0.409	-0.327	0.692	0.664	0.721
I would not test	-1.682	-1.781	-1.583	0.186	0.168	0.205	-	-	-	1.000		

For peer review only

Part 3 – DCE Results

(Cells highlighted in blue indicate result which is not statistically significant)

Table 3.1- Full Dataset Coefficients, Odds Ratios and Respective 95% Confidence Interval

	Full Dataset *n=1,230*					
	Coefficient	Lower 95% CI	Upper 95% CI	Odds Ratio	Lower 95% CI	Upper 95% CI
**How you Test**						
Self-Test	0.481	0.415	0.547	1.618	1.514	1.729
Post	0.306	0.239	0.373	1.358	1.271	1.452
Pharmacy	0.144	0.085	0.203	1.155	1.088	1.226
Education/ Work	-0.197	-0.257	-0.137	0.821	0.773	0.872
GP Practice	0.019	-0.042	0.081	1.019	0.959	1.084
Sexual Health Clinic	0.000	-	-	1.000	-	-
**Time to Result**						
30 Mins	0.591	0.537	0.645	1.806	1.711	1.906
2 Hours	0.338	0.281	0.395	1.402	1.324	1.485
7 Days	0.000	-	-	1.000	-	-
14 Days	-0.148	-0.198	-0.098	0.862	0.820	0.907
**Accuracy**						
2 in 100 False Negative	1.176	1.141	1.212	3.242	3.130	3.359
5 in 100 False Negative	0.000	-	-	1.000	-	-
**Consultation Method**						
Online Consultation	0.192	0.140	0.245	1.212	1.150	1.277
Pharmacy Consultation	0.147	0.095	0.199	1.158	1.100	1.220
GP Consultation	0.168	0.116	0.220	1.183	1.123	1.246
Sexual Health Clinic Consultation	0.000	-	-	1.000	-	-
**Access to Healthcare Professional**						
Phone	-0.052	-0.102	-0.002	0.949	0.903	0.998
Instant Messenger	0.027	-0.023	0.076	1.027	0.977	1.079
Email	0.047	-0.002	0.096	1.048	0.998	1.101
Face to Face	0.000	-	-	1.000	-	-
**How you get Antibiotics**						
Post to Home	0.011	-0.041	0.063	1.011	0.960	1.065
Post to Collection Point	0.030	-0.020	0.081	1.031	0.980	1.085
Collect from Pharmacy	0.072	0.018	0.126	1.075	1.018	1.134
Collect from Sexual Health Clinic	0.000	-	-	1.000	-	-

For peer review only

Table 3.2 - Gender Subgroup Analysis Coefficients and Odds Ratios with Associated 95% Confidence Intervals

	**Males *n=607***						**Females *n=623***					
	**Coefficient**	**Lower 95% CI**	**Upper 95% CI**	**Odds Ratio**	**Lower 95% CI**	**Upper 95% CI**	**Coefficient**	**Lower 95% CI**	**Upper 95% CI**	**Odds Ratio**	**Lower 95% CI**	**Upper 95% CI**
**How you Test**												
Self-Test	0.438	0.344	0.531	1.549	1.410	1.701	0.526	0.432	0.620	1.693	1.541	1.860
Post	0.269	0.174	0.363	1.308	1.190	1.438	0.344	0.250	0.439	1.411	1.284	1.551
Pharmacy	0.123	0.039	0.208	1.131	1.040	1.231	0.165	0.081	0.249	1.180	1.085	1.283
Education/ Work	-0.084	-0.169	0.001	0.919	0.845	1.001	-0.310	-0.395	-0.225	0.733	0.674	0.798
GP Practice	0.048	-0.039	0.135	1.049	0.962	1.145	-0.010	-0.097	0.077	0.990	0.908	1.080
Sexual Health Clinic	0.000	-	-	1.000	-	-	0.000	-	-	1.000	-	-
**Time to Result**												
30 Mins	0.603	0.527	0.680	1.828	1.694	1.973	0.580	0.503	0.656	1.786	1.654	1.928
2 Hours	0.311	0.230	0.392	1.365	1.259	1.479	0.366	0.285	0.447	1.442	1.329	1.563
7 Days	0.000	-	-	1.000	-	-	0.000	-	-	1.000	-	-
14 Days	-0.170	-0.241	-0.098	0.844	0.786	0.906	-0.127	-0.198	-0.056	0.881	0.820	0.945
**Accuracy**												
2 in 100 False Negative	1.082	1.032	1.132	2.951	2.807	3.101	1.273	1.223	1.322	3.570	3.396	3.753
5 in 100 False Negative	0.000	-	-	1.000	-	-	0.000	-	-	1.000	-	-
**Consultation Method**												
Online Consultation	0.171	0.097	0.245	1.187	1.102	1.278	0.214	0.140	0.288	1.239	1.151	1.334
Pharmacy Consultation	0.100	0.027	0.173	1.106	1.028	1.189	0.194	0.121	0.268	1.215	1.129	1.307
GP Consultation	0.159	0.085	0.232	1.172	1.089	1.262	0.178	0.104	0.251	1.194	1.110	1.285
Sexual Health Clinic Consultation	0.000	-	-	1.000	-	-	0.000	-	-	1.000	-	-
**Access to Healthcare Professional**												
Phone	-0.065	-0.136	0.005	0.937	0.873	1.005	-0.039	-0.110	0.032	0.962	0.896	1.032
Instant Messenger	-0.006	-0.076	0.063	0.994	0.927	1.065	0.060	-0.010	0.130	1.062	0.990	1.139
Email	0.033	-0.036	0.103	1.034	0.965	1.108	0.060	-0.009	0.130	1.062	0.991	1.139
Face to Face	0.000	-	-	1.000	-	-	0.000	-	-	1.000	-	-
**How you get Antibiotics**												
Post to Home	-0.004	-0.078	0.070	0.996	0.925	1.072	0.027	-0.046	0.101	1.028	0.955	1.106
Post to Collection Point	0.079	0.007	0.151	1.082	1.007	1.163	-0.017	-0.089	0.055	0.983	0.915	1.056
Collect from Pharmacy	0.062	-0.014	0.138	1.064	0.986	1.149	0.083	0.007	0.160	1.087	1.007	1.173
Collect from Sexual Health Clinic	0.000	-	-	1.000	-	-	0.000	-	-	1.000	-	-

Table 3.3 - Age Range Subgroup Analysis Coefficients and Odds Ratios, including Respective 95% Confidence Intervals

	**16-18 *n=415***						**19-21 *n=406***						**22-24 *n=409***					
	**Coeffi-cient**	**Lower 95% CI**	**Upper 95% CI**	**Odds Ratio**	**Lower 95% CI**	**Upper 95% CI**	**Coeffi-cient**	**Lower 95% CI**	**Upper 95% CI**	**Odds Ratio**	**Lower 95% CI**	**Upper 95% CI**	**Coeffi-cient**	**Lower 95% CI**	**Upper 95% CI**	**Odds Ratio**	**Lower 95% CI**	**Upper 95% CI**
**How you Test**																		
Self-Test	0.509	0.395	0.623	1.664	1.485	1.865	0.297	0.182	0.412	1.346	1.200	1.510	0.639	0.523	0.755	1.895	1.687	2.128
Post	0.390	0.275	0.505	1.477	1.316	1.657	0.129	0.013	0.245	1.138	1.013	1.277	0.397	0.281	0.513	1.487	1.324	1.670
Pharmacy	0.239	0.137	0.342	1.270	1.147	1.408	0.021	-0.082	0.124	1.021	0.921	1.132	0.170	0.067	0.273	1.185	1.069	1.314
Education/ Work	-0.218	-0.322	-0.114	0.804	0.725	0.892	-0.215	-0.319	-0.112	0.806	0.727	0.894	-0.160	-0.265	-0.056	0.852	0.768	0.945
GP Practice	0.081	-0.025	0.187	1.085	0.975	1.206	0.035	-0.072	0.141	1.035	0.931	1.152	-0.061	-0.168	0.046	0.941	0.846	1.047
Sexual Health Clinic	0.000	-	-	1.000	-	-	0.000	-	-	1.000	-	-	0.000	-	-	1.000	-	-
**Time to Result**																		
30 Mins	0.567	0.474	0.661	1.763	1.606	1.936	0.636	0.542	0.730	1.888	1.719	2.074	0.572	0.478	0.666	1.772	1.613	1.947
2 Hours	0.282	0.183	0.381	1.326	1.201	1.464	0.437	0.338	0.536	1.548	1.402	1.709	0.295	0.196	0.395	1.344	1.216	1.484
7 Days	0.000	-	-	1.000	-	-	0.000	-	-	1.000	-	-	0.000	-	-	1.000	-	-
14 Days	-0.130	-0.217	-0.044	0.878	0.805	0.957	-0.125	-0.213	-0.038	0.882	0.808	0.963	-0.189	-0.277	-0.102	0.828	0.758	0.903
**Accuracy**																		
2 in 100 False Negative	1.196	1.135	1.257	3.307	3.112	3.514	1.151	1.090	1.212	3.161	2.974	3.361	1.188	1.127	1.250	3.282	3.086	3.489
5 in 100 False Negative	0.000	-	-	1.000	-	-	0.000	-	-	1.000	-	-	0.000	-	-	1.000	-	-
**Consultation Method**																		
Online Consultation	0.171	0.081	0.262	1.187	1.085	1.299	0.182	0.092	0.273	1.200	1.096	1.314	0.226	0.135	0.317	1.253	1.144	1.373
Pharmacy Consultation	0.154	0.065	0.244	1.167	1.067	1.276	0.190	0.100	0.279	1.209	1.105	1.322	0.098	0.007	0.188	1.102	1.007	1.206
GP Consultation	0.246	0.157	0.335	1.279	1.170	1.398	0.133	0.043	0.223	1.142	1.044	1.250	0.125	0.035	0.215	1.133	1.036	1.240
Sexual Health Clinic Consultation	0.000	-	-	1.000	-	-	0.000	-	-	1.000	-	-	0.000	-	-	1.000	-	-

For peer review only

	16-18 *n=415*						19-21 *n=406*						22-24 *n=409*					
	**Coeffi-cient**	**Lower 95% CI**	**Upper 95% CI**	**Odds Ratio**	**Lower 95% CI**	**Upper 95% CI**	**Coeffi-cient**	**Lower 95% CI**	**Upper 95% CI**	**Odds Ratio**	**Lower 95% CI**	**Upper 95% CI**	**Coeffi-cient**	**Lower 95% CI**	**Upper 95% CI**	**Odds Ratio**	**Lower 95% CI**	**Upper 95% CI**
**Access to Healthcare Professional**																		
Phone	0.001	-0.085	0.087	1.001	0.918	1.091	-0.104	-0.190	-0.017	0.902	0.827	0.983	-0.055	-0.142	0.031	0.946	0.868	1.032
Instant Messenger	0.102	0.017	0.188	1.108	1.017	1.207	-0.024	-0.109	0.062	0.977	0.897	1.064	0.001	-0.085	0.087	1.001	0.918	1.091
Email	0.075	-0.009	0.160	1.078	0.991	1.174	0.037	-0.048	0.122	1.038	0.954	1.130	0.029	-0.056	0.115	1.030	0.945	1.122
Face to Face	0.000	-	-	1.000	-	-	0.000	-	-	1.000	-	-	0.000	-	-	1.000	-	-
**How you get Antibiotics**																		
Post to Home	-0.024	-0.114	0.066	0.977	0.893	1.068	0.010	-0.081	0.100	1.010	0.922	1.106	0.047	-0.043	0.137	1.048	0.957	1.147
Post to Collection Point	0.010	-0.078	0.097	1.010	0.925	1.102	0.035	-0.053	0.123	1.036	0.948	1.131	0.046	-0.042	0.135	1.047	0.959	1.144
Collect from Pharmacy	0.039	-0.054	0.132	1.039	0.947	1.141	0.101	0.008	0.195	1.106	1.008	1.215	0.077	-0.017	0.171	1.080	0.983	1.186
Collect from Sexual Health Clinic	0.000	-	-	1.000	-	-	0.000	-	-	1.000	-	-	0.000	-	-	1.000	-	-

For peer review only

Table 3.4 - Relationship Status Subgroup Analysis Coefficients and Odds Ratios, including Respective 95% Confidence Intervals

	Single *n=615*						Sexual Relationship with One Partner *n=512*					
	**Coefficient**	**Lower 95% CI**	**Upper 95% CI**	**Odds Ratio**	**Lower 95% CI**	**Upper 95% CI**	**Coefficient**	**Lower 95% CI**	**Upper 95% CI**	**Odds Ratio**	**Lower 95% CI**	**Upper 95% CI**
**How you Test**												
Self-Test	0.490	0.396	0.584	1.632	1.486	1.792	0.538	0.434	0.641	1.712	1.543	1.899
Post	0.322	0.227	0.416	1.380	1.255	1.516	0.322	0.218	0.425	1.380	1.244	1.530
Pharmacy	0.199	0.115	0.283	1.220	1.122	1.327	0.104	0.011	0.196	1.109	1.011	1.217
Education/ Work	-0.162	-0.247	-0.077	0.851	0.781	0.926	-0.251	-0.344	-0.157	0.778	0.709	0.854
GP Practice	-0.006	-0.093	0.081	0.994	0.911	1.085	0.063	-0.032	0.159	1.066	0.969	1.172
Sexual Health Clinic	0.000	-	-	1.000	-	-	0.000	-	-	1.000	-	-
**Time to Result**												
30 Mins	0.548	0.471	0.625	1.730	1.602	1.867	0.640	0.556	0.724	1.896	1.743	2.062
2 Hours	0.271	0.190	0.352	1.311	1.209	1.422	0.416	0.327	0.505	1.516	1.387	1.657
7 Days	0.000	-	-	1.000	-	-	0.000	-	-	1.000	-	-
14 Days	-0.148	-0.220	-0.077	0.862	0.803	0.926	-0.149	-0.227	-0.071	0.862	0.797	0.932
**Accuracy**												
2 in 100 False Negative	1.186	1.137	1.236	3.275	3.116	3.443	1.196	1.141	1.250	3.305	3.129	3.492
5 in 100 False Negative	0.000	-	-	1.000	-	-	0.000	-	-	1.000	-	-
**Consultation Method**												
Online Consultation	0.145	0.071	0.219	1.156	1.074	1.245	0.251	0.169	0.332	1.285	1.185	1.394
Pharmacy Consultation	0.108	0.035	0.182	1.115	1.036	1.199	0.203	0.123	0.284	1.225	1.130	1.328
GP Consultation	0.150	0.077	0.223	1.162	1.080	1.250	0.173	0.092	0.253	1.189	1.097	1.288
Sexual Health Clinic Consultation	0.000	-	-	1.000	-	-	0.000	-	-	1.000	-	-
**Access to Healthcare Professional**												
Phone	-0.059	-0.130	0.011	0.942	0.878	1.011	-0.077	-0.155	0.000	0.926	0.856	1.000
Instant Messenger	0.025	-0.045	0.095	1.025	0.956	1.099	-0.009	-0.086	0.068	0.991	0.918	1.071
Email	0.066	-0.003	0.136	1.069	0.997	1.145	0.001	-0.076	0.077	1.001	0.927	1.080
Face to Face	0.000	-	-	1.000	-	-	0.000	-	-	1.000	-	-
**How you get Antibiotics**												
Post to Home	-0.044	-0.118	0.030	0.957	0.889	1.030	0.053	-0.028	0.134	1.055	0.973	1.144
Post to Collection Point	-0.032	-0.104	0.040	0.968	0.901	1.041	0.070	-0.009	0.149	1.072	0.991	1.160
Collect from Pharmacy	0.067	-0.009	0.143	1.070	0.991	1.154	0.055	-0.029	0.139	1.056	0.971	1.149
Collect from Sexual Health Clinic	0.000	-	-	1.000	-	-	0.000	-	-	1.000	-	-

For peer review only

Table 3.5 - Previous STI Testing Subgroup Analysis Coefficients and Odds Ratios, including Respective 95% Confidence Interval

	**Previous Test n=393**						**No Previous Test *n=790***					
	**Coefficient**	**Lower 95% CI**	**Upper 95% CI**	**Odds Ratio**	**Lower 95% CI**	**Upper 95% CI**	**Coefficient**	**Lower 95% CI**	**Upper 95% CI**	**Odds Ratio**	**Lower 95% CI**	**Upper 95% CI**
**How you Test**												
Self-Test	0.422	0.305	0.539	1.524	1.356	1.714	0.518	0.434	0.601	1.678	1.544	1.824
Post	0.164	0.047	0.280	1.178	1.048	1.323	0.382	0.298	0.466	1.465	1.347	1.594
Pharmacy	-0.056	-0.160	0.048	0.945	0.852	1.049	0.245	0.170	0.320	1.277	1.185	1.377
Education/ Work	-0.273	-0.377	-0.168	0.761	0.686	0.845	-0.150	-0.225	-0.074	0.861	0.798	0.928
GP Practice	-0.122	-0.229	-0.014	0.885	0.795	0.986	0.097	0.019	0.174	1.101	1.019	1.190
Sexual Health Clinic	0.000	-	-	1.000	-	-	0.000	-	-	1.000	-	-
**Time to Result**												
30 Mins	0.592	0.498	0.687	1.808	1.645	1.988	0.586	0.518	0.654	1.797	1.679	1.923
2 Hours	0.305	0.205	0.406	1.357	1.227	1.500	0.353	0.281	0.425	1.423	1.325	1.529
7 Days	0.000	-	-	1.000	-	-	0.000	-	-	1.000	-	-
14 Days	-0.125	-0.213	-0.037	0.882	0.808	0.963	-0.156	-0.219	-0.093	0.856	0.803	0.912
**Accuracy**												
2 in 100 False Negative	1.099	1.037	1.160	3.000	2.820	3.191	1.248	1.203	1.292	3.482	3.331	3.640
5 in 100 False Negative	0.000	-	-	1.000	-	-	0.000	-	-	1.000	-	-
**Consultation Method**												
Online Consultation	0.161	0.069	0.252	1.174	1.071	1.287	0.205	0.140	0.271	1.228	1.150	1.311
Pharmacy Consultation	0.094	0.003	0.184	1.098	1.003	1.203	0.168	0.103	0.233	1.183	1.108	1.262
GP Consultation	0.106	0.015	0.197	1.112	1.015	1.217	0.195	0.130	0.260	1.215	1.139	1.297
Sexual Health Clinic Consultation	0.000	-	-	1.000	-	-	0.000	-	-	1.000	-	-
**Access to Healthcare Professional**												
Phone	-0.054	-0.142	0.034	0.947	0.868	1.034	-0.048	-0.110	0.015	0.953	0.895	1.015
Instant Messenger	0.044	-0.042	0.131	1.045	0.958	1.140	0.025	-0.037	0.087	1.025	0.963	1.091
Email	0.048	-0.038	0.134	1.049	0.962	1.144	0.053	-0.009	0.114	1.054	0.991	1.121
Face to Face	0.000	-	-	1.000	-	-	0.000	-	-	1.000	-	-
**How you get Antibiotics**												
Post to Home	0.011	-0.081	0.102	1.011	0.922	1.107	0.020	-0.046	0.085	1.020	0.955	1.089
Post to Collection Point	0.034	-0.056	0.123	1.034	0.946	1.131	0.034	-0.029	0.098	1.035	0.971	1.103
Collect from Pharmacy	0.083	-0.012	0.177	1.086	0.988	1.194	0.075	0.008	0.143	1.078	1.008	1.153
Collect from Sexual Health Clinic	0.000	-	-	1.000	-	-	0.000	-	-	1.000	-	-

For peer review only

Table 3.6 - Odds Ratios for all Sub-Groups Included within the Analysis

	Full Dataset	Male	Female	16-18	19-21	22-24	Single	One Partner	Prev Test	No Prev Test
N	**1,230**	**607**	**623**	**415**	**406**	**409**	**615**	**512**	**393**	**790**
	**Odds Ratio**	**Odds Ratio**	**Odds Ratio**	**Odds Ratio**	**Odds Ratio**	**Odds Ratio**	**Odds Ratio**	**Odds Ratio**	**Odds Ratio**	**Odds Ratio**
**How you Test**										
Self-Test	1.618	1.549	1.693	1.664	1.346	1.895	1.632	1.712	1.524	1.678
Post	1.358	1.308	1.411	1.477	1.138	1.487	1.380	1.380	1.178	1.465
Pharmacy	1.155	1.131	1.180	1.270	1.021	1.185	1.220	1.109	0.945	1.277
Education/ Work	0.821	0.919	0.733	0.804	0.806	0.852	0.851	0.778	0.761	0.861
GP Practice	1.019	1.049	0.990	1.085	1.035	0.941	0.994	1.066	0.885	1.101
Sexual Health Clinic	1.000	1.000	1.000	1.000	1.000	1.000	1.000	1.000	1.000	1.000
**Time to Result**										
30 Mins	1.806	1.828	1.786	1.763	1.888	1.772	1.730	1.896	1.808	1.797
2 Hours	1.402	1.365	1.442	1.326	1.548	1.344	1.311	1.516	1.357	1.423
7 Days	1.000	1.000	1.000	1.000	1.000	1.000	1.000	1.000	1.000	1.000
14 Days	0.862	0.844	0.881	0.878	0.882	0.828	0.862	0.862	0.882	0.856
**Accuracy**										
2 in 100 False Negative	3.242	2.951	3.570	3.307	3.161	3.282	3.275	3.305	3.000	3.482
5 in 100 False Negative	1.000	1.000	1.000	1.000	1.000	1.000	1.000	1.000	1.000	1.000
**Consultation Method**										
Online Consultation	1.212	1.187	1.239	1.187	1.200	1.253	1.156	1.285	1.174	1.228
Pharmacy Consultation	1.158	1.106	1.215	1.167	1.209	1.102	1.115	1.225	1.098	1.183
GP Consultation	1.183	1.172	1.194	1.279	1.142	1.133	1.162	1.189	1.112	1.215
Sexual Health Clinic Consultation	1.000	1.000	1.000	1.000	1.000	1.000	1.000	1.000	1.000	1.000
**Access to Healthcare Professional**										
Phone	0.949	0.937	0.962	1.001	0.902	0.946	0.942	0.926	0.947	0.953
Instant Messenger	1.027	0.994	1.062	1.108	0.977	1.001	1.025	0.991	1.045	1.025
Email	1.048	1.034	1.062	1.078	1.038	1.030	1.069	1.001	1.049	1.054
Face to Face	1.000	1.000	1.000	1.000	1.000	1.000	1.000	1.000	1.000	1.000
**How you get Antibiotics**										
Post to Home	1.011	0.996	1.028	0.977	1.010	1.048	0.957	1.055	1.011	1.020
Post to Collection Point	1.031	1.082	0.983	1.010	1.036	1.047	0.968	1.072	1.034	1.035
Collect from Pharmacy	1.075	1.064	1.087	1.039	1.106	1.080	1.070	1.056	1.086	1.078
Collect from Sexual Health Clinic	1.000	1.000	1.000	1.000	1.000	1.000	1.000	1.000	1.000	1.000

For peer review only

STROBE Statement—checklist of items that should be included in reports of observational studies

	Item No	Recommendation
**Title and abstract**	1	(*a*) Indicate the study's design with a commonly used term in the title or the abstract ✔ (page 1)
		(*b*) Provide in the abstract an informative and balanced summary of what was done and what was found ✔ (page 2)
**Introduction**		
Background/rationale	2	Explain the scientific background and rationale for the investigation being reported ✔ (page 4)
Objectives	3	State specific objectives, including any prespecified hypotheses ✔ (page 5)
**Methods**		
Study design	4	Present key elements of study design early in the paper ✔ (page 5)
Setting	5	Describe the setting, locations, and relevant dates, including periods of recruitment, exposure, follow-up, and data collection ✔ (page 7)
Participants	6	(*a*) *Cohort study*—Give the eligibility criteria, and the sources and methods of selection of participants. Describe methods of follow-up ✔ (page 7)<br>*Case-control study*—Give the eligibility criteria, and the sources and methods of case ascertainment and control selection. Give the rationale for the choice of cases and controls<br>*Cross-sectional study*—Give the eligibility criteria, and the sources and methods of selection of participants
		(*b*) *Cohort study*—For matched studies, give matching criteria and number of exposed and unexposed<br>*Case-control study*—For matched studies, give matching criteria and the number of controls per case
Variables	7	Clearly define all outcomes, exposures, predictors, potential confounders, and effect modifiers. Give diagnostic criteria, if applicable ✔ (page 8)
Data sources/ measurement	8*	For each variable of interest, give sources of data and details of methods of assessment (measurement). Describe comparability of assessment methods if there is more than one group ✔ (page 8)
Bias	9	Describe any efforts to address potential sources of bias ✔ (page 8)
Study size	10	Explain how the study size was arrived at ✔ (page 7)
Quantitative variables	11	Explain how quantitative variables were handled in the analyses. If applicable, describe which groupings were chosen and why ✔ (page 7)
Statistical methods	12	(*a*) Describe all statistical methods, including those used to control for confounding ✔ (page 7)
		(*b*) Describe any methods used to examine subgroups and interactions ✔ (page 7-8)
		(*c*) Explain how missing data were addressed
		(*d*) *Cohort study*—If applicable, explain how loss to follow-up was addressed<br>*Case-control study*—If applicable, explain how matching of cases and controls was addressed<br>*Cross-sectional study*—If applicable, describe analytical methods taking account of sampling strategy
		(*e*) Describe any sensitivity analyses
**Results**		
Participants	13*	(a) Report numbers of individuals at each stage of study—eg numbers potentially eligible,

For peer review only

For peer review only - http://bmjopen.bmj.com/site/about/guidelines.xhtml

		examined for eligibility, confirmed eligible, included in the study, completing follow-up, and analysed ✔ (page 8)
		(b) Give reasons for non-participation at each stage
		(c) Consider use of a flow diagram
Descriptive data	14*	(a) Give characteristics of study participants (eg demographic, clinical, social) and information on exposures and potential confounders ✔ (page 8 & Supplementary Information File 2)
		(b) Indicate number of participants with missing data for each variable of interest
		(c) *Cohort study*—Summarise follow-up time (eg, average and total amount)
Outcome data	15*	*Cohort study*—Report numbers of outcome events or summary measures over time ✔ (page 8-9)
		Case-control study—Report numbers in each exposure category, or summary measures of exposure
		Cross-sectional study—Report numbers of outcome events or summary measures
Main results	16	(*a*) Give unadjusted estimates and, if applicable, confounder-adjusted estimates and their precision (eg, 95% confidence interval). Make clear which confounders were adjusted for and why they were included ✔ (page 8-9)
		(*b*) Report category boundaries when continuous variables were categorized
		(*c*) If relevant, consider translating estimates of relative risk into absolute risk for a meaningful time period
Other analyses	17	Report other analyses done—eg analyses of subgroups and interactions, and sensitivity analyses ✔ (page 9)
**Discussion**		
Key results	18	Summarise key results with reference to study objectives ✔ (page 9)
Limitations	19	Discuss limitations of the study, taking into account sources of potential bias or imprecision. Discuss both direction and magnitude of any potential bias ✔ (page 9-10)
Interpretation	20	Give a cautious overall interpretation of results considering objectives, limitations, multiplicity of analyses, results from similar studies, and other relevant evidence ✔ (page 11)
Generalisability	21	Discuss the generalisability (external validity) of the study results ✔ (page 11)
**Other information**		
Funding	22	Give the source of funding and the role of the funders for the present study and, if applicable, for the original study on which the present article is based ✔ (page 12)

*Give information separately for cases and controls in case-control studies and, if applicable, for exposed and unexposed groups in cohort and cross-sectional studies.

For peer review only - http://bmjopen.bmj.com/site/about/guidelines.xhtml

BMJ Open

Young people's preferences for the use of emerging technologies for asymptomatic regular chlamydia testing and management: A discrete choice experiment in England

Journal:	*BMJ Open*
Manuscript ID	bmjopen-2018-023663.R1
Article Type:	Research
Date Submitted by the Author:	20-Aug-2018
Complete List of Authors:	Eaton, Sue; University of Warwick, Warwick Medical School<br>Biggerstaff, Deborah; University of Warwick Warwick Medical School<br>Petrou, Stavros; University of Warwick, Warwick Medical School<br>Osipenko, Leeza; National Institute for Health and Care Excellence, Scientific Advice<br>Gibbs, Jo; University College London, Research Department of Infection and Population Health<br>Estcourt, Claudia; Glasgow Caledonian University,<br>Sadiq, S. Tariq; St George's University of London, Institute for Infection and Immunity Research Institute<br>Szczepura, Ala; Coventry University, Enterprise & Innovation Group; University of Warwick Warwick Medical School
**Primary Subject Heading**:	Public health
Secondary Subject Heading:	Diagnostics, Health policy, Infectious diseases, Sexual health, Patient-centred medicine
Keywords:	sexually transmitted infections, chlamydia trachomatis, patient preferences, discrete choice experiment, online clinical care pathway, digital health

SCHOLARONE™
Manuscripts

Title: Young people's preferences for the use of emerging technologies for asymptomatic regular chlamydia testing and management: A discrete choice experiment in England

Authors:
Dr Sue Eaton, Warwick Medical School, University of Warwick, Coventry, UK.
Dr Deborah Biggerstaff, Warwick Medical School, University of Warwick, Coventry, UK.
Prof Stavros Petrou, Warwick Medical School, University of Warwick, Coventry, UK.
Dr Leeza Osipenko, National Institute for Health and Care Excellence, Scientific Advice, London.
Dr Jo Gibbs, Research Department of Infection & Population Health, University College London, London, UK.
Prof Claudia S Estcourt, Glasgow Caledonian University, Glasgow, UK.
Prof S Tariq Sadiq, Applied Diagnostic Research and Evaluation Unit, Institute for Infection and Immunity, St George's, University of London, London, UK.
Prof Ala Szczepura, Enterprise & Innovation Group, Coventry University, UK.

Corresponding author
Prof Ala Szczepura, BA DPhil (Oxon)
Enterprise & Innovation Group
Coventry University
Coventry CV1 2TT
UK
Email: ala.szczepura@coventry.ac.uk
Mob: +44 (0)7557 425 463

Key words: sexually transmitted infections, chlamydia trachomatis, patient preferences, discrete choice experiment, online clinical care pathway, digital health

For peer review only

ABSTRACT:

Objective: To undertake a comprehensive assessment of the strength of preferences among young people for attributes of emerging technologies for testing and treatment of asymptomatic chlamydia.

Design: Discrete choice experiment (DCE) with sequential mixed methods design. A staged approach to selection of attributes/ levels included two literature reviews, focus groups with young people aged 16-24 years (n = 21), experts' review (n=13), and narrative synthesis. Cognitive testing was undertaken to pilot and adapt the initial questionnaire. Online national panel was used for final DCE survey to maximise generalisability. Analysis of questionnaire responses used multinomial logit models and included validity checks.

Setting: England.

Participants: 1,230 young people aged 16-24 from a national online panel (completion rate 73%).

Outcome Measures: Odds Ratios (ORs) for service attributes in relation to reference levels.

Results: The strongest attribute influencing preferences was chlamydia test accuracy (OR 3.24, 95% CI 3.13–3.36), followed by time to result (OR 1.81, 95% CI 1.71–1.91). Respondents showed a preference for remote chlamydia testing options (self-testing, self-sampling and postal testing) over attendance at a testing location. For accessing treatment following a positive test result, there was a general preference for online (OR 1.21, 95% CI 1.15–1.28) versus traditional GP (OR 1.18, 95% CI 1.12–1.24) or pharmacy (OR 1.15, 95% CI 1.10–1.22) over clinic services. For accessing a healthcare professional and receipt of antibiotics, there was little difference in preferences between options.

Conclusions:
Both test accuracy and very short intervals between testing and results were important factors for young people when deciding whether to undergo a routine test for asymptomatic chlamydia, with test accuracy being more important. These findings should assist technology developers, policy makers, commissioners and service providers to optimise technology adoption in service redesign, although use of an online panel may limit generalisability of findings to other populations.

For peer review only - http://bmjopen.bmj.com/site/about/guidelines.xhtml

Strengths and Limitations of the Study:

- To our knowledge, this is the first large-scale DCE study to examine the preferences of a geographically representative national sample of young people for emerging technologies designed to improve screening for and treatment of asymptomatic chlamydia.
- Robust sequential methods were used to select final DCE attributes and levels, including two literature reviews, focus groups with young people, and review by expert groups.
- An online panel enabled access to the population targeted for screening, including young people with no personal experience of chlamydia testing and treatment.
- A limitation of this work is that the literature reviews inevitably identified more potential attributes than could be included in the DCE.
- The use of an online panel may limit generalisability of findings to the small percentage of this population who do not currently access the internet, and therefore over-represent the acceptability of online care.

For peer review only

INTRODUCTION:

Chlamydia is the most commonly reported sexually transmitted infection (STI) in England, with young people aged 15-24 accounting for 63% of diagnoses in 2016.[1] The estimated annual cost of chlamydia treatment in 2015 was £249.8 million.[2] Undetected infections and re-infections can lead to significant adverse health consequences, such as pelvic inflammatory disease, ectopic pregnancy and infertility, which have an impact on National Health Service (NHS) costs and health-related quality of life.[3] A National Chlamydia Screening Programme (NCSP) was therefore rolled out in England for 16-24 year olds between 2003 and 2008.[4] However, despite recent evidence of a reduction in chlamydia prevalence in England for for 2000 to 2015, concurrent with large-scale population testing[5], and the fact that uncomplicated infection is easy to treat with oral antibiotics, uptake of screening for chlamydia remains low.[6] Worryingly, there has also been a decline over the last four years in the number of local authorities achieving the public health outcomes framework indicator of 2,300 diagnoses per 100,000 population.[1] Barriers identified for young people accessing STI testing and treatment services include tangible service attributes, such as location of service, and personal or behavioural factors such as the stigma associated with attendance, embarrassment, fear of being recognised and privacy concerns.[7-9]

A range of options are currently available for young people to access asymptomatic chlamydia testing and treatment services. For testing, options include attending a genito-urinary medicine (GUM) clinic, testing via primary care, e.g. pharmacies/general practitioners (GPs), or via internet testing services where a number of websites offer free self-sampling kits online with samples sent to laboratories for analysis and results communicated directly to patients. For individuals who screen positive, options for accessing antibiotic treatment include attending a GUM/sexual health clinic, GP practice or pharmacy. Even though a national screening programme exists in England, large geographical variations exist in testing coverage, from 16% of young people tested for chlamydia in the West Midlands region to 27% in London, and in the proportion of positive cases treated.[1 10]

For many health services, digital technology is now widely regarded by policy makers as one approach to improve access and reduce costs.[11] For chlamydia testing and treatment, a range of digital options are available, and more technologies are likely to enter the market within the next 3-5 years.[12] These include point-of-care tests[13], online postal self-sampling, eSexual health clinics[14], apps and non-face-to-face consultation methods[15 16], and self-tests networked through mobile phones[17]. Such innovations provide an opportunity to redesign current chlamydia screening services with the aim of improving testing and treatment uptake. However, such service redesign should be based on a sound understanding of the preferences of young people as service-users for specific attributes of such services.

Research specifically measuring young people's preferences nationally for chlamydia testing and treatment service options is lacking. Even for STI testing services generally, relatively few studies have used a discrete choice experiment (DCE) design to assess preferences in the UK population.[18-21] In comparison with other preference elicitation methods, a DCE can quantify the relative importance of different attributes that characterise a new or existing

For peer review only - http://bmjopen.bmj.com/site/about/guidelines.xhtml

product and/or service, identifying which attributes people prioritise and which they may be willing to trade with the view to maximising their utility.[22] A DCE requires respondents to choose between competing scenarios, e.g. service options, described in terms of a particular attribute (e.g. time to test result) and a range of levels (e.g. 30min to 14 days) and to compare these against an alternative scenario. DCE studies are very useful because they allow a direct assessment of relative preferences for various existing and hypothetical new service configurations or treatment approaches.[23]

The aim of the present study was to undertake a comprehensive assessment of the preferences of young people. targeted by the National Chlamydia Screening Programme, for emerging technology options for testing and treatment in the context of a "check-up test" where remote care could be medically appropriate.[14] To our knowledge, no previous study has attempted to disentangle strength of preference for attributes associated with new and emerging options for chlamydia screening (such as self-testing) or treatment (such as online prescription). Because STI services are "open access" and not subject to gatekeeping by referral from a clinician, the impact of such disruptive innovations will be directly dependent on population preferences. Early insight into attributes that could influence whether individuals are more likely to use a new testing or treatment pathway should therefore be helpful in informing product development and pathway redesign for future chlamydia screening service models.[24 25]

METHODS:

This study was conducted using an exploratory sequential mixed methods design, adopting recommended stages for undertaking a DCE[26], as shown in Figure 1. In selecting methods to design the questionnaire and conduct the DCE, reference was made to the International Society for Pharmacoeconomics and Outcomes Research good practice checklist for conjoint analysis.[27]

Patient and public involvement: Young people (with and without experience of STI services) participated in focus groups to inform priorities included in the DCE; in cognitive testing to finalise questionnaire design; and in questionnaire completion to identify preferences, as described in the Acknowledgements.

Selection of Attributes and Levels

The attributes and associated levels were determined using a four-stage approach.[28]

Literature Reviews

Two literature reviews were undertaken to produce a comprehensive list of potential themes and factors that might influence young people's choices. These included: (i) a systematic review of the use of stated preference studies for STI testing and treatment (PROSPERO Reg: CRD42014014862); and (ii) a scoping review of other research exploring preferences and acceptability of STI testing and treatment services.

Focus Groups

For peer review only - http://bmjopen.bmj.com/site/about/guidelines.xhtml

Focus groups were run with young people aged 16-24 years (4 groups, 3-7 per group; total n=21) to identify which themes and factors young people consider important when choosing to test for STIs. Convenience sampling was used to identify participants. Focus group topic guides incorporated typical vignettes of situations individuals might encounter. Sessions were recorded, transcribed and thematic analysis was performed to produce a list of potential attributes and levels.[29]

Expert Groups

Four expert groups were convened (n=13), including a range of professionals with expert knowledge of the service and technology context. Expert groups were asked to review the focus group findings in terms of clinical feasibility and practicality.[26]

Narrative Synthesis

Narrative synthesis[30] enabled outputs from the three prior stages to be synthesised for each potential attribute. This approach was adopted as it offered a clear approach to synthesis based on the following stages: (i) identification of a checklist of properties against which attributes can be considered; (ii) tabulation against the checklist; and (iii) conceptual mapping and triangulation against the checklist. The final synthesis highlighted a tension between young people's desire to be tested for a wide range of STIs irrespective of risk, versus clinical guidelines for selective testing of STIs based on population group prevalence and risk. The range of STIs presented in the DCE was consequently limited to Chlamydia. Because focus group findings indicated difficulty in understanding several dimensions of test performance, test accuracy was expressed in terms of the likelihood of a false negative result.

Questionnaire Design and Piloting

A generic pairwise choice with opt out question was selected for the questionnaire design. Respondents were presented with a series of choice sets for which there were 3 responses: 'option A', 'option B' or 'I would not test'. A sample choice set is illustrated in Figure 2. The questionnaire adopted a main effects design using full profiles (all attributes included in the study). Whilst some DCEs do include an attribute on economic costs, DCEs exploring preferences for STI testing services in England have generally excluded cost since the NHS provides treatment 'free at the point of delivery'[20 21] A cost attribute was therefore not included.

The questionnaire was developed using SAS 9.4 software to ensure that the design was D-efficient.[27] A full factorial design was ruled out in favour of a fractional factorial design because a full factorial design would have contained 3,072 possible alternatives, which would have been unmanageable in practice for individuals to complete or for a blocked questionnaire format to handle[31]. The smallest 100% efficient design that could be created included 48 choice sets. These were blocked (halved) into two questionnaires each with 24 choice sets using SAS JMP Pro 9.2.0. Choice set 1 was repeated as choice set 25 in each questionnaire to provide an internal validity check.

Three rounds of cognitive testing (n=9) were undertaken to check respondents' comprehension of information when making choices. Cognitive testing, undertaken based on two questionnaires of 24 sets, confirmed that a study based on two such questionnaires

For peer review only

was acceptable to participants. Some modifications were made to levels where reasons for choice selection demonstrated that one level (e.g. 8 in 100 false negative) dominated the reason for selection. Cognitive testing also identified that implausible combinations (e.g. a postal test providing a result in two hours) impacted on completion of the task, whilst unlikely (but feasible) combinations did not. Only implausible combinations were therefore excluded from the choice sets. The D-Efficiency of the design[27] calculated by SAS JMP Pro 9.2.0, prior to the removal of implausible combinations, was 98.06. The final combination selected for the questionnaire was the one which created no duplicate choices and which provided an equal balance of the number of choice sets containing overlap between questionnaires.

Respondents were asked to provide socio-demographic information, including their age, gender, ethnicity, region of residence, sexual preference, relationship status and whether they had previously been tested for an STI. An introduction to the DCE questionnaire provided background information and an explanation of the attributes and levels. The introduction was included in the cognitive testing rounds to check comprehension within the target age range. As a result, the text was modified and diagrams added to illustrate the chlamydia testing pathways. The final phase of testing demonstrated that the number of choice sets was acceptable to respondents. The use of an online panel also provided completion time data to support the internal validity checks, and enabled an accurate record of time taken to complete the survey.

Final Attributes and Levels Selected

The six attributes and 24 levels selected for inclusion in the final DCE questionnaire are shown in table 1. The detailed information used to explain the attributes and levels is included in Supplementary Information File 1 (pages 3-7).

Participants and Recruitment Procedure

Participants were drawn from the general population rather than from health care settings, thereby providing access to young people aged 16-24 years who had not previously been tested for an STI. An online national panel (YouthSight)[32] was used to maximise geographical reach and generalisability. Ex-ante sub-group analysis was planned by three age bands (16-18, 19-21, 22-24) and by gender (male, female). Where sub-group analysis is planned, it is recommended that there should be a minimum of 200 respondents in each sub-group, so a sample size of 1,200 was required in our study, 600 per questionnaire.[33] Consent was obtained online prior to questionnaire completion. Participants were offered a small reimbursement of one point (equivalent to £1) for completion of a survey of up to 20 minutes in length. Points could be exchanged for shopping vouchers.

Statistical Analyses

The multinomial logit (MNL) model developed by McFadden was used for analysing responses; this is recognised as the convention for three-response choice set studies ('option A', 'option B' or 'I would not test').[26 34] Analysis utilised STATA13 SE with the method and code outlined by Ryan *et al*[23] Analyses presented odds ratios (ORs), 95% confidence interval (CIs) and coefficients for each attribute level. Variables within the model were all treated as categorical variables for the analysis. Attribute levels were specified using dummy coding, the preferred form of coding where ORs are to be calculated. Within

For peer review only

the model, the levels that were dropped to form the reference levels reflected those aligned to a 'typical' sexual health clinic pathway (summarised in table 2). To test the internal validity of questionnaire responses, analyses compared full results against: (i) removal of responses where participants did not answer the repeated choice set consistently; (ii) removal of any respondents who took less than the minimum time (five minutes) observed in cognitive testing to complete the questionnaire; and (iii) removal of responses containing the opt out question data. Further tests for internal consistency and rationality were not included, since excluding responses on this basis may be viewed as an inappropriate imposition of rationality.[35]

Demographic characteristics (gender, age and ethnicity) of respondents were compared with national Census data.[36] The influence of patient level characteristics (age, sex and STI testing history (yes/no)) on the likelihood of not choosing to test was examined. In addition to *ex-ante* planned sub-group analyses, if sufficient responses were received analysis was also planned to compare: (i) respondents who had, or had not, previously tested for an STI, and (ii) those who indicated their relationship status as 'single' versus those in a sexual relationship with one person.

Trade-off between accuracy and time to result was examined by considering the probability of uptake for tests with characteristics at the opposite ends of the spectrum i.e. 'lower accuracy (5% false negatives), faster time to result (30 min)' and 'higher accuracy (2% false negatives), longer time to result (14 days)'.

RESULTS:

In total, 1,230 fully completed questionnaires were received, the platform analytics showed that 460 people had opened the questionnaire but did not complete it, providing a completion rate of 73%.. No further information was available on the demographics of the 460 non-responders nor any information on the point at which they chose to exit the survey, so these people could not be included in our analyses. Time to complete the 25 choice sets ranged from one minute 19 seconds to 30 minutes 19 seconds with a median time to completion of seven minutes 51 seconds. Demographic characteristics of respondents are summarised in the Supplementary Information File 2 (Part 1). Comparing the DCE sample to the characteristics of 16-24 year olds in England indicates that gender, age and ethnicity are broadly in line with national population demographics, with a slight under-representation of the 16-18 year age group. In terms of the geographical residence of participants, the sample was broadly in line with the geographical distribution of 16-24 year olds identified in the ONS Mid-Year Population Estimates 2015[37], with the exception of a lower proportion of respondents from the East of England.

Internal validity checks showed that, in comparison to the full dataset, all checks yielded very similar ORs with no change in the order of the strength of preference for a level within each attribute. Results of the internal validity checks are included in the Supplementary Information File 2 (Part 2).

OR values for the full dataset and subgroups analysed are presented in Table 3 with reference levels (1.00). Analysis of the trade-off between accuracy and time to result is presented in Table 4. More detailed DCE results, including 95% CIs as well as OR values plus

For peer review only - http://bmjopen.bmj.com/site/about/guidelines.xhtml

coefficients for each attribute level for all sub-group analysis are presented in Supplementary Information File 2 (Part 3).

In Table 3, looking across all attributes and levels for the whole population, participants expressed the strongest preference for attribute 1 - accuracy of test result (OR 3.242, 95% CI 3.130–3.359). There are also differences in the strength of preference between males and females for this attribute (OR 2.951, 95% CI 2.807–3.101 and 3.570, 95% CI 3.396–3.753, respectively), and between those who had previously tested or not tested (OR 3.000, 95% CI 2.820–3.191 and 3.482, 95% CI 3.331–3.640, respectively). Time to result was the attribute showing the next strongest preference across all subgroups (OR 1.806, 95% CI 1.711–1.906). These results are consistent with the logical expectation that people will prefer higher accuracy and a shorter waiting time. Looking specifically at the trade-off between accuracy and time to result, Table 4 indicates that participants are willing to wait noticeably longer in order to have a test result with a lower chance of a false negative result.

When considering how to test, all subgroups demonstrated a preference for self-testing (OR 1.618, 95% CI 1.514–1.729) over attendance at a sexual health clinic. Testing via an outreach service in an educational/ work setting was found to be the least preferred option (OR 0.821, 95% CI 0.773–0.872). Respondents showed a consistent strength of preference for those options that do not involve direct contact with healthcare professionals (self-testing and postal self-sampling), compared with those that do. For consultation and treatment following a positive test result, there was a preference for non-sexual health clinic pathways with online consultation to access treatment (OR 1.212, 95% CI 1.150–1.277), treatment via general practice (OR 1.183, 95% CI 1.123–1.246) and treatment via pharmacy (OR 1.158, 95% CI 1.100–1.220) preferred in the full dataset. At subgroup level (age, gender, previous testing history and relationship status), more variation was found in the preference order for this attribute, with the exception of the sexual health clinic which was consistently the least preferred option across all subgroups (see table 3 and Supplementary Information File 2, Part 3). Finally, the full dataset shows that, if someone wants to access a healthcare professional, there was no statistically significant preference for instant messenger or email access compared with accessing the professional face-to-face. Telephone access to a healthcare professional was the least preferred access option (OR 0.949, 95% CI 0.903–0.998), and the only statistically significant result when compared with face-to-face access (the reference level). There was, similarly, no statistically significant difference in preferences for how young people might access antibiotics apart from a slight preference for the pharmacy versus sexual health clinic (OR 1.075, 95% CI 1.018–1.134).

DISCUSSION:

Our findings indicate that, based on the levels included in the DCE questionnaire, young people are willing to wait in order to have a chlamydia screening test result with a lower chance of a false negative result. The conclusion for new test developers is that time to result is less important than accuracy, and that test users are unlikely to prefer a point-of-care or self-screening test with lower accuracy than the tests currently available to them. There was a strong preference for remote access to testing, consultation, and antibiotic prescriptions, although for accessing a health professional there was no preference

For peer review only - http://bmjopen.bmj.com/site/about/guidelines.xhtml

between online and face-to-face methods. This suggests a remote online pathway is acceptable to young people, *as long as* test performance remains equivalent.

In the various hypothetical situations presented, respondents showed a preference for chlamydia self-testing, self-sampling and postal testing over attendance at a testing location. For accessing treatment, a general preference was exhibited for online versus traditional GP or pharmacy services over clinic services. For receipt of antibiotics, there was little difference in preferences. We were also able to identify which attributes people may be willing to trade to maximise their utility. Looking at the trade-off between accuracy and time to result, we found that young people are willing to wait noticeably longer in order to have a test result with a lower chance of a false negative result, reinforcing the need for test equivalence.

The strengths of this DCE study include, firstly, the robust methods employed to select attributes and levels, which aligned with the recommendations of Coast *et al*, ensuring attributes are "manipulable in policy".[38] Secondly, the fact that participants were drawn from the general population targeted by the National Chlamydia Screening Programme, rather than from healthcare settings. This enabled us to access a demographically and geographically representative national sample of young people, including those who have had no previous contact with STI services. Finally, the use of an online panel enabled large scale data to be collected at a reasonable cost and allowed validity checks that would not otherwise have been possible with postal questionnaire responses. The large sample size also allows comparison between several subgroups to explore differences based on age, gender, previous testing history and relationship category.

However, the study does have a few limitations. The first relates to the selection of attributes and levels. Whilst the selection process employed was very rigorous, this cannot detract from the fact that further attributes were identified which might impact on individuals' choices. To mitigate against this, where such an attribute was excluded, information was provided in the survey background section to minimise respondents forming their own views on the impact of this attribute on the pathway.

Use of an online panel also provided accurate records of the time taken to complete the survey, and permitted additional validity checks which would not otherwise have been possible with a written questionnaire. On the other hand, use of an online panel did exclude populations who do not have access to the internet, thereby potentially over-representing the acceptability of online care. However, given the extremely high proportion (97%) of 15-24 year-olds accessing the internet daily via a mobile device[39] and owning a smartphone[40], it is evident that the vast majority of the target population could access online care pathways if they choose to do so. The question of whether these young people have the degree of digital and health literacy needed for online testing and treatment was not explored in this study.

Behavioural factors, such as embarrassment[7], stigma[41] and privacy and anonymity concerns[42 43] are known to influence uptake of sexual health services, as well as structural factors such as convenience and perceived barriers to access[8]; many of these may be lower for a non-face-to-face service. Balanced against this, access to online testing and treatment

For peer review only - http://bmjopen.bmj.com/site/about/guidelines.xhtml

is valued less strongly than attributes such as test accuracy and time to result. In individual cases a young person's preference for remote versus face-to-face testing and treatment might differ.

Finally, it is difficult to compare the present results to other published DCEs because of key differences in perspective. Miners *et al* (sample n=3,358)[20] and Llewellyn *et al* (sample n=233)[21] both only focussed on preferences within existing traditional service delivery models, and did not incorporate any hypothetical future scenarios (e.g. point-of-care testing, self-testing or treatment via eHealth/mHealth solutions). Two other DCE studies, which did consider self-sampling at home for chlamydia screening[18 19], with sample sizes 174 and 126 respectively, both described sending the sample to a laboratory for analysis rather than a self-test. One of these studies[19] did identify a stronger preference for attendance at a family planning clinic, rather than self-sampling, but since participants were recruited from the waiting room of such a clinic they cannot be considered representative of the general population targeted by the NCSP. A fifth study, which examined preferences for point-of-care testing, only surveyed clinicians undertaking STI testing (sample n=218), thus excluding the population actually targeted for chlamydia screening.[44] The only stated preference study that has considered patients' preferences for STI self-testing focussed on HIV (sample n=365).[45] The authors reported results in line with our findings, with respondents exhibiting a preference for tests which are accurate, timely and private/ anonymous. However, this study was undertaken in 2002 prior to smartphones and at a time when self-testing for HIV was still under development. Importantly for screening tests, only one DCE study to date[21] has sought to include non-service users (i.e. populations with no experience of STI testing). The authors identified a preference for testing for all STIs, in settings with healthcare professionals with specialist knowledge present, and for receipt of negative as well as positive results. This study used a convenience sample of 233 students from two universities which was unlikely to be geographically or socio-economically representative. All other studies have drawn their DCE samples from people who were either current service users, or attendees for other, linked services. Use of such samples represents a significant short-coming when considering the introduction of new technologies for asymptomatic chlamydia testing and management. Most sample sizes were also smaller than in the present study.

A number of questions remain which our research does not address. Firstly, recognising that the range of STIs included is an important consideration for young people in choosing to test[20 21], further research is required to understand this better in the context of potential new screening pathways which incorporate other STIs (e.g. gonorrhoea). Secondly, our study highlights a number of methodological considerations where there is an absence of consensus that may warrant further exploration to improve consistency. These include the number of choice sets to include in a DCE and the use of repeated choice sets as an internal validity measure. Finally, given that cost was not included as an attribute in this study, it is not possible to provide an indication of willingness to pay, or the monetary benefits of potential service changes, from this DCE.[46]

The present study is, to our knowledge, the first to present a large scale, quantitative analysis of young people's preferences for attributes of potential new pathways to deliver testing and treatment of asymptomatic chlamydia, based on a nationally representative population. The DCE methodology applied also produced a measure of the relative strength

For peer review only

of preference between different attributes and levels, and potential trade-offs. This can provide useful evidence to technology developers, policy makers, commissioners and service providers. In particular, it provides a first insight into preferences for the type of technologies currently under development, and those which might be available for use in the near future, compared to the features of existing products and services. This can indicate how young people may respond to changes in pathways and to the introduction of new technologies.

Within the context of current UK sexual health policy and commissioning of sexual health services[12], this DCE provides supportive evidence for the policy direction of remote chlamydia testing and treatment. However, whilst young people overall expressed a stronger preference for attributes such as self-test, online consultation etc, a small proportion still preferred existing pathways. This suggests that, in order to maximise benefit, face-to-face services should continue to be available in addition to any online screening and treatment service. This will ensure that services are inclusive and accessible irrespective of digital/ health literacy, while recognising that people's needs and preferences may change depending on their personal circumstances.

Acknowledgements

We thank Dr Joshua Pink, National Institute for Health and Care Excellence (NICE), Manchester, UK for providing specialist advice on DCE design and analysis. We would also like to thank academics in the Studies in Adolescent Sexual Health (SASH) Group at Coventry University who provided advice on how to access the young people who participated in focus groups; and YouthSight for their expertise in converting the questionnaire into an online format. Finally, the authors wish to thank all the young people who took the time to participate in the focus groups, cognitive testing and complete a detailed questionnaire describing their preferences, and the experts who contributed their views in the expert groups.

Contributors:

SE conceived and undertook the study as part of her PhD research. AS was lead supervisor and takes responsibility for the integrity of the data and the accuracy of the data analysis. SP, LO and AS oversaw the DCE design and analysis; DB guided the qualitative research. CSE and JG provided clinical input into the development of the DCE materials and assisted with interpretation of findings. SE, AS, SP, LO and DB wrote the first draft of the article. STS (Principal Investigator), AS and CSE were applicants on the eSTI2 Consortium grant, wrote the initial protocol and obtained funding. All authors contributed to and agreed the final version of the manuscript.

Funding:

The Electronic Self-Testing Instruments for Sexually Transmitted Infection Control (eSTI^2) Consortium (which funded SE's studentship and also AS, JG, CSE and STS) was funded under the UKCRC Translational Infection Research (TIR) Initiative supported by the Medical Research Council (Grant Number G0901608) with contributions to the Grant from the Biotechnology and Biological Sciences Research Council, the National Institute for Health Research on behalf of the Department of Health, the Chief Scientist Office of the Scottish Government Health Directorates and the Wellcome Trust.

For peer review only

The funding body had no role in the design of the study, analyses, interpretation, or decision to submit the manuscript for publication.

Competing Interests:
None Declared

Ethics Approval
Ethical approval was granted by the Biomedical & Scientific Research Ethics Committee at the University of Warwick (ref: REGO-2015-1647).

Data Sharing Statement:
Requests for access to data should be addressed to the corresponding author

For peer review only

References

1. Public Health England. Sexually Transmitted Infections and Chlamydia Screening in England, 2016. 2017. https://assets.publishing.service.gov.uk/government/uploads/system/uploads/attachment_data/file/617025/Health_Protection_Report_STIs_NCSP_2017.pdf
2. Development Economics. Unprotected Nation - The Financial and Economic Impacts of Restricted Contraceptive and Sexual Health Services. 2013. https://www.fpa.org.uk/sites/default/files/unprotected-nation-sexual-health-full-report.pdf.
3. Public Health England. Opportunistic Chlamydia Screening of Young Adults in England: An Evidence Summary. 2014. https://assets.publishing.service.gov.uk/government/uploads/system/uploads/attachment_data/file/497371/Opportunistic_Chlamydia_Screening_Evidence_Summary_April_2014.pdf.
4. Public Health England. Guidance: NCSP: programme overview. 2013. https://www.gov.uk/government/publications/ncsp-programme-overview/ncsp-programme-overview.
5. Lewis J, White PJ. Changes in chlamydia prevalence and duration of infection estimated from testing and diagnosis rates in England: a model-based analysis using surveillance data, 2000-15. Lancet Public Health 2018;**3**(6):e271-e78.
6. Public Health England. Health protection report, volume 10, issue 22. 2016. https://www.gov.uk/government/publications/health-protection-report-volume-10-2016/hpr-volume-10-issue-22-news-8-july
7. Booth A R, Harris P R, Goyder E, et al. Beliefs about chlamydia testing amongst young people living in relatively deprived areas. J Public Helath (Oxf) 2013;**35**:213-22.
8. Friedman AL, Bloodgood B. Exploring the feasibility of alternative STD-testing venues and results delivery channels for a national screening campaign. Health Promot Pract 2013;**14**(1):96-104.
9. Balfe M, Brugha R. What prompts young adults in Ireland to attend health services for STI testing? BMC Public Health 2009;**9**:311.
10. NCSP. NCSP Scorecard 2011-12. 2012. http://www.chlamydiascreening.nhs.uk/ps/data.asp.
11. National Information Board & Department of Health. Personalised Health and Care 2020 - Using Data and Technology to Transform Outcomes for Patients and Citizens, A Framework for Action. 2014. https://www.gov.uk/government/uploads/system/uploads/attachment_data/file/384650/NIB_Report.pdf.
12. Eaton S, Osipenko L, Petrou S, et al. National Telemedicine and Telehealth Policy Context in England and Introduction of Digital Innovations for Detection and Treatment of Sexually Transmitted Infections. SM J Clin Med 2017;**3**(1).
13. Harding-Esch EM, Nori AV, Hegazi A, et al. Impact of deploying multiple point-of-care tests with a 'sample first' approach on a sexual health clinical care pathway. A service evaluation. Sex Transm Infect 2017;**93**(6):424-29.
14. Estcourt C GJ, Sutcliffe L, Gkatzidou V, Tickle L, Hone K, Aicken C, Lowndes C, Harding-Esch E, Sue Eaton S, Oakeshott P, Szczepura A, Ashcroft R, Copas A, Nettleship A, Sadiq SJ, Sonnenberg P,. The eSexual Health Clinic System for Sexually Transmitted

For peer review only - http://bmjopen.bmj.com/site/about/guidelines.xhtml

Infection Management, Prevention and Control: Exploratory studies demonstrating safety, feasibility and public health utility. The Lancet Public Health 2017.
15. Lloyds Pharmacy. Online Doctor. 2018. https://onlinedoctor.lloydspharmacy.com.
16. City Health Care Partnership CIC. Sexual & Reproductive Healthcare Services. 2018. www.sexualhealthvirtualclinic.co.uk.
17. UK Research and Innovation. Enabling and translating advances in diagnostic and communication technologies to reduce the burden of STIs. 2018. http://gtr.ukri.org/projects?ref=G0901608.
18. Ryan M, Watson V. Comparing welfare estimates from payment card contingent valuation and discrete choice experiments. Health Econ 2009;**18**(4):389-401.
19. Watson V, Ryan M, Watson E. Valuing experience factors in the provision of Chlamydia screening: an application to women attending the family planning clinic. Value Health 2009;**12**(4):621-3.
20. Miners A, Llewellyn C, Pollard A, et al. Assessing user preferences for sexually transmitted infection testing services: a discrete choice experiment. Sex Transm Infect 2012;**88**(7):510-6.
21. Llewellyn CD, Sakal C, Lagarde M, et al. Testing for sexually transmitted infections among students: a discrete choice experiment of service preferences. BMJ open 2013;**3**(10):e003240.
22. Lancsar E, Louviere J. Conducting discrete choice experiments to inform healthcare decision making: a user's guide. Pharmacoeconomics 2008;**26**(8):661-77.
23. Ryan M, Watson V, Krucien N, et al. Using Discrete Choice Experiments in Health Economics: Theoretical and Practical Issues. University of Aberdeen, UK: Health Economics Research Unit, 2014.
24. Hall J, Viney R, Haas M, et al. Using stated preference discrete choice modeling to evaluate health care programs. Journal of Business Research 2004;**57**:1026-32.
25. Ryan M. Discrete choice experiments in health care. BMJ 2004;**328**(7436):360-1.
26. Ryan M, Gerard K, Amaya-Amaya ME. *Using Discrete Choice Experiments to Value Health and Health Care.* Dordrecht, The Netherlands: Springer, 2008.
27. Bridges JF, Hauber AB, Marshall D, et al. Conjoint analysis applications in health--a checklist: a report of the ISPOR Good Research Practices for Conjoint Analysis Task Force. Value Health 2011;**14**(4):403-13.
28. Eaton S. Integrating Novel Digital Technology for the Testing & Treatment of Chlamydia into Mainstream Sexual Health Services in England. University of Warwick, 2017.
29. Silverman D. *Interpreting Qualitative Data, London, Sage Publications Ltd.* London: Sage Publications Ltd., 2014.
30. Pope C, Mays N, J. P. *Synthesising Qualitative and Quantitative Health Evidence A Guide to Methods*. Maidenhead, England: Open University Press, 2007.
31. Vanniyasingam T, Daly C, Jin X, et al. Investigating the impact of design characteristics on statistical efficiency within discrete choice experiments: A systematic survey. Contemp Clin Trials Commun 2018;**10**:17-28.
32. Youthsight. YouthSight Panel Services. 2018. http://www.youthsight.com/expertise/panel-services/.
33. Orme B. *Getting Started with Conjoint Analysis: Strategies for Product Design and Pricing Research, Madison, Wisconsin, Research Publishers LLC.* Madison, USA: Research Publishers LLC, 2010.

For peer review only - http://bmjopen.bmj.com/site/about/guidelines.xhtml

34. Louviere J, Hensher DA, Swait JD. *Stated Choice Methods Analysis and Application.* Cambridge, England.: Cambridge University Press, 2003.
35. Clark MD, Determann D, Petrou S, et al. Discrete choice experiments in health economics: a review of the literature. Pharmacoeconomics 2014;**32**(9):883-902.
36. Office for National Statistics. 2011 Census, Key Statistics for Local Authorities in England and Wales . 2012. http://webarchive.nationalarchives.gov.uk/20160107112030/http://www.ons.gov.uk/ons/publications/re-reference-tables.html?edition=tcm%3A77-286262.
37. Office for National Statistics. Population Estimates for UK, England and Wales, Scotland and Northern Ireland: mid-2015. . 2016. https://www.ons.gov.uk/peoplepopulationandcommunity/populationandmigration/populationestimates/bulletins/annualmidyearpopulationestimates/mid2015.
38. Coast J, Al-Janabi H, Sutton EJ, et al. Using qualitative methods for attribute development for discrete choice experiments: issues and recommendations. Health Econ 2012;**21**(6):730-41.
39. Office for National Statistics. Internet access – households and individuals: 2016. 2016. https://www.ons.gov.uk/peoplepopulationandcommunity/householdcharacteristics/homeinternetandsocialmediausage/bulletins/internetaccesshouseholdsandindividuals/2016.
40. Ipsos Connect. Technology Tracker. Quarterly Release: Q2 2016. 2016. https://www.ipsos.com/sites/default/files/2016-10/Ipsos_Tech_Tracker_Q2_2016.pdf.
41. Balfe M, R. B. What concerns do young adults in Ireland have about attending health services for STD testing? Deviant Behav 2011;**32**:320-50.
42. Baraitser P, Brown KC, Gleisner Z, et al. 'Do it yourself' sexual health care: the user experience. Sex Health 2011;**8**(1):23-9.
43. Lorimer K, McDaid L. Young men's views toward the barriers and facilitators of Internet-based Chlamydia trachomatis screening: qualitative study. Journal of medical Internet research 2013;**15**(12):e265.
44. Hsieh YH, Gaydos CA, Hogan MT, et al. What qualities are most important to making a point of care test desirable for clinicians and others offering sexually transmitted infection testing? PloS one 2011;**6**(4):e19263.
45. Phillips KA, Maddala T, Johnson FR. Measuring preferences for health care interventions using conjoint analysis: an application to HIV testing. Health Serv Res 2002;**37**(6):1681-705.
46. de Bekker-Grob EW, Ryan M, Gerard K. Discrete choice experiments in health economics: a review of the literature. Health Econ 2012;**21**(2):145-72.

For peer review only

FIGURE LEGENDS

Figure 1 – Stages in Conducting a Discrete Choice Experiment

Figure 2 – Sample Choice Set from the Discrete Choice Experiment

For peer review only

Table 1 – Summary of Attributes and Levels Included in the Study
Full descriptions provided in Supplementary Information File 1 (pages 3-7).

Attribute / Level	A1. How you Test	A2. Time to Result	A3. Accuracy	A4. Consultation Method	A5. Access to HCP	A6. How you get Antibiotics
**L1.**	Self-test	30 mins	2 in 100 (False Negative)	Online Consultation	Phone	Post to Home
**L2.**	Self-Sample & Post	2 Hours	5 in 100 (False Negative)	Pharmacy	IM	Post to Collection Point
**L3.**	Self- Sample & Pharmacy	7 Days		GP	Email	Collect from Pharmacy
**L4.**	Self-Sample & Education/ Work	14 Days		Sexual Health Clinic	Face to Face	Collect from Sexual Health Clinic
**L5.**	GP Practice – Sample taken by healthcare professional					
**L6.**	Sexual Health Clinic – Sample taken by healthcare professional					

For peer review only - http://bmjopen.bmj.com/site/about/guidelines.xhtml

Table 2 – Dummy Coding Reference Level

Attribute	Reference Level
How you test	Sexual Health Clinic
Time to result	7 Days
Accuracy	5 in 100 (False Negative)
Consultation method	Sexual Health Clinic
Access to HCP	Face to Face
How you get antibiotics	Collect from Sexual Health Clinic

For peer review only - http://bmjopen.bmj.com/site/about/guidelines.xhtml

Table 3 – Overview Odds Ratios for all Sub-Groups Included within the Analysis.

For further information regarding the explanation of attributes and levels to young people in the questionnaire please see ***Supplementary File 1****.*
Odds Ratio values not statistically significant are shaded blue.
95% Confidence Intervals provided in ***Supplementary File 2****.*

	Full Dataset	Male	Female	16-18	19-21	22-24	Single	One Partner	Prev Test	No Prev Test
**Attribute & Levels**	**1,230**	**607**	**623**	**415**	**406**	**409**	**615**	**512**	**393**	**790**
	**Odds Ratio**	**Odds Ratio**	**Odds Ratio**	**Odds Ratio**	**Odds Ratio**	**Odds Ratio**	**Odds Ratio**	**Odds Ratio**	**Odds Ratio**	**Odds Ratio**
**1. How you Test**										
Self-Test	1.618	1.549	1.693	1.664	1.346	1.895	1.632	1.712	1.524	1.678
Self-Sample and post to a laboratory for analysis	1.358	1.308	1.411	1.477	1.138	1.487	1.380	1.380	1.178	1.465
Self-Sample and take to a pharmacy for analysis	1.155	1.131	1.180	1.270	1.021	1.185	1.220	1.109	0.945	1.277
Self-Sample and take to place of education/work for analysis	0.821	0.919	0.733	0.804	0.806	0.852	0.851	0.778	0.761	0.861
GP Practice – sample taken by a healthcare professional	1.019	1.049	0.990	1.085	1.035	0.941	0.994	1.066	0.885	1.101
Sexual Health Clinic – sample taken by a healthcare professional	1.000	1.000	1.000	1.000	1.000	1.000	1.000	1.000	1.000	1.000
**2. Time to Result**										
30 Mins	1.806	1.828	1.786	1.763	1.888	1.772	1.730	1.896	1.808	1.797
2 Hours	1.402	1.365	1.442	1.326	1.548	1.344	1.311	1.516	1.357	1.423
7 Days	1.000	1.000	1.000	1.000	1.000	1.000	1.000	1.000	1.000	1.000
14 Days	0.862	0.844	0.881	0.878	0.882	0.828	0.862	0.862	0.882	0.856
**3. Accuracy**										
2 in 100 False Negative	3.242	2.951	3.570	3.307	3.161	3.282	3.275	3.305	3.000	3.482
5 in 100 False Negative	1.000	1.000	1.000	1.000	1.000	1.000	1.000	1.000	1.000	1.000
**4. Consultation Method**										
Online Consultation	1.212	1.187	1.239	1.187	1.200	1.253	1.156	1.285	1.174	1.228
Pharmacy Consultation	1.158	1.106	1.215	1.167	1.209	1.102	1.115	1.225	1.098	1.183
GP Consultation	1.183	1.172	1.194	1.279	1.142	1.133	1.162	1.189	1.112	1.215
Sexual Health Clinic Consultation	1.000	1.000	1.000	1.000	1.000	1.000	1.000	1.000	1.000	1.000
**5. Access to Healthcare Professional**										
Phone	0.949	0.937	0.962	1.001	0.902	0.946	0.942	0.926	0.947	0.953
Instant Messenger	1.027	0.994	1.062	1.108	0.977	1.001	1.025	0.991	1.045	1.025
Email	1.048	1.034	1.062	1.078	1.038	1.030	1.069	1.001	1.049	1.054
Face to Face	1.000	1.000	1.000	1.000	1.000	1.000	1.000	1.000	1.000	1.000
**6. How you get Antibiotics**										
Post to Home	1.011	0.996	1.028	0.977	1.010	1.048	0.957	1.055	1.011	1.020
Post to Collection Point	1.031	1.082	0.983	1.010	1.036	1.047	0.968	1.072	1.034	1.035
Collect from Pharmacy	1.075	1.064	1.087	1.039	1.106	1.080	1.070	1.056	1.086	1.078
Collect from Sexual Health Clinic	1.000	1.000	1.000	1.000	1.000	1.000	1.000	1.000	1.000	1.000

Table 4 – Trade-off between Accuracy and Time (Probability of Uptake)

For peer review only - http://bmjopen.bmj.com/site/about/guidelines.xhtml

	Full Dataset	Male	Female	16-18	19-21	22-24	Single	One Partner	Prev Test	No Prev Test
Pathways: (n in 100[1] / time[2])										
5 in 100[1]/ 30min[2]	38%	40%	35%	36%	39%	38%	36%	39%	40%	36%
2 in 100/ 14 days	58%	55%	62%	59%	58%	59%	59%	58%	58%	59%
Not Test	4%	5%	3%	5%	3%	3%	4%	3%	2%	5%
Total	100%	100%	100%	100%	100%	100%	100%	100%	100%	100%

[1] Test accuracy = n in 100 people will be told they don't have chlamydia when they do

[2] Time to receipt of result

For peer review only - http://bmjopen.bmj.com/site/about/guidelines.xhtml

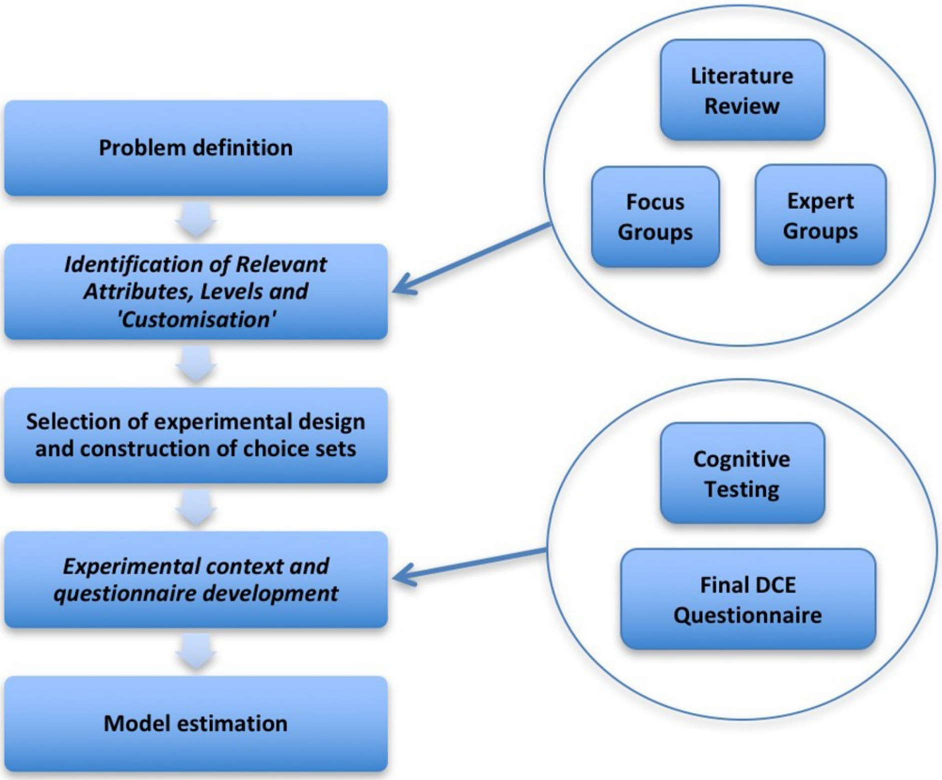

Figure 1 – Stages in Conducting a Discrete Choice Experiment

90x90mm (300 x 300 DPI)

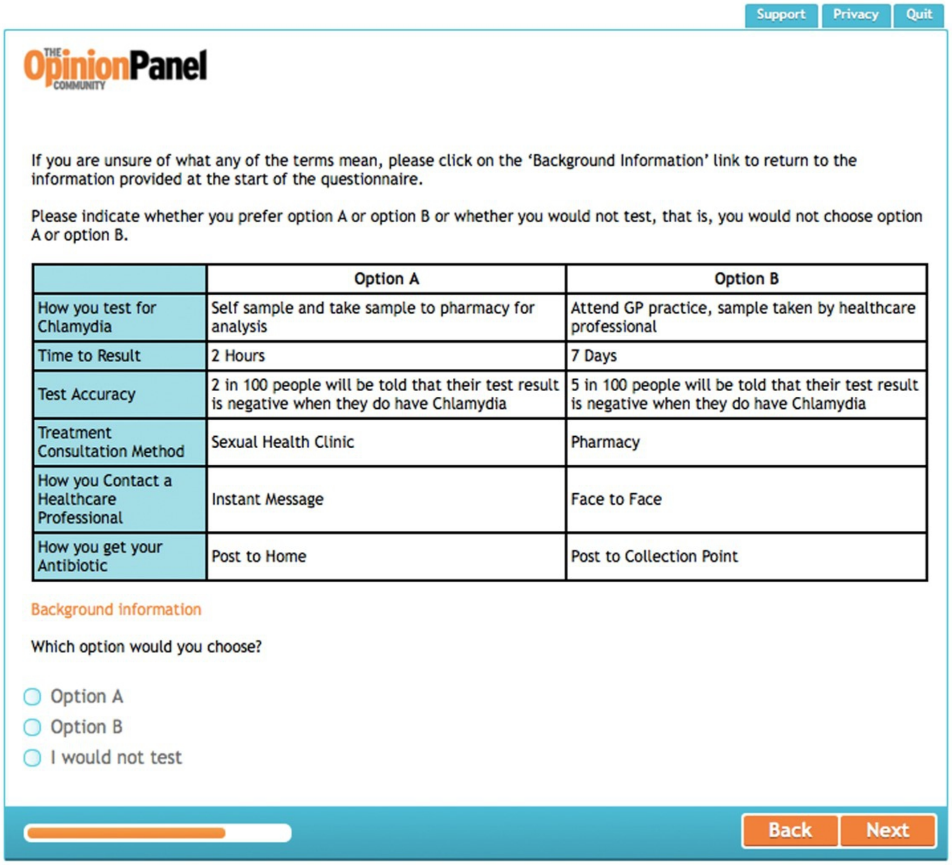

Support | Privacy | Quit

THE OpinionPanel COMMUNITY

If you are unsure of what any of the terms mean, please click on the 'Background Information' link to return to the information provided at the start of the questionnaire.

Please indicate whether you prefer option A or option B or whether you would not test, that is, you would not choose option A or option B.

	Option A	Option B
How you test for Chlamydia	Self sample and take sample to pharmacy for analysis	Attend GP practice, sample taken by healthcare professional
Time to Result	2 Hours	7 Days
Test Accuracy	2 in 100 people will be told that their test result is negative when they do have Chlamydia	5 in 100 people will be told that their test result is negative when they do have Chlamydia
Treatment Consultation Method	Sexual Health Clinic	Pharmacy
How you Contact a Healthcare Professional	Instant Message	Face to Face
How you get your Antibiotic	Post to Home	Post to Collection Point

Background information

Which option would you choose?

- Option A
- Option B
- I would not test

Back | Next

Figure 2 – Sample Choice Set from the Discrete Choice Experiment

99x90mm (300 x 300 DPI)

Supplementary File 1 – DCE Questionnaire Introduction and Background Information

Developing Chlamydia Testing & Treatment Services

This survey is designed to find out more about your views on what is important to you in accessing services to get tested for chlamydia, and treatment if your test result is positive (i.e. you have chlamydia). This forms part of a research project being undertaken at the University of Warwick, please click here to access further information about the study and how your information will be used [insert url to participant information leaflet]. By continuing to complete the survey you are consenting for your responses to be used for research in accordance with the information set out in the study information leaflet. Your responses will help us understand what is important to young people to shape how services are developed in the future.

Please read through the following background information before completing the survey. It contains information about chlamydia and the terms that are used in the survey.

What is Chlamydia?

Chlamydia is the most common sexually transmitted infection in England and the majority of infections are found in young people aged 15-24. Both men and women can get chlamydia, but most people with chlamydia have no symptoms and do not know they have an infection. The test for chlamydia is usually a urine sample for men and a vaginal swab for women. Once diagnosed, chlamydia can be treated with a single dose (2 or 4 tablets) of an antibiotic called Azithromycin.

In England there is a national chlamydia screening programme which recommends that people aged 16-24 are tested for chlamydia annually or when they change their sexual partner.

What happens if I don't get treatment?

If left undiagnosed chlamydia can lead to serious health problems.

In women this includes a condition called Pelvic Inflammatory Disease, which is an infection of the womb, fallopian tubes or ovaries. This can cause severe pain and in some cases (around 1 in 10) lead to infertility or ectopic pregnancy (where the pregnancy occurs outside of the womb in the fallopian tube). If you are pregnant and have chlamydia you can pass it on to your baby when you give birth and this can lead to the baby getting an eye infection or pneumonia.

For peer review only - http://bmjopen.bmj.com/site/about/guidelines.xhtml

In men chlamydia can lead to a condition called epididymitis, which causes soreness and swelling in the scrotum.

For further information

If you would like further information on chlamydia and how to prevent it, or any of the other conditions described please visit:
http://www.nhs.uk/conditions/chlamydia/Pages/Introduction.aspx

About the Survey…

In completing the survey you will be presented with a number of choices for getting tested and treatment for chlamydia. Please consider each set of choices and indicate whether you prefer option A or option B or whether you would not test, that is, you would not choose option A or option B.

Each choice involves two different scenarios for testing and getting treatment and there are differences between how you test, how long you have to wait for the result, how accurate the test is, how you have a consultation to get treatment for chlamydia, how you access a healthcare professional to get that treatment and how you get your antibiotic.

The following sections provide you with an explanation of the process of getting tested and treatment and the key terms used in the survey:

The diagram below shows the stages up until you find out our result:

For peer review only - http://bmjopen.bmj.com/site/about/guidelines.xhtml

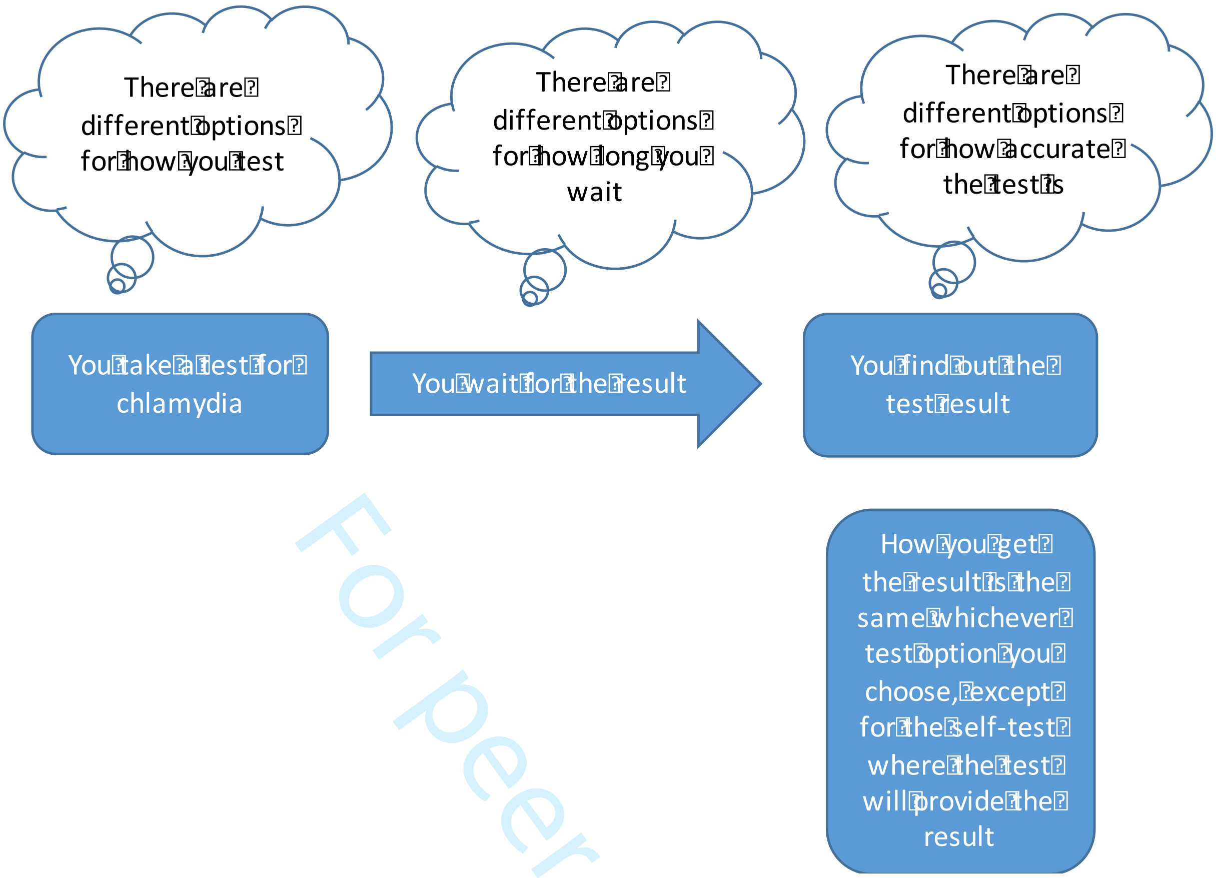

For peer review only

The following sections provide more information on how you test, how long you wait for the result and the accuracy of the test.

How you test for Chlamydia

This focuses on how you get your test, how the sample is taken and what happens to the sample once you've taken it. There are six options in the questionnaire, an explanation of these is provided below:

- **Self-Test -** Order a test kit online or collect one from a community location e.g. pharmacy or supermarket, provide the sample yourself and interpret the result yourself (like a pregnancy test). This is different to the self-sample options below because you interpret the result yourself rather than it being interpreted by a healthcare professional.
- **Self-Sample and post off for analysis -** Order a test kit online or collect one from a community location e.g. pharmacy or supermarket, provide the sample yourself and send the sample in the freepost envelope to the laboratory for analysis. This is different to the self-test option above because the test is interpreted by a healthcare professional.
- **Self-Sample and take to pharmacy for analysis -** Order a test kit online or collect one from a community location e.g. pharmacy or supermarket, provide the sample yourself and then take the sample to a pharmacy for analysis. This is different to the self-test option above because the test is interpreted by a

For peer review only - http://bmjopen.bmj.com/site/about/guidelines.xhtml

healthcare professional. Opening hours vary between pharmacies but many in towns and cities are open evenings and weekends as well as weekdays.

- **Self-Sample and take to your place of education/ workplace for analysis -** Order a test kit online or collect one from a community location e.g. pharmacy or supermarket, provide the sample yourself and take the sample to your place of education/ workplace for analysis by an outreach nurse. This is different to the self-test option above because the test is interpreted by a healthcare professional. Outreach services in education and workplaces are usually available one day a week.
- **Attend GP Practice, sample taken by a GP or Nurse -** Book an appointment at your GP practice with a GP or practice nurse. Your sample will be taken by the healthcare professional you see. GP practices are routinely open Monday to Friday 8am – 6.30pm.
- **Attend sexual health clinic, sample taken by a Doctor or Nurse -** Drop in or book an appointment with a doctor or nurse at a sexual health clinic. Your sample will be taken by the health care professional you see. Sexual Health Clinics are located in towns and cities, opening hours vary but clinics are generally open during the working day Monday to Friday, some evenings during the week and Saturdays.

Taking a sample yourself is straightforward and does not affect the accuracy of the test result.

Time to Result

How long it takes to get the test result. For the self-test option, this is how long it takes from you doing the test to you being able to read the result. For the other options this is how long it takes from you posting or dropping off your sample to when you are given your result. There are four options:

- 30 Mins
- 2 Hours
- 7 Days
- 14 Days

The time it takes to get the result does not affect the accuracy of the test.

Test Accuracy

This tells you how accurate your test result is. In this survey we want you to consider the likelihood of the test telling you that you don't have chlamydia when in fact you do. There are two options:

1. 2 in 100 people will be told that their test result is negative when they do have chlamydia
2. 5 in 100 people will be told their test result is negative when they do have chlamydia

For peer review only - http://bmjopen.bmj.com/site/about/guidelines.xhtml

Another way of describing this is shown below:

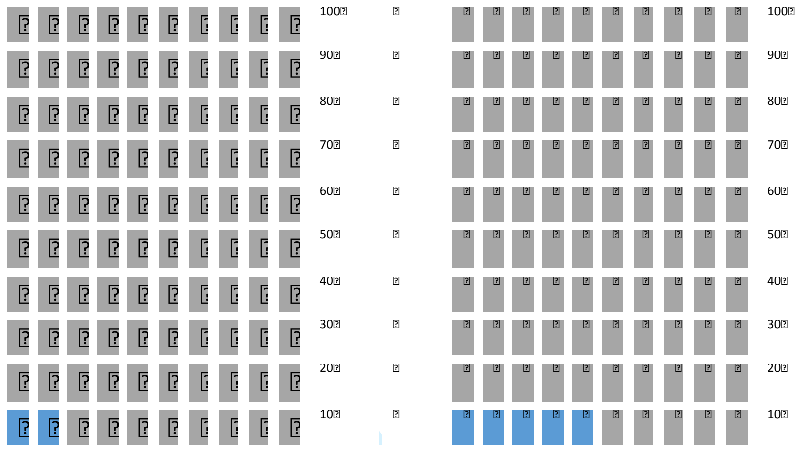

Each graph represents 100 people

= People who are told their test result is negative when they do have chlamydia

We would also like you to think about how you would choose to get treatment if your test result is positive. The diagram below shows the stages to get treatment:

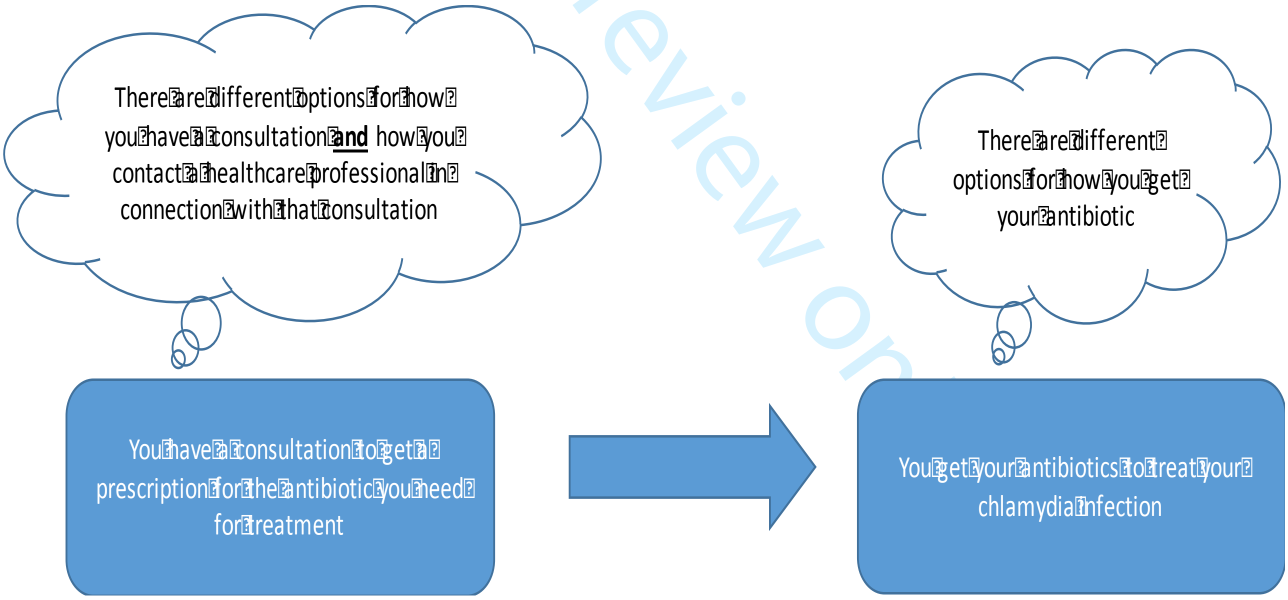

The following sections provide more information on how you get your treatment, how you contact a healthcare professional and how you get your antibiotics.

How you get your treatment

If your test result is positive there are a number of different options for you to get your treatment for chlamydia. There are four options in this survey:

For peer review only - http://bmjopen.bmj.com/site/about/guidelines.xhtml

- Complete an online consultation – this is available 24 hours a day, 7 days a week. You can access a healthcare professional if you need any help or advice between 9am and 5pm Monday to Friday.
- Have a consultation with a pharmacist - Opening hours vary between pharmacies but many in towns and cities are open evenings and weekends as well as weekdays.
- Have a consultation with a GP - GP practices are routinely open Monday to Friday 8am – 6.30pm.
- Have a consultation with a doctor or nurse at a Sexual Health Clinic - Sexual Health Clinics are located in towns and cities, opening hours vary but clinics are generally open during the working day Monday to Friday, some evenings during the week and Saturdays.

How you Contact a Healthcare Professional

New technology means that there are now more options for completing your consultation to get treatment, or in the case of the online consultation, accessing a healthcare professional for advice. There are four options within the questionnaire:

- Telephone – speak to a healthcare professional on the telephone.
- Instant Messaging – have a discussion with a healthcare professional via an instant messaging service online
- Email – have a discussion with a healthcare professional via email. During opening hours you will receive a response within 2 hours, and for emails sent out of hours you will receive a response the next day.
- Face-to-face - have a face-to-face discussion with a healthcare professional.

How you get your antibiotics

Once you've completed your consultation you need to get your antibiotic tablets. In this survey there are four options available to you:

- Deliver to home address – provide your home address for your antibiotic to be posted to you. Your antibiotic will arrive within 1-2 working days.
- Deliver to collection point – nominate an address other than your home address, for example a friend's address or a collection point for your antibiotic to be posted to. Your antibiotic will arrive within 1-2 working days.
- Collect from Pharmacy – attend a pharmacy of your choice with your prescription and the pharmacist gives you the antibiotic tablets
- Collect from Sexual Health Clinic – attend a sexual health clinic and the doctor or nurse gives you the antibiotic tablets

In all of the choices available to you there are a number of things that you should assume are the same:

- The service is provided to you free of charge although there may be a cost to you, for example in making phone calls, accessing the internet, or taking time off work to go to an appointment.

For peer review only - http://bmjopen.bmj.com/site/about/guidelines.xhtml

- The healthcare professional who is providing your treatment is trained to be able to provide the service.
- How you get your results is the same whichever option you choose, except for the self-test where the test will provide the result.
- Your personal data is managed securely in whichever option you choose

More choices for an option does not mean that it is more or less important, it just reflects that there are more choices within the scenarios you are being asked to consider.

For peer review only

Supplementary File 2 – DCE Results

Part 1 - Demographic Characteristics for Sub-Group Analysis

Demographic Characteristic	n	%	National %[1]
**Age**			
16	8	1%	10%
17	113	9%	10%
18	294	24%	11%
19	183	15%	11%
20	132	11%	11%
21	91	7%	11%
22	162	13%	11%
23	135	11%	12%
24	112	9%	12%
Total	1230	100%	99.00%
**Gender**			
Male	607	49%	51%
Female	623	51%	49%
Total	1230	100%	100%
**Ethnicity**			
White – English, Welsh, Scottish, Northern Irish, British	932	76%	80%
White – Irish	7	1%	1%
White – Gypsy or Irish Traveller	1	0%	0%
White – Any other white background	37	3%	5%
Mixed/ Multiple Ethnic Groups – White & Black Caribbean	10	1%	1%
Mixed/ Multiple Ethnic Groups – White & Black African	6	0%	0%
Mixed/ Multiple Ethnic Groups – White & Asian	13	1%	1%
Mixed/ Multiple Ethnic Groups – Any other mixed/ multiple ethnic background	8	1%	1%
Asian/ Asian British – Indian	50	4%	3%
Asian/ Asian British - Pakistani	38	3%	2%
Asian/ Asian British - Bangladeshi	18	1%	1%
Asian/ Asian British - Chinese	22	2%	1%
Asian/ Asian British – Any other Asian background	21	2%	2%
Black/ African/ Caribbean/ Black British – African	26	2%	2%
Black/ African/ Caribbean/ Black British – Caribbean	10	1%	1%

[1] - National Data for Age and Region taken from ONS Mid-Year Population Estimates 2015 (ONS, 2016b). Percentages derived from the total 16-24 population. National Data for Ethnicity taken from Census 2011 data, percentages derived from total England population (ONS, 2012). Percentages may not sum due to rounding

Demographic Characteristic	n	%	National %[1]
Black/ African/ Caribbean/ Black British – Any other black/ African/ Caribbean background	2	0%	1%
Other – Arab	5	0%	0%
Other – Any other ethnic group	9	1%	1%
I would prefer not to say	15	1%	0%
Total	1230	100%	103.00%
**Region**			
East Midlands	117	10%	9%
London	225	18%	15%
North East	74	6%	5%
North West	123	10%	13%
Eastern	55	4%	10%
South East	222	18%	16%
South West	143	12%	10%
West Midlands	144	12%	11%
Yorkshire and The Humber	113	9%	11%
I would prefer not to say	14	1%	0%
Total	1230	100%	100.00%
**Sexual Preference**			
Heterosexual (partner of opposite sex)	1030	84%	
Homosexual (partner of same sex) & Male	13	1%	
Homosexual (partner of same sex) & Female	39	3%	
Bisexual (partner of either sex)	98	8%	
I would prefer not to say	50	4%	
Total	1230	100%	
**Previous STI Test**			
Yes	393	32%	
No	790	64%	
I would prefer not to say	47	4%	
Total	1230	100%	
**Relationship Status**			
Single	615	50%	
In a non-sexual relationship with one person	36	3%	
In a non-sexual relationship with more than one person	2	0%	
In a sexual relationship with one person	512	42%	
In a sexual relationship with more than one person	36	3%	
I would prefer not to say	29	2%	
Total	1230	100%	

Part 2 - Validity Checks

Table 2.1 – Comparison of Full Dataset Coefficients and Odds Ratios for the Full Dataset for Dataset including Choice Set 1 and Dataset including Choice Set 25. Data highlighted in blue indicate results which are not statistically significant.

	**Dataset Including Choice Set 1, *n=1,230***						**Dataset Including Choice Set 25, *n=1,230***					
	**Coefficient**	**Lower 95% CI**	**Upper 95% CI**	**Odds Ratio**	**Lower 95% CI**	**Upper 95% CI**	**Coefficient**	**Lower 95% CI**	**Upper 95% CI**	**Odds Ratio**	**Lower 95% CI**	**Upper 95% CI**
**How you Test**												
Self-Test	0.498	0.432	0.564	1.646	1.540	1.758	0.481	0.415	0.547	1.618	1.514	1.729
Post	0.308	0.239	0.376	1.360	1.270	1.456	0.306	0.239	0.373	1.358	1.271	1.452
Pharmacy	0.116	0.056	0.175	1.123	1.057	1.192	0.144	0.085	0.203	1.155	1.088	1.226
Education/ Work	-0.206	-0.266	-0.146	0.814	0.766	0.864	-0.197	-0.257	-0.137	0.821	0.773	0.872
GP Practice	0.011	-0.050	0.073	1.011	0.951	1.075	0.019	-0.042	0.081	1.019	0.959	1.084
Sexual Health Clinic	0.000	-	-	1.000	-	-	0.000	-	-	1.000	-	-
**Time to Result**												
30 Mins	0.579	0.524	0.633	1.784	1.688	1.884	0.591	0.537	0.645	1.806	1.711	1.906
2 Hours	0.340	0.283	0.398	1.406	1.328	1.488	0.338	0.281	0.395	1.402	1.324	1.485
7 Days	0.000	-	-	1.000	-	-	0.000	-	-	1.000	-	-
14 Days	-0.149	-0.200	-0.099	0.861	0.819	0.906	-0.148	-0.198	-0.098	0.862	0.820	0.907
**Accuracy**												
2 in 100 False Negative	1.192	1.157	1.226	3.292	3.179	3.409	1.176	1.141	1.212	3.242	3.130	3.359
5 in 100 False Negative	0.000	-	-	1.000	-	-	0.000	-	-	1.000	-	-

For peer review only

	Dataset Including Choice Set 1, *n=1,230*						Dataset Including Choice Set 25, *n=1,230*					
	Coefficient	Lower 95% CI	Upper 95% CI	Odds Ratio	Lower 95% CI	Upper 95% CI	Coefficient	Lower 95% CI	Upper 95% CI	Odds Ratio	Lower 95% CI	Upper 95% CI
**Consultation Method**												
Online Consultation	0.185	0.133	0.236	1.203	1.142	1.266	0.192	0.140	0.245	1.212	1.150	1.277
Pharmacy Consultation	0.150	0.099	0.202	1.162	1.104	1.224	0.147	0.095	0.199	1.158	1.100	1.220
GP Consultation	0.146	0.095	0.197	1.157	1.100	1.218	0.168	0.116	0.220	1.183	1.123	1.246
Sexual Health Clinic Consultation	0.000	-	-	1.000	-	-	0.000	-	-	1.000	-	-
**Access to Healthcare Professional**												
Phone	-0.058	-0.108	-0.008	0.943	0.897	0.992	-0.052	-0.102	-0.002	0.949	0.903	0.998
Instant Messenger	0.020	-0.029	0.070	1.020	0.971	1.072	0.027	-0.023	0.076	1.027	0.977	1.079
Email	0.027	-0.023	0.076	1.027	0.978	1.079	0.047	-0.002	0.096	1.048	0.998	1.101
Face to Face	0.000	-	-	1.000	-	-	0.000	-	-	1.000	-	-
**How you get Antibiotics**												
Post to Home	0.038	-0.013	0.089	1.039	0.987	1.093	0.011	-0.041	0.063	1.011	0.960	1.065
Post to Collection Point	0.052	0.002	0.101	1.053	1.002	1.106	0.030	-0.020	0.081	1.031	0.980	1.085
Collect from Pharmacy	0.091	0.038	0.143	1.095	1.039	1.154	0.072	0.018	0.126	1.075	1.018	1.134
Collect from Sexual Health Clinic	0.000	-	-	1.000	-	-	0.000	-	-	1.000	-	-
**Choice**												
Option A	0.000	-	-	1.000	-	-	0.000	-	-	1.000	-	-
Option B	-0.341	-0.381	-0.302	0.711	0.683	0.740	-0.360	-0.400	-0.320	0.698	0.670	0.726
I would not test	-1.697	-1.797	-1.596	0.183	0.166	0.203	-1.682	-1.781	-1.583	0.186	0.168	0.205

For peer review only

Table 2.2 – Coefficients and Odds Ratios for the Full Dataset and the Dataset Excluding Different Responses to Choice Sets 1 and 25. Data highlighted in blue indicate results which are not statistically significant.

	**Full Dataset *n=1,230***						**Excl Incorrect Answer to Validity Check *n=912***					
	**Coefficient**	**Lower 95% CI**	**Upper 95% CI**	**Odds Ratio**	**Lower 95% CI**	**Upper 95% CI**	**Coefficient**	**Lower 95% CI**	**Upper 95% CI**	**Odds Ratio**	**Lower 95% CI**	**Upper 95% CI**
**How you Test**												
Self-Test	0.481	0.415	0.547	1.618	1.514	1.729	0.507	0.429	0.584	1.660	1.535	1.794
Post	0.306	0.239	0.373	1.358	1.271	1.452	0.333	0.254	0.411	1.395	1.290	1.508
Pharmacy	0.144	0.085	0.203	1.155	1.088	1.226	0.124	0.054	0.194	1.132	1.056	1.214
Education/ Work	-0.197	-0.257	-0.137	0.821	0.773	0.872	-0.253	-0.323	-0.183	0.777	0.724	0.833
GP Practice	0.019	-0.042	0.081	1.019	0.959	1.084	0.019	-0.053	0.091	1.019	0.948	1.095
Sexual Health Clinic	0.000	-	-	1.000	-	-	0.000	-	-	1.000	-	-
**Time to Result**												
30 Mins	0.591	0.537	0.645	1.806	1.711	1.906	0.573	0.510	0.637	1.774	1.665	1.890
2 Hours	0.338	0.281	0.395	1.402	1.324	1.485	0.335	0.268	0.402	1.397	1.307	1.494
7 Days	0.000	-	-	1.000	-	-	0.000	-	-	1.000	-	-
14 Days	-0.148	-0.198	-0.098	0.862	0.820	0.907	-0.148	-0.177	-0.236	-0.117	0.790	0.889
**Accuracy**												
2 in 100 False Negative	1.176	1.141	1.212	3.242	3.130	3.359	1.323	1.282	1.364	3.755	3.602	3.913
5 in 100 False Negative	0.000	-	-	1.000	-	-	0.000	-	-	1.000	-	-
**Consultation Method**												
Online Consultation	0.192	0.140	0.245	1.212	1.150	1.277	0.197	0.136	0.258	1.218	1.145	1.294
Pharmacy Consultation	0.147	0.095	0.199	1.158	1.100	1.220	0.173	0.112	0.234	1.189	1.119	1.264
GP Consultation	0.168	0.116	0.220	1.183	1.123	1.246	0.179	0.119	0.240	1.196	1.126	1.271
Sexual Health Clinic Consultation	0.000	-	-	1.000	-	-	0.000	-	-	1.000	-	-

	**Full Dataset *n=1,230***						**Excl Incorrect Answer to Validity Check *n=912***					
	**Coefficient**	**Lower 95% CI**	**Upper 95% CI**	**Odds Ratio**	**Lower 95% CI**	**Upper 95% CI**	**Coefficient**	**Lower 95% CI**	**Upper 95% CI**	**Odds Ratio**	**Lower 95% CI**	**Upper 95% CI**
**Access to Healthcare Professional**												
Phone	-0.052	-0.102	-0.002	0.949	0.903	0.998	-0.084	-0.143	-0.026	0.919	0.867	0.975
Instant Messenger	0.027	-0.023	0.076	1.027	0.977	1.079	-0.026	-0.084	0.031	0.974	0.919	1.032
Email	0.047	-0.002	0.096	1.048	0.998	1.101	-0.005	-0.063	0.052	0.995	0.939	1.054
Face to Face	0.000	-	-	1.000	-	-	0.000	-	-	1.000	-	-
**How you get Antibiotics**												
Post to Home	0.011	-0.041	0.063	1.011	0.960	1.065	0.014	-0.047	0.075	1.014	0.954	1.078
Post to Collection Point	0.030	-0.020	0.081	1.031	0.980	1.085	0.023	-0.036	0.083	1.024	0.964	1.086
Collect from Pharmacy	0.072	0.018	0.126	1.075	1.018	1.134	0.065	0.001	0.128	1.067	1.001	1.137
Collect from Sexual Health Clinic	0.000	-	-	1.000	-	-	0.000	-	-	1.000	-	-
**Choice**												
Option A	0.000	-	-	1.000	-	-	0.000	-	-	1.000	-	-
Option B	-0.360	-0.400	-0.320	0.698	0.670	0.726	-0.327	-0.374	-0.279	0.721	0.688	0.756
I would not test	-1.682	-1.781	-1.583	0.186	0.168	0.205	-1.674	-1.790	-1.557	0.188	0.167	0.211

For peer review only

Table 2.3 – Coefficient and Odds Ratios for the Full Dataset and the Dataset Containing Five Minutes or Longer to Complete. Data highlighted in blue indicate results which are not statistically significant.

	Full Dataset *n=1,230*						Data Excluding Responses taking less than 5 min to complete *n= 997*					
	Coefficient	Lower 95% CI	Upper 95% CI	Odds Ratio	Lower 95% CI	Upper 95% CI	Coefficient	Lower 95% CI	Upper 95% CI	Odds Ratio	Lower 95% CI	Upper 95% CI
**How you Test**												
Self-Test	0.481	0.415	0.547	1.618	1.514	1.729	0.542	0.467	0.616	1.719	1.595	1.852
Post	0.306	0.239	0.373	1.358	1.271	1.452	0.344	0.269	0.419	1.411	1.309	1.521
Pharmacy	0.144	0.085	0.203	1.155	1.088	1.226	0.168	0.101	0.234	1.183	1.106	1.264
Education/ Work	-0.197	-0.257	-0.137	0.821	0.773	0.872	-0.245	-0.312	-0.177	0.783	0.732	0.837
GP Practice	0.019	-0.042	0.081	1.019	0.959	1.084	-0.008	-0.077	0.061	0.992	0.925	1.062
Sexual Health Clinic	0.000	-	-	1.000	-	-	0.000	-	-	1.000	-	-
**Time to Result**												
30 Mins	0.591	0.537	0.645	1.806	1.711	1.906	0.642	0.581	0.703	1.900	1.788	2.019
2 Hours	0.338	0.281	0.395	1.402	1.324	1.485	0.372	0.308	0.436	1.451	1.361	1.547
7 Days	0.000	-	-	1.000	-	-	0.000	-	-	1.000	-	-
14 Days	-0.148	-0.198	-0.098	0.862	0.820	0.907	-0.147	-0.204	-0.091	0.863	0.816	0.913
**Accuracy**												
2 in 100 False Negative	1.176	1.141	1.212	3.242	3.130	3.359	1.308	1.269	1.348	3.700	3.556	3.850
5 in 100 False Negative	0.000	-	-	1.000	-	-	0.000	-	-	1.000	-	-
**Consultation Method**												
Online Consultation	0.192	0.140	0.245	1.212	1.150	1.277	0.217	0.159	0.276	1.243	1.172	1.318
Pharmacy Consultation	0.147	0.095	0.199	1.158	1.100	1.220	0.160	0.102	0.219	1.174	1.108	1.245
GP Consultation	0.168	0.116	0.220	1.183	1.123	1.246	0.171	0.113	0.229	1.187	1.120	1.258
Sexual Health Clinic Consultation	0.000	-	-	1.000	-	-	0.000	-	-	1.000	-	-
**Access to Healthcare Professional**												
Phone	-0.052	-0.102	-0.002	0.949	0.903	0.998	-0.042	-0.098	0.014	0.959	0.906	1.014
Instant Messenger	0.027	-0.023	0.076	1.027	0.977	1.079	0.043	-0.013	0.098	1.044	0.987	1.103
Email	0.047	-0.002	0.096	1.048	0.998	1.101	0.042	-0.013	0.097	1.043	0.987	1.102
Face to Face	0.000	-	-	1.000	-	-	0.000	-	-	1.000	-	-

For peer review only

	Full Dataset *n=1,230*						Data Excluding Responses taking less than 5 min to complete *n= 997*					
	Coefficient	Lower 95% CI	Upper 95% CI	Odds Ratio	Lower 95% CI	Upper 95% CI	Coefficient	Lower 95% CI	Upper 95% CI	Odds Ratio	Lower 95% CI	Upper 95% CI
**How you get Antibiotics**												
Post to Home	0.011	-0.041	0.063	1.011	0.960	1.065	0.001	-0.057	0.060	1.001	0.944	1.062
Post to Collection Point	0.030	-0.020	0.081	1.031	0.980	1.085	0.013	-0.044	0.070	1.013	0.957	1.072
Collect from Pharmacy	0.072	0.018	0.126	1.075	1.018	1.134	0.085	0.024	0.145	1.089	1.025	1.157
Collect from Sexual Health Clinic	0.000	-	-	1.000	-	-	0.000	-	-	1.000	-	-
**Choice**												
Option A	0.000	-	-	1.000	-	-	0.000	-	-	1.000	-	-
Option B	-0.360	-0.400	-0.320	0.698	0.670	0.726	-0.368	-0.413	-0.323	0.692	0.662	0.724
I would not test	-1.682	-1.781	-1.583	0.186	0.168	0.205	-1.776	-1.889	-1.662	0.169	0.151	0.190

For peer review only

Table 2.4 – Comparison of Full Dataset and Dataset Excluding Opt Out Responses. Data highlighted in blue indicate results which are not statistically significant.

	**Full Dataset *n=29,520 Choice Responses***						**Removal of 'I would not test' Data *n=27,727 Choice Responses***					
	**Coefficient**	**Lower 95% CI**	**Upper 95% CI**	**Odds Ratio**	**Lower 95% CI**	**Upper 95% CI**	**Coefficient**	**Lower 95% CI**	**Upper 95% CI**	**Odds Ratio**	**Lower 95% CI**	**Upper 95% CI**
**How you Test**												
Self-Test	0.481	0.415	0.547	1.618	1.514	1.729	0.501	0.433	0.568	1.650	1.542	1.765
Post	0.306	0.239	0.373	1.358	1.271	1.452	0.320	0.252	0.387	1.377	1.287	1.473
Pharmacy	0.144	0.085	0.203	1.155	1.088	1.226	0.152	0.092	0.212	1.164	1.096	1.236
Education/ Work	-0.197	-0.257	-0.137	0.821	0.773	0.872	-0.202	-0.262	-0.141	0.817	0.769	0.868
GP Practice	0.019	-0.042	0.081	1.019	0.959	1.084	0.027	-0.035	0.089	1.027	0.965	1.093
Sexual Health Clinic	0.000	-	-	1.000	-	-	0.000	-	-	1.000	-	-
**Time to Result**												
30 Mins	0.591	0.537	0.645	1.806	1.711	1.906	0.612	0.558	0.667	1.845	1.746	1.949
2 Hours	0.338	0.281	0.395	1.402	1.324	1.485	0.350	0.292	0.408	1.419	1.339	1.503
7 Days	0.000	-	-	1.000	-	-	0.000	-	-	1.000	-	-
14 Days	-0.148	-0.198	-0.098	0.862	0.820	0.907	-0.145	-0.196	-0.094	0.865	0.822	0.910
**Accuracy**												
2 in 100 False Negative	1.176	1.141	1.212	3.242	3.130	3.359	1.205	1.170	1.241	3.338	3.221	3.460
5 in 100 False Negative	0.000	-	-	1.000	-	-	0.000	-	-	1.000	-	-
**Consultation Method**												
Online Consultation	0.192	0.140	0.245	1.212	1.150	1.277	0.200	0.147	0.253	1.222	1.159	1.288
Pharmacy Consultation	0.147	0.095	0.199	1.158	1.100	1.220	0.159	0.106	0.211	1.172	1.112	1.235
GP Consultation	0.168	0.116	0.220	1.183	1.123	1.246	0.174	0.122	0.227	1.190	1.130	1.254
Sexual Health Clinic Consultation	0.000	-	-	1.000	-	-	0.000	-	-	1.000	-	-
**Access to Healthcare Professional**												
Phone	-0.052	-0.102	-0.002	0.949	0.903	0.998	-0.046	-0.097	0.004	0.955	0.908	1.004
Instant Messenger	0.027	-0.023	0.076	1.027	0.977	1.079	0.029	-0.021	0.079	1.029	0.979	1.082
Email	0.047	-0.002	0.096	1.048	0.998	1.101	0.050	0.000	0.100	1.051	1.000	1.105
Face to Face	0.000	-	-	1.000	-	-	0.000	-	-	1.000	-	-

	Full Dataset *n=29,520 Choice Responses*						Removal of 'I would not test' Data *n=27,727 Choice Responses*					
	**Coefficient**	**Lower 95% CI**	**Upper 95% CI**	**Odds Ratio**	**Lower 95% CI**	**Upper 95% CI**	**Coefficient**	**Lower 95% CI**	**Upper 95% CI**	**Odds Ratio**	**Lower 95% CI**	**Upper 95% CI**
**How you get Antibiotics**												
Post to Home	0.011	-0.041	0.063	1.011	0.960	1.065	0.012	-0.041	0.065	1.012	0.960	1.067
Post to Collection Point	0.030	-0.020	0.081	1.031	0.980	1.085	0.035	-0.016	0.086	1.036	0.984	1.090
Collect from Pharmacy	0.072	0.018	0.126	1.075	1.018	1.134	0.076	0.021	0.131	1.079	1.022	1.139
Collect from Sexual Health Clinic	0.000	-	-	1.000	-	-	0.000	-	-	1.000	-	-
**Choice**												
Option A	0.000	-	-	1.000	-	-	0.000	-	-	1.000	-	-
Option B	-0.360	-0.400	-0.320	0.698	0.670	0.726	-0.368	-0.409	-0.327	0.692	0.664	0.721
I would not test	-1.682	-1.781	-1.583	0.186	0.168	0.205	-	-	-	1.000		

For peer review only

Part 3 – DCE Results

(Cells highlighted in blue indicate result which is not statistically significant)

Table 3.1- Full Dataset Coefficients, Odds Ratios and Respective 95% Confidence Interval

	**Full Dataset *n=1,230***					
	**Coefficient**	**Lower 95% CI**	**Upper 95% CI**	**Odds Ratio**	**Lower 95% CI**	**Upper 95% CI**
**How you Test**						
Self-Test	0.481	0.415	0.547	1.618	1.514	1.729
Post	0.306	0.239	0.373	1.358	1.271	1.452
Pharmacy	0.144	0.085	0.203	1.155	1.088	1.226
Education/ Work	-0.197	-0.257	-0.137	0.821	0.773	0.872
GP Practice	0.019	-0.042	0.081	1.019	0.959	1.084
Sexual Health Clinic	0.000	-	-	1.000	-	-
**Time to Result**						
30 Mins	0.591	0.537	0.645	1.806	1.711	1.906
2 Hours	0.338	0.281	0.395	1.402	1.324	1.485
7 Days	0.000	-	-	1.000	-	-
14 Days	-0.148	-0.198	-0.098	0.862	0.820	0.907
**Accuracy**						
2 in 100 False Negative	1.176	1.141	1.212	3.242	3.130	3.359
5 in 100 False Negative	0.000	-	-	1.000	-	-
**Consultation Method**						
Online Consultation	0.192	0.140	0.245	1.212	1.150	1.277
Pharmacy Consultation	0.147	0.095	0.199	1.158	1.100	1.220
GP Consultation	0.168	0.116	0.220	1.183	1.123	1.246
Sexual Health Clinic Consultation	0.000	-	-	1.000	-	-
**Access to Healthcare Professional**						
Phone	-0.052	-0.102	-0.002	0.949	0.903	0.998
Instant Messenger	0.027	-0.023	0.076	1.027	0.977	1.079
Email	0.047	-0.002	0.096	1.048	0.998	1.101
Face to Face	0.000	-	-	1.000	-	-
**How you get Antibiotics**						
Post to Home	0.011	-0.041	0.063	1.011	0.960	1.065
Post to Collection Point	0.030	-0.020	0.081	1.031	0.980	1.085
Collect from Pharmacy	0.072	0.018	0.126	1.075	1.018	1.134
Collect from Sexual Health Clinic	0.000	-	-	1.000	-	-

For peer review only

Table 3.2 - Gender Subgroup Analysis Coefficients and Odds Ratios with Associated 95% Confidence Intervals

	Males *n=607*						Females *n=623*					
	**Coefficient**	**Lower 95% CI**	**Upper 95% CI**	**Odds Ratio**	**Lower 95% CI**	**Upper 95% CI**	**Coefficient**	**Lower 95% CI**	**Upper 95% CI**	**Odds Ratio**	**Lower 95% CI**	**Upper 95% CI**
**How you Test**												
Self-Test	0.438	0.344	0.531	1.549	1.410	1.701	0.526	0.432	0.620	1.693	1.541	1.860
Post	0.269	0.174	0.363	1.308	1.190	1.438	0.344	0.250	0.439	1.411	1.284	1.551
Pharmacy	0.123	0.039	0.208	1.131	1.040	1.231	0.165	0.081	0.249	1.180	1.085	1.283
Education/ Work	-0.084	-0.169	0.001	0.919	0.845	1.001	-0.310	-0.395	-0.225	0.733	0.674	0.798
GP Practice	0.048	-0.039	0.135	1.049	0.962	1.145	-0.010	-0.097	0.077	0.990	0.908	1.080
Sexual Health Clinic	0.000	-	-	1.000	-	-	0.000	-	-	1.000	-	-
**Time to Result**												
30 Mins	0.603	0.527	0.680	1.828	1.694	1.973	0.580	0.503	0.656	1.786	1.654	1.928
2 Hours	0.311	0.230	0.392	1.365	1.259	1.479	0.366	0.285	0.447	1.442	1.329	1.563
7 Days	0.000	-	-	1.000	-	-	0.000	-	-	1.000	-	-
14 Days	-0.170	-0.241	-0.098	0.844	0.786	0.906	-0.127	-0.198	-0.056	0.881	0.820	0.945
**Accuracy**												
2 in 100 False Negative	1.082	1.032	1.132	2.951	2.807	3.101	1.273	1.223	1.322	3.570	3.396	3.753
5 in 100 False Negative	0.000	-	-	1.000	-	-	0.000	-	-	1.000	-	-
**Consultation Method**												
Online Consultation	0.171	0.097	0.245	1.187	1.102	1.278	0.214	0.140	0.288	1.239	1.151	1.334
Pharmacy Consultation	0.100	0.027	0.173	1.106	1.028	1.189	0.194	0.121	0.268	1.215	1.129	1.307
GP Consultation	0.159	0.085	0.232	1.172	1.089	1.262	0.178	0.104	0.251	1.194	1.110	1.285
Sexual Health Clinic Consultation	0.000	-	-	1.000	-	-	0.000	-	-	1.000	-	-
**Access to Healthcare Professional**												
Phone	-0.065	-0.136	0.005	0.937	0.873	1.005	-0.039	-0.110	0.032	0.962	0.896	1.032
Instant Messenger	-0.006	-0.076	0.063	0.994	0.927	1.065	0.060	-0.010	0.130	1.062	0.990	1.139
Email	0.033	-0.036	0.103	1.034	0.965	1.108	0.060	-0.009	0.130	1.062	0.991	1.139
Face to Face	0.000	-	-	1.000	-	-	0.000	-	-	1.000	-	-
**How you get Antibiotics**												
Post to Home	-0.004	-0.078	0.070	0.996	0.925	1.072	0.027	-0.046	0.101	1.028	0.955	1.106
Post to Collection Point	0.079	0.007	0.151	1.082	1.007	1.163	-0.017	-0.089	0.055	0.983	0.915	1.056
Collect from Pharmacy	0.062	-0.014	0.138	1.064	0.986	1.149	0.083	0.007	0.160	1.087	1.007	1.173
Collect from Sexual Health Clinic	0.000	-	-	1.000	-	-	0.000	-	-	1.000	-	-

For peer review only

Table 3.3 - Age Range Subgroup Analysis Coefficients and Odds Ratios, including Respective 95% Confidence Intervals

	**16-18 *n=415***						**19-21 *n=406***						**22-24 *n=409***					
	**Coeffi-cient**	**Lower 95% CI**	**Upper 95% CI**	**Odds Ratio**	**Lower 95% CI**	**Upper 95% CI**	**Coeffi-cient**	**Lower 95% CI**	**Upper 95% CI**	**Odds Ratio**	**Lower 95% CI**	**Upper 95% CI**	**Coeffi-cient**	**Lower 95% CI**	**Upper 95% CI**	**Odds Ratio**	**Lower 95% CI**	**Upper 95% CI**
**How you Test**																		
Self-Test	0.509	0.395	0.623	1.664	1.485	1.865	0.297	0.182	0.412	1.346	1.200	1.510	0.639	0.523	0.755	1.895	1.687	2.128
Post	0.390	0.275	0.505	1.477	1.316	1.657	0.129	0.013	0.245	1.138	1.013	1.277	0.397	0.281	0.513	1.487	1.324	1.670
Pharmacy	0.239	0.137	0.342	1.270	1.147	1.408	0.021	-0.082	0.124	1.021	0.921	1.132	0.170	0.067	0.273	1.185	1.069	1.314
Education/ Work	-0.218	-0.322	-0.114	0.804	0.725	0.892	-0.215	-0.319	-0.112	0.806	0.727	0.894	-0.160	-0.265	-0.056	0.852	0.768	0.945
GP Practice	0.081	-0.025	0.187	1.085	0.975	1.206	0.035	-0.072	0.141	1.035	0.931	1.152	-0.061	-0.168	0.046	0.941	0.846	1.047
Sexual Health Clinic	0.000	-	-	1.000	-	-	0.000	-	-	1.000	-	-	0.000	-	-	1.000	-	-
**Time to Result**																		
30 Mins	0.567	0.474	0.661	1.763	1.606	1.936	0.636	0.542	0.730	1.888	1.719	2.074	0.572	0.478	0.666	1.772	1.613	1.947
2 Hours	0.282	0.183	0.381	1.326	1.201	1.464	0.437	0.338	0.536	1.548	1.402	1.709	0.295	0.196	0.395	1.344	1.216	1.484
7 Days	0.000	-	-	1.000	-	-	0.000	-	-	1.000	-	-	0.000	-	-	1.000	-	-
14 Days	-0.130	-0.217	-0.044	0.878	0.805	0.957	-0.125	-0.213	-0.038	0.882	0.808	0.963	-0.189	-0.277	-0.102	0.828	0.758	0.903
**Accuracy**																		
2 in 100 False Negative	1.196	1.135	1.257	3.307	3.112	3.514	1.151	1.090	1.212	3.161	2.974	3.361	1.188	1.127	1.250	3.282	3.086	3.489
5 in 100 False Negative	0.000	-	-	1.000	-	-	0.000	-	-	1.000	-	-	0.000	-	-	1.000	-	-
**Consultation Method**																		
Online Consultation	0.171	0.081	0.262	1.187	1.085	1.299	0.182	0.092	0.273	1.200	1.096	1.314	0.226	0.135	0.317	1.253	1.144	1.373
Pharmacy Consultation	0.154	0.065	0.244	1.167	1.067	1.276	0.190	0.100	0.279	1.209	1.105	1.322	0.098	0.007	0.188	1.102	1.007	1.206
GP Consultation	0.246	0.157	0.335	1.279	1.170	1.398	0.133	0.043	0.223	1.142	1.044	1.250	0.125	0.035	0.215	1.133	1.036	1.240
Sexual Health Clinic Consultation	0.000	-	-	1.000	-	-	0.000	-	-	1.000	-	-	0.000	-	-	1.000	-	-

For peer review only

	16-18 *n=415*						19-21 *n=406*						22-24 *n=409*					
	**Coeffi-cient**	**Lower 95% CI**	**Upper 95% CI**	**Odds Ratio**	**Lower 95% CI**	**Upper 95% CI**	**Coeffi-cient**	**Lower 95% CI**	**Upper 95% CI**	**Odds Ratio**	**Lower 95% CI**	**Upper 95% CI**	**Coeffi-cient**	**Lower 95% CI**	**Upper 95% CI**	**Odds Ratio**	**Lower 95% CI**	**Upper 95% CI**
**Access to Healthcare Professional**																		
Phone	0.001	-0.085	0.087	1.001	0.918	1.091	-0.104	-0.190	-0.017	0.902	0.827	0.983	-0.055	-0.142	0.031	0.946	0.868	1.032
Instant Messenger	0.102	0.017	0.188	1.108	1.017	1.207	-0.024	-0.109	0.062	0.977	0.897	1.064	0.001	-0.085	0.087	1.001	0.918	1.091
Email	0.075	-0.009	0.160	1.078	0.991	1.174	0.037	-0.048	0.122	1.038	0.954	1.130	0.029	-0.056	0.115	1.030	0.945	1.122
Face to Face	0.000	-	-	1.000	-	-	0.000	-	-	1.000	-	-	0.000	-	-	1.000	-	-
**How you get Antibiotics**																		
Post to Home	-0.024	-0.114	0.066	0.977	0.893	1.068	0.010	-0.081	0.100	1.010	0.922	1.106	0.047	-0.043	0.137	1.048	0.957	1.147
Post to Collection Point	0.010	-0.078	0.097	1.010	0.925	1.102	0.035	-0.053	0.123	1.036	0.948	1.131	0.046	-0.042	0.135	1.047	0.959	1.144
Collect from Pharmacy	0.039	-0.054	0.132	1.039	0.947	1.141	0.101	0.008	0.195	1.106	1.008	1.215	0.077	-0.017	0.171	1.080	0.983	1.186
Collect from Sexual Health Clinic	0.000	-	-	1.000	-	-	0.000	-	-	1.000	-	-	0.000	-	-	1.000	-	-

For peer review only

Table 3.4 - Relationship Status Subgroup Analysis Coefficients and Odds Ratios, including Respective 95% Confidence Intervals

	Single *n=615*						Sexual Relationship with One Partner *n=512*					
	**Coefficient**	**Lower 95% CI**	**Upper 95% CI**	**Odds Ratio**	**Lower 95% CI**	**Upper 95% CI**	**Coefficient**	**Lower 95% CI**	**Upper 95% CI**	**Odds Ratio**	**Lower 95% CI**	**Upper 95% CI**
**How you Test**												
Self-Test	0.490	0.396	0.584	1.632	1.486	1.792	0.538	0.434	0.641	1.712	1.543	1.899
Post	0.322	0.227	0.416	1.380	1.255	1.516	0.322	0.218	0.425	1.380	1.244	1.530
Pharmacy	0.199	0.115	0.283	1.220	1.122	1.327	0.104	0.011	0.196	1.109	1.011	1.217
Education/ Work	-0.162	-0.247	-0.077	0.851	0.781	0.926	-0.251	-0.344	-0.157	0.778	0.709	0.854
GP Practice	-0.006	-0.093	0.081	0.994	0.911	1.085	0.063	-0.032	0.159	1.066	0.969	1.172
Sexual Health Clinic	0.000	-	-	1.000	-	-	0.000	-	-	1.000	-	-
**Time to Result**												
30 Mins	0.548	0.471	0.625	1.730	1.602	1.867	0.640	0.556	0.724	1.896	1.743	2.062
2 Hours	0.271	0.190	0.352	1.311	1.209	1.422	0.416	0.327	0.505	1.516	1.387	1.657
7 Days	0.000	-	-	1.000	-	-	0.000	-	-	1.000	-	-
14 Days	-0.148	-0.220	-0.077	0.862	0.803	0.926	-0.149	-0.227	-0.071	0.862	0.797	0.932
**Accuracy**												
2 in 100 False Negative	1.186	1.137	1.236	3.275	3.116	3.443	1.196	1.141	1.250	3.305	3.129	3.492
5 in 100 False Negative	0.000	-	-	1.000	-	-	0.000	-	-	1.000	-	-
**Consultation Method**												
Online Consultation	0.145	0.071	0.219	1.156	1.074	1.245	0.251	0.169	0.332	1.285	1.185	1.394
Pharmacy Consultation	0.108	0.035	0.182	1.115	1.036	1.199	0.203	0.123	0.284	1.225	1.130	1.328
GP Consultation	0.150	0.077	0.223	1.162	1.080	1.250	0.173	0.092	0.253	1.189	1.097	1.288
Sexual Health Clinic Consultation	0.000	-	-	1.000	-	-	0.000	-	-	1.000	-	-
**Access to Healthcare Professional**												
Phone	-0.059	-0.130	0.011	0.942	0.878	1.011	-0.077	-0.155	0.000	0.926	0.856	1.000
Instant Messenger	0.025	-0.045	0.095	1.025	0.956	1.099	-0.009	-0.086	0.068	0.991	0.918	1.071
Email	0.066	-0.003	0.136	1.069	0.997	1.145	0.001	-0.076	0.077	1.001	0.927	1.080
Face to Face	0.000	-	-	1.000	-	-	0.000	-	-	1.000	-	-
**How you get Antibiotics**												
Post to Home	-0.044	-0.118	0.030	0.957	0.889	1.030	0.053	-0.028	0.134	1.055	0.973	1.144
Post to Collection Point	-0.032	-0.104	0.040	0.968	0.901	1.041	0.070	-0.009	0.149	1.072	0.991	1.160
Collect from Pharmacy	0.067	-0.009	0.143	1.070	0.991	1.154	0.055	-0.029	0.139	1.056	0.971	1.149
Collect from Sexual Health Clinic	0.000	-	-	1.000	-	-	0.000	-	-	1.000	-	-

For peer review only

Table 3.5 - Previous STI Testing Subgroup Analysis Coefficients and Odds Ratios, including Respective 95% Confidence Interval

	Previous Test n=393						No Previous Test *n=790*					
	**Coefficient**	**Lower 95% CI**	**Upper 95% CI**	**Odds Ratio**	**Lower 95% CI**	**Upper 95% CI**	**Coefficient**	**Lower 95% CI**	**Upper 95% CI**	**Odds Ratio**	**Lower 95% CI**	**Upper 95% CI**
**How you Test**												
Self-Test	0.422	0.305	0.539	1.524	1.356	1.714	0.518	0.434	0.601	1.678	1.544	1.824
Post	0.164	0.047	0.280	1.178	1.048	1.323	0.382	0.298	0.466	1.465	1.347	1.594
Pharmacy	-0.056	-0.160	0.048	0.945	0.852	1.049	0.245	0.170	0.320	1.277	1.185	1.377
Education/ Work	-0.273	-0.377	-0.168	0.761	0.686	0.845	-0.150	-0.225	-0.074	0.861	0.798	0.928
GP Practice	-0.122	-0.229	-0.014	0.885	0.795	0.986	0.097	0.019	0.174	1.101	1.019	1.190
Sexual Health Clinic	0.000	-	-	1.000	-	-	0.000	-	-	1.000	-	-
**Time to Result**												
30 Mins	0.592	0.498	0.687	1.808	1.645	1.988	0.586	0.518	0.654	1.797	1.679	1.923
2 Hours	0.305	0.205	0.406	1.357	1.227	1.500	0.353	0.281	0.425	1.423	1.325	1.529
7 Days	0.000	-	-	1.000	-	-	0.000	-	-	1.000	-	-
14 Days	-0.125	-0.213	-0.037	0.882	0.808	0.963	-0.156	-0.219	-0.093	0.856	0.803	0.912
**Accuracy**												
2 in 100 False Negative	1.099	1.037	1.160	3.000	2.820	3.191	1.248	1.203	1.292	3.482	3.331	3.640
5 in 100 False Negative	0.000	-	-	1.000	-	-	0.000	-	-	1.000	-	-
**Consultation Method**												
Online Consultation	0.161	0.069	0.252	1.174	1.071	1.287	0.205	0.140	0.271	1.228	1.150	1.311
Pharmacy Consultation	0.094	0.003	0.184	1.098	1.003	1.203	0.168	0.103	0.233	1.183	1.108	1.262
GP Consultation	0.106	0.015	0.197	1.112	1.015	1.217	0.195	0.130	0.260	1.215	1.139	1.297
Sexual Health Clinic Consultation	0.000	-	-	1.000	-	-	0.000	-	-	1.000	-	-
**Access to Healthcare Professional**												
Phone	-0.054	-0.142	0.034	0.947	0.868	1.034	-0.048	-0.110	0.015	0.953	0.895	1.015
Instant Messenger	0.044	-0.042	0.131	1.045	0.958	1.140	0.025	-0.037	0.087	1.025	0.963	1.091
Email	0.048	-0.038	0.134	1.049	0.962	1.144	0.053	-0.009	0.114	1.054	0.991	1.121
Face to Face	0.000	-	-	1.000	-	-	0.000	-	-	1.000	-	-
**How you get Antibiotics**												
Post to Home	0.011	-0.081	0.102	1.011	0.922	1.107	0.020	-0.046	0.085	1.020	0.955	1.089
Post to Collection Point	0.034	-0.056	0.123	1.034	0.946	1.131	0.034	-0.029	0.098	1.035	0.971	1.103
Collect from Pharmacy	0.083	-0.012	0.177	1.086	0.988	1.194	0.075	0.008	0.143	1.078	1.008	1.153
Collect from Sexual Health Clinic	0.000	-	-	1.000	-	-	0.000	-	-	1.000	-	-

For peer review only

STROBE Statement—checklist of items that should be included in reports of observational studies

	Item No	Recommendation
**Title and abstract**	1	(*a*) Indicate the study's design with a commonly used term in the title or the abstract ✔ (page 1)
		(*b*) Provide in the abstract an informative and balanced summary of what was done and what was found ✔ (page 2)
**Introduction**		
Background/rationale	2	Explain the scientific background and rationale for the investigation being reported ✔ (page 4)
Objectives	3	State specific objectives, including any prespecified hypotheses ✔ (page 5)
**Methods**		
Study design	4	Present key elements of study design early in the paper ✔ (page 5-6)
Setting	5	Describe the setting, locations, and relevant dates, including periods of recruitment, exposure, follow-up, and data collection ✔ (page 7)
Participants	6	(*a*) *Cohort study*—Give the eligibility criteria, and the sources and methods of selection of participants. Describe methods of follow-up ✔ (page 7)<br>*Case-control study*—Give the eligibility criteria, and the sources and methods of case ascertainment and control selection. Give the rationale for the choice of cases and controls<br>*Cross-sectional study*—Give the eligibility criteria, and the sources and methods of selection of participants
		(*b*) *Cohort study*—For matched studies, give matching criteria and number of exposed and unexposed<br>*Case-control study*—For matched studies, give matching criteria and the number of controls per case
Variables	7	Clearly define all outcomes, exposures, predictors, potential confounders, and effect modifiers. Give diagnostic criteria, if applicable ✔ (page 8)
Data sources/ measurement	8*	For each variable of interest, give sources of data and details of methods of assessment (measurement). Describe comparability of assessment methods if there is more than one group ✔ (page 8)
Bias	9	Describe any efforts to address potential sources of bias ✔ (page 8)
Study size	10	Explain how the study size was arrived at ✔ (page 7)
Quantitative variables	11	Explain how quantitative variables were handled in the analyses. If applicable, describe which groupings were chosen and why ✔ (page 7)
Statistical methods	12	(*a*) Describe all statistical methods, including those used to control for confounding ✔ (page 7-8)
		(*b*) Describe any methods used to examine subgroups and interactions ✔ (page 8)
		(*c*) Explain how missing data were addressed
		(*d*) *Cohort study*—If applicable, explain how loss to follow-up was addressed<br>*Case-control study*—If applicable, explain how matching of cases and controls was addressed<br>*Cross-sectional study*—If applicable, describe analytical methods taking account of sampling strategy
		(*e*) Describe any sensitivity analyses
**Results**		
Participants	13*	(a) Report numbers of individuals at each stage of study—eg numbers potentially eligible,

For peer review only - http://bmjopen.bmj.com/site/about/guidelines.xhtml

		examined for eligibility, confirmed eligible, included in the study, completing follow-up, and analysed ✔ (page 8)
		(b) Give reasons for non-participation at each stage
		(c) Consider use of a flow diagram
Descriptive data	14*	(a) Give characteristics of study participants (eg demographic, clinical, social) and information on exposures and potential confounders ✔ (page 8 & Supplementary Information File 2)
		(b) Indicate number of participants with missing data for each variable of interest
		(c) *Cohort study*—Summarise follow-up time (eg, average and total amount)
Outcome data	15*	*Cohort study*—Report numbers of outcome events or summary measures over time ✔ (page 8-9)
		Case-control study—Report numbers in each exposure category, or summary measures of exposure
		Cross-sectional study—Report numbers of outcome events or summary measures
Main results	16	(*a*) Give unadjusted estimates and, if applicable, confounder-adjusted estimates and their precision (eg, 95% confidence interval). Make clear which confounders were adjusted for and why they were included ✔ (page 8-9)
		(*b*) Report category boundaries when continuous variables were categorized
		(*c*) If relevant, consider translating estimates of relative risk into absolute risk for a meaningful time period
Other analyses	17	Report other analyses done—eg analyses of subgroups and interactions, and sensitivity analyses ✔ (page 9)
**Discussion**		
Key results	18	Summarise key results with reference to study objectives ✔ (page 9-10)
Limitations	19	Discuss limitations of the study, taking into account sources of potential bias or imprecision. Discuss both direction and magnitude of any potential bias ✔ (page 10)
Interpretation	20	Give a cautious overall interpretation of results considering objectives, limitations, multiplicity of analyses, results from similar studies, and other relevant evidence ✔ (page 11)
Generalisability	21	Discuss the generalisability (external validity) of the study results ✔ (page 11)
**Other information**		
Funding	22	Give the source of funding and the role of the funders for the present study and, if applicable, for the original study on which the present article is based ✔ (page 12)

*Give information separately for cases and controls in case-control studies and, if applicable, for exposed and unexposed groups in cohort and cross-sectional studies.

For peer review only

BMJ Open

Young people's preferences for the use of emerging technologies for asymptomatic regular chlamydia testing and management: A discrete choice experiment in England

Journal:	*BMJ Open*
Manuscript ID	bmjopen-2018-023663.R2
Article Type:	Research
Date Submitted by the Author:	30-Oct-2018

Complete List of Authors:	Eaton, Sue; University of Warwick, Warwick Medical School<br>Biggerstaff, Deborah; University of Warwick Warwick Medical School<br>Petrou, Stavros; University of Warwick, Warwick Medical School<br>Osipenko, Leeza; National Institute for Health and Care Excellence, Scientific Advice<br>Gibbs, Jo; University College London, Research Department of Infection and Population Health<br>Estcourt, Claudia; Glasgow Caledonian University,<br>Sadiq, S. Tariq; St George's University of London, Institute for Infection and Immunity Research Institute<br>Szczepura, Ala; Coventry University, Enterprise & Innovation Group; University of Warwick Warwick Medical School

**Primary Subject Heading**:	Public health
Secondary Subject Heading:	Diagnostics, Health policy, Infectious diseases, Sexual health, Patient-centred medicine
Keywords:	sexually transmitted infections, chlamydia trachomatis, patient preferences, discrete choice experiment, online clinical care pathway, digital health

SCHOLARONE™
Manuscripts

Title: Young people's preferences for the use of emerging technologies for asymptomatic regular chlamydia testing and management: A discrete choice experiment in England

Authors:
Dr Sue Eaton, Warwick Medical School, University of Warwick, Coventry, UK.
Dr Deborah Biggerstaff, Warwick Medical School, University of Warwick, Coventry, UK.
Prof Stavros Petrou, Warwick Medical School, University of Warwick, Coventry, UK.
Dr Leeza Osipenko, National Institute for Health and Care Excellence, Scientific Advice, London.
Dr Jo Gibbs, Research Department of Infection & Population Health, University College London, London, UK.
Prof Claudia S Estcourt, Glasgow Caledonian University, Glasgow, UK.
Prof S Tariq Sadiq, Applied Diagnostic Research and Evaluation Unit, Institute for Infection and Immunity, St George's, University of London, London, UK.
Prof Ala Szczepura, Enterprise & Innovation Group, Coventry University, UK.

Corresponding author
Prof Ala Szczepura, BA DPhil (Oxon)
Enterprise & Innovation Group
Coventry University
Coventry CV1 2TT
UK
Email: ala.szczepura@coventry.ac.uk
Mob: +44 (0)7557 425 463

Key words: sexually transmitted infections, chlamydia trachomatis, patient preferences, discrete choice experiment, online clinical care pathway, digital health

For peer review only

ABSTRACT:

Objective: To undertake a comprehensive assessment of the strength of preferences among young people for attributes of emerging technologies for testing and treatment of asymptomatic chlamydia.

Design: Discrete choice experiment (DCE) with sequential mixed methods design. A staged approach to selection of attributes/ levels included two literature reviews, focus groups with young people aged 16-24 years (n = 21), experts' review (n=13), and narrative synthesis. Cognitive testing was undertaken to pilot and adapt the initial questionnaire. Online national panel was used for final DCE survey to maximise generalisability. Analysis of questionnaire responses used multinomial logit models and included validity checks.

Setting: England.

Participants: 1,230 young people aged 16-24 from a national online panel (completion rate 73%).

Outcome Measures: Odds Ratios (ORs) for service attributes in relation to reference levels.

Results: The strongest attribute influencing preferences was chlamydia test accuracy (OR 3.24, 95% CI 3.13–3.36), followed by time to result (OR 1.81, 95% CI 1.71–1.91). Respondents showed a preference for remote chlamydia testing options (self-testing, self-sampling and postal testing) over attendance at a testing location. For accessing treatment following a positive test result, there was a general preference for online (OR 1.21, 95% CI 1.15–1.28) versus traditional GP (OR 1.18, 95% CI 1.12–1.24) or pharmacy (OR 1.15, 95% CI 1.10–1.22) over clinic services. For accessing a healthcare professional and receipt of antibiotics, there was little difference in preferences between options.

Conclusions:
Both test accuracy and very short intervals between testing and results were important factors for young people when deciding whether to undergo a routine test for asymptomatic chlamydia, with test accuracy being more important. These findings should assist technology developers, policy makers, commissioners and service providers to optimise technology adoption in service redesign, although use of an online panel may limit generalisability of findings to other populations.

For peer review only

Strengths and Limitations of the Study:

- To our knowledge, this is the first large-scale DCE study to examine the preferences of a geographically representative national sample of young people for emerging technologies designed to improve screening for and treatment of asymptomatic chlamydia.
- Robust sequential methods were used to select final DCE attributes and levels, including two literature reviews, focus groups with young people, and review by expert groups.
- An online panel enabled access to the population targeted for screening, including young people with no personal experience of chlamydia testing and treatment.
- A limitation of this work is that the literature reviews inevitably identified more potential attributes than could be included in the DCE.
- The use of an online panel may limit generalisability of findings to the very small percentage (3%) of 15-24 year-olds who do not currently access the internet, and therefore over-represent the acceptability of online care.

For peer review only

INTRODUCTION:

Chlamydia is the most commonly reported sexually transmitted infection (STI) in England, with young people aged 15-24 accounting for 63% of diagnoses in 2016.[1] The estimated annual cost of chlamydia treatment in 2015 was £249.8 million.[2] Undetected infections and re-infections can lead to significant adverse health consequences, such as pelvic inflammatory disease, ectopic pregnancy and infertility, which have an impact on National Health Service (NHS) costs and health-related quality of life.[3] A National Chlamydia Screening Programme (NCSP) was therefore rolled out in England for 16-24 year olds between 2003 and 2008.[4] However, despite recent evidence of a reduction in chlamydia prevalence in England for for 2000 to 2015, concurrent with large-scale population testing[5], and the fact that uncomplicated infection is easy to treat with oral antibiotics, uptake of screening for chlamydia remains low.[6] Worryingly, there has also been a decline over the last four years in the number of local authorities achieving the public health outcomes framework indicator of 2,300 diagnoses per 100,000 population.[1] Barriers identified for young people accessing STI testing and treatment services include tangible service attributes, such as location of service, and personal or behavioural factors such as the stigma associated with attendance, embarrassment, fear of being recognised and privacy concerns.[7-9]

A range of options are currently available for young people to access asymptomatic chlamydia testing and treatment services. For testing, options include attending a genito-urinary medicine (GUM) clinic, testing via primary care, e.g. pharmacies/general practitioners (GPs), or via internet testing services where a number of websites offer free self-sampling kits online with samples sent to laboratories for analysis and results communicated directly to patients. For individuals who screen positive, options for accessing antibiotic treatment include attending a GUM/sexual health clinic, GP practice or pharmacy. Even though a national screening programme exists in England, large geographical variations exist in testing coverage, from 16% of young people tested for chlamydia in the West Midlands region to 27% in London, and in the proportion of positive cases treated.[1 10]

For many health services, digital technology is now widely regarded by policy makers as one approach to improve access and reduce costs.[11] For chlamydia testing and treatment, a range of digital options are available, and more technologies are likely to enter the market within the next 3-5 years.[12] These include point-of-care tests[13], online postal self-sampling, eSexual health clinics[14], apps and non-face-to-face consultation methods[15 16], and self-tests networked through mobile phones[17]. Such innovations provide an opportunity to redesign current chlamydia screening services with the aim of improving testing and treatment uptake. However, such service redesign should be based on a sound understanding of the preferences of young people as service-users for specific attributes of such services.

Research specifically measuring young people's preferences nationally for chlamydia testing and treatment service options is lacking. Even for STI testing services generally, relatively few studies have used a discrete choice experiment (DCE) design to assess preferences in the UK population.[18-21] In comparison with other preference elicitation methods, a DCE can quantify the relative importance of different attributes that characterise a new or existing product and/or service, identifying which attributes people prioritise and which they may be willing to trade with the view to maximising their utility.[22] A DCE requires respondents to choose

For peer review only - http://bmjopen.bmj.com/site/about/guidelines.xhtml

between competing scenarios, e.g. service options, described in terms of a particular attribute (e.g. time to test result) and a range of levels (e.g. 30min to 14 days) and to compare these against an alternative scenario. DCE studies are very useful because they allow a direct assessment of relative preferences for various existing and hypothetical new service configurations or treatment approaches.[23]

The aim of the present study was to undertake a comprehensive assessment of the preferences of young people, targeted by the National Chlamydia Screening Programme, for emerging technology options for testing and treatment in the context of a "check-up test" where remote care could be medically appropriate.[14] To our knowledge, no previous study has attempted to disentangle strength of preference for attributes associated with new and emerging options for chlamydia screening (such as self-testing) or treatment (such as online prescription). Because STI services are "open access" and not subject to gatekeeping by referral from a clinician, the impact of such disruptive innovations will be directly dependent on population preferences. Early insight into attributes that could influence whether individuals are more likely to use a new testing or treatment pathway should therefore be helpful in informing product development and pathway redesign for future chlamydia screening service models.[24 25]

METHODS:

This study was conducted using an exploratory sequential mixed methods design, adopting recommended stages for undertaking a DCE[26], as shown in Figure 1. In selecting methods to design the questionnaire and conduct the DCE, reference was made to the International Society for Pharmacoeconomics and Outcomes Research good practice checklist for conjoint analysis.[27]

Patient and public involvement: Young people (with and without experience of STI services) participated in focus groups to inform priorities included in the DCE; in cognitive testing to finalise questionnaire design; and in questionnaire completion to identify preferences, as described in the Acknowledgements.

Selection of Attributes and Levels

The attributes and associated levels were determined using a four-stage approach.[28] More detailed information is provided in Supplementary Information File 1.

Literature Reviews

Two literature reviews were undertaken to produce a comprehensive list of potential themes and factors that might influence young people's choices. These included: (i) a systematic review of the use of stated preference studies for STI testing and treatment (PROSPERO Reg: CRD42014014862); and (ii) a scoping review of other research exploring preferences and acceptability of STI testing and treatment services.

Focus Groups

Focus groups were run with young people aged 16-24 years (4 groups, 3-7 per group; total n=21) to identify which themes and factors young people consider important when choosing

For peer review only - http://bmjopen.bmj.com/site/about/guidelines.xhtml

to test for STIs. Convenience sampling was used to identify participants. Focus group topic guides incorporated typical vignettes of situations individuals might encounter. Sessions were recorded, transcribed and thematic analysis was performed to produce a list of potential attributes and levels.[29]

Expert Groups

Four expert groups were convened (n=13), including a range of professionals with expert knowledge of the service and technology context. Expert groups were asked to review the focus group findings in terms of clinical feasibility and practicality.[26]

Narrative Synthesis

Narrative synthesis[30] enabled outputs from the three prior stages to be synthesised for each potential attribute. This approach was adopted as it offered a clear approach to synthesis based on the following stages: (i) identification of a checklist of properties against which attributes can be considered; (ii) tabulation against the checklist; and (iii) conceptual mapping and triangulation against the checklist. The final synthesis highlighted a tension between young people's desire to be tested for a wide range of STIs irrespective of risk, versus clinical guidelines for selective testing of STIs based on population group prevalence and risk. The range of STIs presented in the DCE was consequently limited to Chlamydia. Because focus group findings indicated difficulty in understanding several dimensions of test performance, test accuracy was expressed in terms of the likelihood of a false negative result.

Questionnaire Design and Piloting

A generic pairwise choice with opt out question was selected for the questionnaire design. Respondents were presented with a series of choice sets for which there were 3 responses: 'option A', 'option B' or 'I would not test'. A sample choice set is illustrated in Figure 2. The questionnaire adopted a main effects design using full profiles (all attributes included in the study). Whilst some DCEs do include an attribute on economic costs, DCEs exploring preferences for STI testing services in England have generally excluded cost since the NHS provides treatment 'free at the point of delivery'[20 21] A cost attribute was therefore not included.

The questionnaire was developed using SAS 9.4 software to ensure that the design was D-efficient.[27] A full factorial design was ruled out in favour of a fractional factorial design because a full factorial design would have contained 3,072 possible alternatives, which would have been unmanageable in practice for individuals to complete or for a blocked questionnaire format to handle[31]. The smallest 100% efficient design that could be created included 48 choice sets. These were blocked (halved) into two questionnaires each with 24 choice sets using SAS JMP Pro 9.2.0. Choice set 1 was repeated as choice set 25 in each questionnaire to provide an internal validity check.

Three rounds of cognitive testing (n=9) were undertaken to check respondents' comprehension of information when making choices. Cognitive testing, undertaken based on two questionnaires of 24 sets, confirmed that a study based on two such questionnaires was acceptable to participants. Some modifications were made to levels where reasons for choice selection demonstrated that one level (e.g. 8 in 100 false negative) dominated the reason for selection. Cognitive testing also identified that implausible combinations (e.g. a postal test

For peer review only - http://bmjopen.bmj.com/site/about/guidelines.xhtml

providing a result in two hours) impacted on completion of the task, whilst unlikely (but feasible) combinations did not. Only implausible combinations were therefore excluded from the choice sets. The D-Efficiency of the design[27] calculated by SAS JMP Pro 9.2.0, prior to the removal of implausible combinations, was 98.06. The final combination selected for the questionnaire was the one which created no duplicate choices and which provided an equal balance of the number of choice sets containing overlap between questionnaires.

Respondents were asked to provide socio-demographic information, including their age, gender, ethnicity, region of residence, sexual preference, relationship status and whether they had previously been tested for an STI. An introduction to the DCE questionnaire provided background information and an explanation of the attributes and levels. The introduction was included in the cognitive testing rounds to check comprehension within the target age range. As a result, the text was modified and diagrams added to illustrate the chlamydia testing pathways. The final phase of testing demonstrated that the number of choice sets was acceptable to respondents. The use of an online panel also provided completion time data to support the internal validity checks, and enabled an accurate record of time taken to complete the survey.

Final Attributes and Levels Selected

The six attributes and 24 levels selected for inclusion in the final DCE questionnaire are shown in table 1. More detailed information used to explain these attributes and levels is provided in Supplementary Information File 2 (pages 3-7).

Participants and Recruitment Procedure

Participants were drawn from the general population rather than from health care settings, thereby providing access to young people aged 16-24 years who had not previously been tested for an STI. An online national panel (YouthSight)[32] was used to maximise geographical reach and generalisability. Ex-ante sub-group analysis was planned by three age bands (16-18, 19-21, 22-24) and by gender (male, female). Where sub-group analysis is planned, it is recommended that there should be a minimum of 200 respondents in each sub-group, so a sample size of 1,200 was required in our study, 600 per questionnaire.[33] Consent was obtained online prior to questionnaire completion. Participants were offered a small reimbursement of one point (equivalent to £1) for completion of a survey of up to 20 minutes in length. Points could be exchanged for shopping vouchers.

Statistical Analyses

The multinomial logit (MNL) model developed by McFadden was used for analysing responses; this is recognised as the convention for three-response choice set studies ('option A', 'option B' or 'I would not test').[26 34] Analysis utilised STATA13 SE with the method and code outlined by Ryan *et al*[23] Analyses presented odds ratios (ORs), 95% confidence interval (CIs) and coefficients for each attribute level. Variables within the model were all treated as categorical variables for the analysis. Attribute levels were specified using dummy coding, the preferred form of coding where ORs are to be calculated. Within the model, the levels that were dropped to form the reference levels reflected those aligned to a 'typical' sexual health clinic pathway (summarised in table 2). To test the internal validity of questionnaire responses, analyses compared full results against: (i) removal of responses where participants did not answer the repeated choice set consistently; (ii) removal of any respondents who took

For peer review only - http://bmjopen.bmj.com/site/about/guidelines.xhtml

less than the minimum time (five minutes) observed in cognitive testing to complete the questionnaire; and (iii) removal of responses containing the opt out question data. Further tests for internal consistency and rationality were not included, since excluding responses on this basis may be viewed as an inappropriate imposition of rationality.[35]

Demographic characteristics (gender, age and ethnicity) of respondents were compared with national Census data.[36] The influence of patient level characteristics (age, sex and STI testing history (yes/no)) on the likelihood of not choosing to test was examined. In addition to *ex-ante* planned sub-group analyses, if sufficient responses were received analysis was also planned to compare: (i) respondents who had, or had not, previously tested for an STI, and (ii) those who indicated their relationship status as 'single' versus those in a sexual relationship with one person.

Trade-off between accuracy and time to result was examined by considering the probability of uptake for tests with characteristics at the opposite ends of the spectrum i.e. 'lower accuracy (5% false negatives), faster time to result (30 min)' and 'higher accuracy (2% false negatives), longer time to result (14 days)'.

RESULTS:

In total, 1,230 fully completed questionnaires were received, the platform analytics showed that 460 people had opened the questionnaire but did not complete it, providing a completion rate of 73%. No further information was available on the demographics of the 460 non-responders nor any information on the point at which they chose to exit the survey, so these people could not be included in our analyses. Time to complete the 25 choice sets ranged from one minute 19 seconds to 30 minutes 19 seconds with a median time to completion of seven minutes 51 seconds. Demographic characteristics of respondents are summarised in the Supplementary Information File 3 (Part 1). Comparing the DCE sample to the characteristics of 16-24 year olds in England indicates that gender, age and ethnicity are broadly in line with national population demographics, with a slight under-representation of the 16-18 year age group. In terms of the geographical residence of participants, the sample was broadly in line with the geographical distribution of 16-24 year olds identified in the ONS Mid-Year Population Estimates 2015[37], with the exception of a lower proportion of respondents from the East of England.

Internal validity checks showed that, in comparison to the full dataset, all checks yielded very similar ORs with no change in the order of the strength of preference for a level within each attribute. Results of the internal validity checks are included in the Supplementary Information File 3 (Part 2).

OR values for the full dataset and subgroups analysed are presented in table 3 with reference levels (1.00). Analysis of the trade-off between accuracy and time to result is presented in table 4. More detailed DCE results, including 95% CIs as well as OR values plus coefficients for each attribute level for all sub-group analysis are presented in Supplementary Information File 3 (Part 3).

In table 3, looking across all attributes and levels for the whole population, participants expressed the strongest preference for attribute 1 - accuracy of test result (OR 3.242, 95% CI

For peer review only - http://bmjopen.bmj.com/site/about/guidelines.xhtml

3.130–3.359). There are also differences in the strength of preference between males and females for this attribute (OR 2.951, 95% CI 2.807–3.101 and 3.570, 95% CI 3.396–3.753, respectively), and between those who had previously tested or not tested (OR 3.000, 95% CI 2.820–3.191 and 3.482, 95% CI 3.331–3.640, respectively). Time to result was the attribute showing the next strongest preference across all subgroups (OR 1.806, 95% CI 1.711–1.906). These results are consistent with the logical expectation that people will prefer higher accuracy and a shorter waiting time. Looking specifically at the trade-off between accuracy and time to result, table 4 indicates that participants are willing to wait noticeably longer in order to have a test result with a lower chance of a false negative result.

When considering how to test, all subgroups demonstrated a preference for self-testing (OR 1.618, 95% CI 1.514–1.729) over attendance at a sexual health clinic. Testing via an outreach service in an educational/ work setting was found to be the least preferred option (OR 0.821, 95% CI 0.773–0.872). Respondents showed a consistent strength of preference for those options that do not involve direct contact with healthcare professionals (self-testing and postal self-sampling), compared with those that do. For consultation and treatment following a positive test result, there was a preference for non-sexual health clinic pathways with online consultation to access treatment (OR 1.212, 95% CI 1.150–1.277), treatment via general practice (OR 1.183, 95% CI 1.123–1.246) and treatment via pharmacy (OR 1.158, 95% CI 1.100–1.220) preferred in the full dataset. At subgroup level (age, gender, previous testing history and relationship status), more variation was found in the preference order for this attribute, with the exception of the sexual health clinic which was consistently the least preferred option across all subgroups (see table 3 and Supplementary Information File 3, Part 3). Finally, the full dataset shows that, if someone wants to access a healthcare professional, there was no statistically significant preference for instant messenger or email access compared with accessing the professional face-to-face. Telephone access to a healthcare professional was the least preferred access option (OR 0.949, 95% CI 0.903–0.998), and the only statistically significant result when compared with face-to-face access (the reference level). There was, similarly, no statistically significant difference in preferences for how young people might access antibiotics apart from a slight preference for the pharmacy versus sexual health clinic (OR 1.075, 95% CI 1.018–1.134).

DISCUSSION:

Our findings indicate that, based on the levels included in the DCE questionnaire, young people are willing to wait in order to have a chlamydia screening test result with a lower chance of a false negative result. The conclusion for new test developers is that time to result is less important than accuracy, and that test users are unlikely to prefer a point-of-care or self-screening test with lower accuracy than the tests currently available to them. There was a strong preference for remote access to testing, consultation, and antibiotic prescriptions, although for accessing a health professional there was no preference between online and face-to-face methods. This suggests a remote online pathway is acceptable to young people, *as long as* test performance remains equivalent.

In the various hypothetical situations presented, respondents showed a preference for chlamydia self-testing, self-sampling and postal testing over attendance at a testing location. For accessing treatment, a general preference was exhibited for online versus traditional GP

For peer review only - http://bmjopen.bmj.com/site/about/guidelines.xhtml

or pharmacy services over clinic services. For receipt of antibiotics, there was little difference in preferences. We were also able to identify which attributes people may be willing to trade to maximise their utility. Looking at the trade-off between accuracy and time to result, we found that young people are willing to wait noticeably longer in order to have a test result with a lower chance of a false negative result, reinforcing the need for test equivalence.

The strengths of this DCE study include, firstly, the robust methods employed to select attributes and levels, which aligned with the recommendations of Coast *et al*, ensuring attributes are "manipulable in policy".[38] Secondly, the fact that participants were drawn from the general population targeted by the National Chlamydia Screening Programme, rather than from healthcare settings. This enabled us to access a demographically and geographically representative national sample of young people, including those who have had no previous contact with STI services. Finally, the use of an online panel enabled large scale data to be collected at a reasonable cost and allowed validity checks that would not otherwise have been possible with postal questionnaire responses. The large sample size also allows comparison between several subgroups to explore differences based on age, gender, previous testing history and relationship category.

However, the study does have a few limitations. The first relates to the selection of attributes and levels. Whilst the selection process employed was very rigorous, this cannot detract from the fact that further attributes were identified which might impact on individuals' choices. To mitigate against this, where such an attribute was excluded, information was provided in the survey background section to minimise respondents forming their own views on the impact of this attribute on the pathway.

Use of an online panel also provided accurate records of the time taken to complete the survey, and permitted additional validity checks which would not otherwise have been possible with a written questionnaire. On the other hand, use of an online panel excluded young people who do not have access to the internet, thereby potentially over-representing the acceptability of online care. However, given the extremely high proportion (97%) of 15-24 year-olds accessing the internet daily via a mobile device[39] and owning a smartphone[40], it is evident that the vast majority of the target population could access online care pathways if they choose to do so. The question of whether these young people have the degree of digital and health literacy needed for online testing and treatment was not explored in this study. Finally, information was unavailable on young people who opened but decided not to complete the questionnaire, so systematic comparison with those who responded was not possible.

Behavioural factors, such as embarrassment[7], stigma[41] and privacy and anonymity concerns[42 43] are known to influence uptake of sexual health services, as well as structural factors such as convenience and perceived barriers to access[8]; many of these may be lower for a non-face-to-face service. Balanced against this, access to online testing and treatment is valued less strongly than attributes such as test accuracy and time to result. In individual cases a young person's preference for remote versus face-to-face testing and treatment might differ.

Finally, it is difficult to compare the present results to other published DCEs because of key differences in perspective. Miners *et al* (sample n=3,358)[20] and Llewellyn *et al* (sample

For peer review only - http://bmjopen.bmj.com/site/about/guidelines.xhtml

n=233)[21] both only focussed on preferences within existing traditional service delivery models, and did not incorporate any hypothetical future scenarios (e.g. point-of-care testing, self-testing or treatment via eHealth/mHealth solutions). Two other DCE studies, which did consider self-sampling at home for chlamydia screening[18 19], with sample sizes 174 and 126 respectively, both described sending the sample to a laboratory for analysis rather than a self-test. One of these studies[19] did identify a stronger preference for attendance at a family planning clinic, rather than self-sampling, but since participants were recruited from the waiting room of such a clinic they cannot be considered representative of the general population targeted by the NCSP. A fifth study, which examined preferences for point-of-care testing, only surveyed clinicians undertaking STI testing (sample n=218), thus excluding the population actually targeted for chlamydia screening.[44] The only stated preference study that has considered patients' preferences for STI self-testing focussed on HIV (sample n=365).[45] The authors reported results in line with our findings, with respondents exhibiting a preference for tests which are accurate, timely and private/ anonymous. However, this study was undertaken in 2002 prior to smartphones and at a time when self-testing for HIV was still under development. Importantly for screening tests, only one DCE study to date[21] has sought to include non-service users (i.e. populations with no experience of STI testing). The authors identified a preference for testing for all STIs, in settings with healthcare professionals with specialist knowledge present, and for receipt of negative as well as positive results. This study used a convenience sample of 233 students from two universities which was unlikely to be geographically or socio-economically representative. All other studies have drawn their DCE samples from people who were either current service users, or attendees for other, linked services. Use of such samples represents a significant short-coming when considering the introduction of new technologies for asymptomatic chlamydia testing and management. Most sample sizes were also smaller than in the present study.

A number of questions remain which our research does not address. Firstly, recognising that the range of STIs included is an important consideration for young people in choosing to test[20 21], further research is required to understand this better in the context of potential new screening pathways which incorporate other STIs (e.g. gonorrhoea). Secondly, our study highlights a number of methodological considerations where there is an absence of consensus that may warrant further exploration to improve consistency. These include the number of choice sets to include in a DCE and the use of repeated choice sets as an internal validity measure. Finally, given that cost was not included as an attribute in this study, it is not possible to provide an indication of willingness to pay, or the monetary benefits of potential service changes, from this DCE.[46]

The present study is, to our knowledge, the first to present a large scale, quantitative analysis of young people's preferences for attributes of potential new pathways to deliver testing and treatment of asymptomatic chlamydia, based on a nationally representative population. The DCE methodology applied also produced a measure of the relative strength of preference between different attributes and levels, and potential trade-offs. This can provide useful evidence to technology developers, policy makers, commissioners and service providers. In particular, it provides a first insight into preferences for the type of technologies currently under development, and those which might be available for use in the near future, compared to the features of existing products and services. This can indicate how young people may respond to changes in pathways and to the introduction of new technologies.

For peer review only - http://bmjopen.bmj.com/site/about/guidelines.xhtml

Within the context of current UK sexual health policy and commissioning of sexual health services[12], this DCE provides supportive evidence for the policy direction of remote chlamydia testing and treatment. However, whilst young people overall expressed a stronger preference for attributes such as self-test, online consultation etc, a small proportion still preferred existing pathways. This suggests that, in order to maximise benefit, face-to-face services should continue to be available in addition to any online screening and treatment service. This will ensure that services are inclusive and accessible irrespective of digital/ health literacy, while recognising that people's needs and preferences may change depending on their personal circumstances.

Acknowledgements
We thank Dr Joshua Pink, National Institute for Health and Care Excellence (NICE), Manchester, UK for providing specialist advice on DCE design and analysis. We would also like to thank academics in the Studies in Adolescent Sexual Health (SASH) Group at Coventry University who provided advice on how to access the young people who participated in focus groups; and YouthSight for their expertise in converting the questionnaire into an online format. Finally, the authors wish to thank all the young people who took the time to participate in the focus groups, cognitive testing and complete a detailed questionnaire describing their preferences, and the experts who contributed their views in the expert groups.

Contributors:
SE conceived and undertook the study as part of her PhD research. AS was lead supervisor and takes responsibility for the integrity of the data and the accuracy of the data analysis. SP, LO and AS oversaw the DCE design and analysis; DB guided the qualitative research. CSE and JG provided clinical input into the development of the DCE materials and assisted with interpretation of findings. SE, AS, SP, LO and DB wrote the first draft of the article. STS (Principal Investigator), AS and CSE were applicants on the eSTI2 Consortium grant, wrote the initial protocol and obtained funding. All authors contributed to and agreed the final version of the manuscript.

Funding:
The Electronic Self-Testing Instruments for Sexually Transmitted Infection Control (eSTI^2) Consortium (which funded SE's studentship and also AS, JG, CSE and STS) was funded under the UKCRC Translational Infection Research (TIR) Initiative supported by the Medical Research Council (Grant Number G0901608) with contributions to the Grant from the Biotechnology and Biological Sciences Research Council, the National Institute for Health Research on behalf of the Department of Health, the Chief Scientist Office of the Scottish Government Health Directorates and the Wellcome Trust.

The funding body had no role in the design of the study, analyses, interpretation, or decision to submit the manuscript for publication.

Competing Interests:
None Declared

For peer review only - http://bmjopen.bmj.com/site/about/guidelines.xhtml

Ethics Approval
Ethical approval was granted by the Biomedical & Scientific Research Ethics Committee at the University of Warwick (ref: REGO-2015-1647).

Data Sharing Statement:
Requests for access to data should be addressed to the corresponding author

References

1. Public Health England. Sexually Transmitted Infections and Chlamydia Screening in England, 2016. 2017. https://assets.publishing.service.gov.uk/government/uploads/system/uploads/attachment_data/file/617025/Health_Protection_Report_STIs_NCSP_2017.pdf
2. Development Economics. Unprotected Nation - The Financial and Economic Impacts of Restricted Contraceptive and Sexual Health Services. 2013. https://www.fpa.org.uk/sites/default/files/unprotected-nation-sexual-health-full-report.pdf.
3. Public Health England. Opportunistic Chlamydia Screening of Young Adults in England: An Evidence Summary. 2014. https://assets.publishing.service.gov.uk/government/uploads/system/uploads/attachment_data/file/497371/Opportunistic_Chlamydia_Screening_Evidence_Summary_April_2014.pdf.
4. Public Health England. Guidance: NCSP: programme overview. 2013. https://www.gov.uk/government/publications/ncsp-programme-overview/ncsp-programme-overview.
5. Lewis J, White PJ. Changes in chlamydia prevalence and duration of infection estimated from testing and diagnosis rates in England: a model-based analysis using surveillance data, 2000-15. Lancet Public Health 2018;**3**(6):e271-e78.
6. Public Health England. Health protection report, volume 10, issue 22. 2016. https://www.gov.uk/government/publications/health-protection-report-volume-10-2016/hpr-volume-10-issue-22-news-8-july
7. Booth A R, Harris P R, Goyder E, et al. Beliefs about chlamydia testing amongst young people living in relatively deprived areas. J Public Helath (Oxf) 2013;**35**:213-22.
8. Friedman AL, Bloodgood B. Exploring the feasibility of alternative STD-testing venues and results delivery channels for a national screening campaign. Health Promot Pract 2013;**14**(1):96-104.
9. Balfe M, Brugha R. What prompts young adults in Ireland to attend health services for STI testing? BMC Public Health 2009;**9**:311.
10. NCSP. NCSP Scorecard 2011-12. 2012. http://www.chlamydiascreening.nhs.uk/ps/data.asp.
11. National Information Board & Department of Health. Personalised Health and Care 2020 - Using Data and Technology to Transform Outcomes for Patients and Citizens, A Framework for Action. 2014. https://www.gov.uk/government/uploads/system/uploads/attachment_data/file/384650/NIB_Report.pdf.

For peer review only

12. Eaton S, Osipenko L, Petrou S, et al. National Telemedicine and Telehealth Policy Context in England and Introduction of Digital Innovations for Detection and Treatment of Sexually Transmitted Infections. SM J Clin Med 2017;**3**(1).
13. Harding-Esch EM, Nori AV, Hegazi A, et al. Impact of deploying multiple point-of-care tests with a 'sample first' approach on a sexual health clinical care pathway. A service evaluation. Sex Transm Infect 2017;**93**(6):424-29.
14. Estcourt C GJ, Sutcliffe L, Gkatzidou V, Tickle L, Hone K, Aicken C, Lowndes C, Harding-Esch E, Sue Eaton S, Oakeshott P, Szczepura A, Ashcroft R, Copas A, Nettleship A, Sadiq SJ, Sonnenberg P,. The eSexual Health Clinic System for Sexually Transmitted Infection Management, Prevention and Control: Exploratory studies demonstrating safety, feasibility and public health utility. The Lancet Public Health 2017.
15. Lloyds Pharmacy. Online Doctor. 2018. https://onlinedoctor.lloydspharmacy.com.
16. City Health Care Partnership CIC. Sexual & Reproductive Healthcare Services. 2018. www.sexualhealthvirtualclinic.co.uk.
17. UK Research and Innovation. Enabling and translating advances in diagnostic and communication technologies to reduce the burden of STIs. 2018. http://gtr.ukri.org/projects?ref=G0901608.
18. Ryan M, Watson V. Comparing welfare estimates from payment card contingent valuation and discrete choice experiments. Health Econ 2009;**18**(4):389-401.
19. Watson V, Ryan M, Watson E. Valuing experience factors in the provision of Chlamydia screening: an application to women attending the family planning clinic. Value Health 2009;**12**(4):621-3.
20. Miners A, Llewellyn C, Pollard A, et al. Assessing user preferences for sexually transmitted infection testing services: a discrete choice experiment. Sex Transm Infect 2012;**88**(7):510-6.
21. Llewellyn CD, Sakal C, Lagarde M, et al. Testing for sexually transmitted infections among students: a discrete choice experiment of service preferences. BMJ open 2013;**3**(10):e003240.
22. Lancsar E, Louviere J. Conducting discrete choice experiments to inform healthcare decision making: a user's guide. Pharmacoeconomics 2008;**26**(8):661-77.
23. Ryan M, Watson V, Krucien N, et al. Using Discrete Choice Experiments in Health Economics: Theoretical and Practical Issues. University of Aberdeen, UK: Health Economics Research Unit, 2014.
24. Hall J, Viney R, Haas M, et al. Using stated preference discrete choice modeling to evaluate health care programs. Journal of Business Research 2004;**57**:1026-32.
25. Ryan M. Discrete choice experiments in health care. BMJ 2004;**328**(7436):360-1.
26. Ryan M, Gerard K, Amaya-Amaya ME. *Using Discrete Choice Experiments to Value Health and Health Care.* Dordrecht, The Netherlands: Springer, 2008.
27. Bridges JF, Hauber AB, Marshall D, et al. Conjoint analysis applications in health--a checklist: a report of the ISPOR Good Research Practices for Conjoint Analysis Task Force. Value Health 2011;**14**(4):403-13.
28. Eaton S. Integrating Novel Digital Technology for the Testing & Treatment of Chlamydia into Mainstream Sexual Health Services in England. University of Warwick, 2017.
29. Silverman D. *Interpreting Qualitative Data, London, Sage Publications Ltd.* London: Sage Publications Ltd., 2014.
30. Pope C, Mays N, J. P. *Synthesising Qualitative and*

For peer review only - http://bmjopen.bmj.com/site/about/guidelines.xhtml

Quantitative Health Evidence A Guide to Methods. Maidenhead, England: Open University Press, 2007.
31. Vanniyasingam T, Daly C, Jin X, et al. Investigating the impact of design characteristics on statistical efficiency within discrete choice experiments: A systematic survey. Contemp Clin Trials Commun 2018;**10**:17-28.
32. Youthsight. YouthSight Panel Services. 2018. http://www.youthsight.com/expertise/panel-services/.
33. Orme B. *Getting Started with Conjoint Analysis: Strategies for Product Design and Pricing Research, Madison, Wisconsin, Research Publishers LLC.* Madison, USA: Research Publishers LLC, 2010.
34. Louviere J, Hensher DA, Swait JD. *Stated Choice Methods Analysis and Application.* Cambridge, England.: Cambridge University Press, 2003.
35. Clark MD, Determann D, Petrou S, et al. Discrete choice experiments in health economics: a review of the literature. Pharmacoeconomics 2014;**32**(9):883-902.
36. Office for National Statistics. 2011 Census, Key Statistics for Local Authorities in England and Wales . 2012. http://webarchive.nationalarchives.gov.uk/20160107112030/http://www.ons.gov.uk/ons/publications/re-reference-tables.html?edition=tcm%3A77-286262.
37. Office for National Statistics. Population Estimates for UK, England and Wales, Scotland and Northern Ireland: mid-2015. . 2016. https://www.ons.gov.uk/peoplepopulationandcommunity/populationandmigration/populationestimates/bulletins/annualmidyearpopulationestimates/mid2015.
38. Coast J, Al-Janabi H, Sutton EJ, et al. Using qualitative methods for attribute development for discrete choice experiments: issues and recommendations. Health Econ 2012;**21**(6):730-41.
39. Office for National Statistics. Internet access – households and individuals: 2016. 2016. https://www.ons.gov.uk/peoplepopulationandcommunity/householdcharacteristics/homeinternetandsocialmediausage/bulletins/internetaccesshouseholdsandindividuals/2016.
40. Ipsos Connect. Technology Tracker. Quarterly Release: Q2 2016. 2016. https://www.ipsos.com/sites/default/files/2016-10/Ipsos_Tech_Tracker_Q2_2016.pdf.
41. Balfe M, R. B. What concerns do young adults in Ireland have about attending health services for STD testing? Deviant Behav 2011;**32**:320-50.
42. Baraitser P, Brown KC, Gleisner Z, et al. 'Do it yourself' sexual health care: the user experience. Sex Health 2011;**8**(1):23-9.
43. Lorimer K, McDaid L. Young men's views toward the barriers and facilitators of Internet-based Chlamydia trachomatis screening: qualitative study. Journal of medical Internet research 2013;**15**(12):e265.
44. Hsieh YH, Gaydos CA, Hogan MT, et al. What qualities are most important to making a point of care test desirable for clinicians and others offering sexually transmitted infection testing? PloS one 2011;**6**(4):e19263.
45. Phillips KA, Maddala T, Johnson FR. Measuring preferences for health care interventions using conjoint analysis: an application to HIV testing. Health Serv Res 2002;**37**(6):1681-705.
46. de Bekker-Grob EW, Ryan M, Gerard K. Discrete choice experiments in health economics: a review of the literature. Health Econ 2012;**21**(2):145-72.

For peer review only - http://bmjopen.bmj.com/site/about/guidelines.xhtml

For peer review only

For peer review only - http://bmjopen.bmj.com/site/about/guidelines.xhtml

FIGURE LEGENDS

Figure 1 – Stages in Conducting a Discrete Choice Experiment

Figure 2 – Sample Choice Set from the Discrete Choice Experiment

For peer review only

Table 1 – Summary of Attributes and Levels Included in the Study
*Full descriptions provided in **Supplementary Information File 2** (pages 3-7).*

Attribute / Level	A1. How you Test	A2. Time to Result	A3. Accuracy	A4. Consultation Method	A5. Access to HCP	A6. How you get Antibiotics
L1.	Self-test	30 mins	2 in 100 (False Negative)	Online Consultation	Phone	Post to Home
L2.	Self-Sample & Post	2 Hours	5 in 100 (False Negative)	Pharmacy	IM	Post to Collection Point
L3.	Self- Sample & Pharmacy	7 Days		GP	Email	Collect from Pharmacy
L4.	Self-Sample & Education/ Work	14 Days		Sexual Health Clinic	Face to Face	Collect from Sexual Health Clinic
L5.	GP Practice – Sample taken by healthcare professional					
L6.	Sexual Health Clinic – Sample taken by healthcare professional					

only

Table 2 – Dummy Coding Reference Level

Attribute	Reference Level
How you test	Sexual Health Clinic
Time to result	7 Days
Accuracy	5 in 100 (False Negative)
Consultation method	Sexual Health Clinic
Access to HCP	Face to Face
How you get antibiotics	Collect from Sexual Health Clinic

For peer review only

Table 3 – Overview Odds Ratios for all Sub-Groups Included within the Analysis.

For further information regarding the explanation of attributes and levels to young people in the questionnaire please see ***Supplementary File 2****.*
Odds Ratio values not statistically significant are shaded blue.
95% Confidence Intervals provided in ***Supplementary File 3****.*

	Full Dataset	Male	Female	16-18	19-21	22-24	Single	One Partner	Prev Test	No Prev Test
**Attribute & Levels**	**1,230**	**607**	**623**	**415**	**406**	**409**	**615**	**512**	**393**	**790**
	**Odds Ratio**	**Odds Ratio**	**Odds Ratio**	**Odds Ratio**	**Odds Ratio**	**Odds Ratio**	**Odds Ratio**	**Odds Ratio**	**Odds Ratio**	**Odds Ratio**
**1. How you Test**										
Self-Test	1.618	1.549	1.693	1.664	1.346	1.895	1.632	1.712	1.524	1.678
Self-Sample and post to a laboratory for analysis	1.358	1.308	1.411	1.477	1.138	1.487	1.380	1.380	1.178	1.465
Self-Sample and take to a pharmacy for analysis	1.155	1.131	1.180	1.270	1.021	1.185	1.220	1.109	0.945	1.277
Self-Sample and take to place of education/work for analysis	0.821	0.919	0.733	0.804	0.806	0.852	0.851	0.778	0.761	0.861
GP Practice – sample taken by a healthcare professional	1.019	1.049	0.990	1.085	1.035	0.941	0.994	1.066	0.885	1.101
Sexual Health Clinic – sample taken by a healthcare professional	1.000	1.000	1.000	1.000	1.000	1.000	1.000	1.000	1.000	1.000
**2. Time to Result**										
30 Mins	1.806	1.828	1.786	1.763	1.888	1.772	1.730	1.896	1.808	1.797
2 Hours	1.402	1.365	1.442	1.326	1.548	1.344	1.311	1.516	1.357	1.423
7 Days	1.000	1.000	1.000	1.000	1.000	1.000	1.000	1.000	1.000	1.000
14 Days	0.862	0.844	0.881	0.878	0.882	0.828	0.862	0.862	0.882	0.856
**3. Accuracy**										
2 in 100 False Negative	3.242	2.951	3.570	3.307	3.161	3.282	3.275	3.305	3.000	3.482
5 in 100 False Negative	1.000	1.000	1.000	1.000	1.000	1.000	1.000	1.000	1.000	1.000
**4. Consultation Method**										
Online Consultation	1.212	1.187	1.239	1.187	1.200	1.253	1.156	1.285	1.174	1.228
Pharmacy Consultation	1.158	1.106	1.215	1.167	1.209	1.102	1.115	1.225	1.098	1.183
GP Consultation	1.183	1.172	1.194	1.279	1.142	1.133	1.162	1.189	1.112	1.215
Sexual Health Clinic Consultation	1.000	1.000	1.000	1.000	1.000	1.000	1.000	1.000	1.000	1.000
**5. Access to Healthcare Professional**										
Phone	0.949	0.937	0.962	1.001	0.902	0.946	0.942	0.926	0.947	0.953
Instant Messenger	1.027	0.994	1.062	1.108	0.977	1.001	1.025	0.991	1.045	1.025
Email	1.048	1.034	1.062	1.078	1.038	1.030	1.069	1.001	1.049	1.054
Face to Face	1.000	1.000	1.000	1.000	1.000	1.000	1.000	1.000	1.000	1.000
**6. How you get Antibiotics**										
Post to Home	1.011	0.996	1.028	0.977	1.010	1.048	0.957	1.055	1.011	1.020
Post to Collection Point	1.031	1.082	0.983	1.010	1.036	1.047	0.968	1.072	1.034	1.035
Collect from Pharmacy	1.075	1.064	1.087	1.039	1.106	1.080	1.070	1.056	1.086	1.078
Collect from Sexual Health Clinic	1.000	1.000	1.000	1.000	1.000	1.000	1.000	1.000	1.000	1.000

Table 4 – Trade-off between Accuracy and Time (Probability of Uptake)

	Full Dataset	Male	Female	16-18	19-21	22-24	Single	One Partner	Prev Test	No Prev Test
Pathways: (n in 100[1] / time[2])										
5 in 100[1]/ 30min[2]	38%	40%	35%	36%	39%	38%	36%	39%	40%	36%
2 in 100/ 14 days	58%	55%	62%	59%	58%	59%	59%	58%	58%	59%
Not Test	4%	5%	3%	5%	3%	3%	4%	3%	2%	5%
Total	100%	100%	100%	100%	100%	100%	100%	100%	100%	100%

[1] Test accuracy = n in 100 people will be told they don't have chlamydia when they do

[2] Time to receipt of result

For peer review only

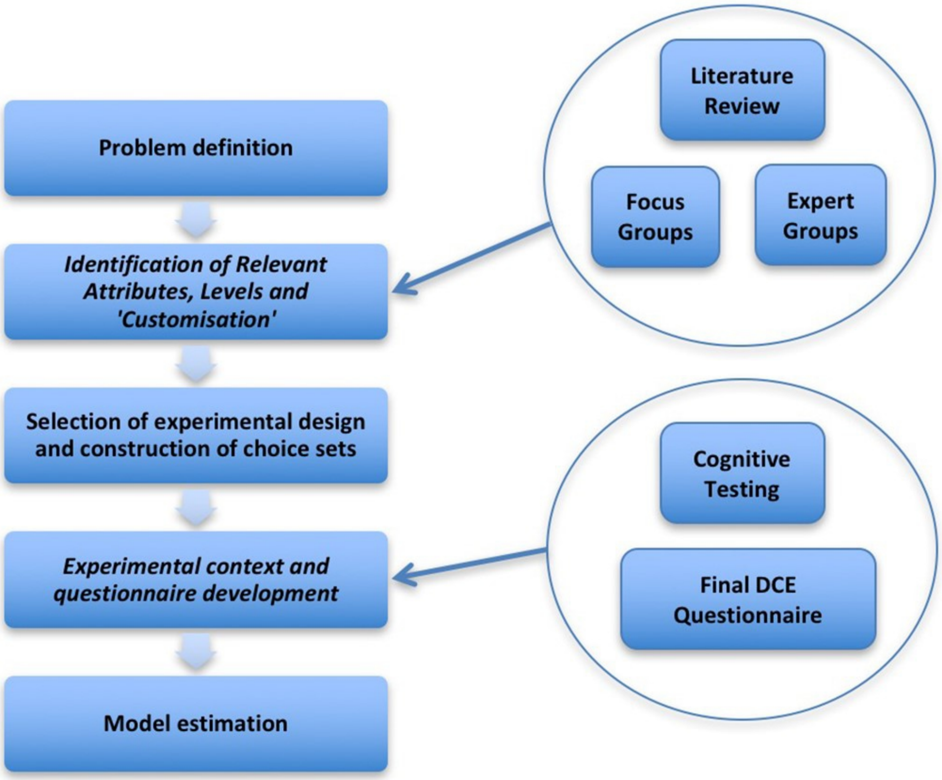

Figure 1 – Stages in Conducting a Discrete Choice Experiment

90x90mm (300 x 300 DPI)

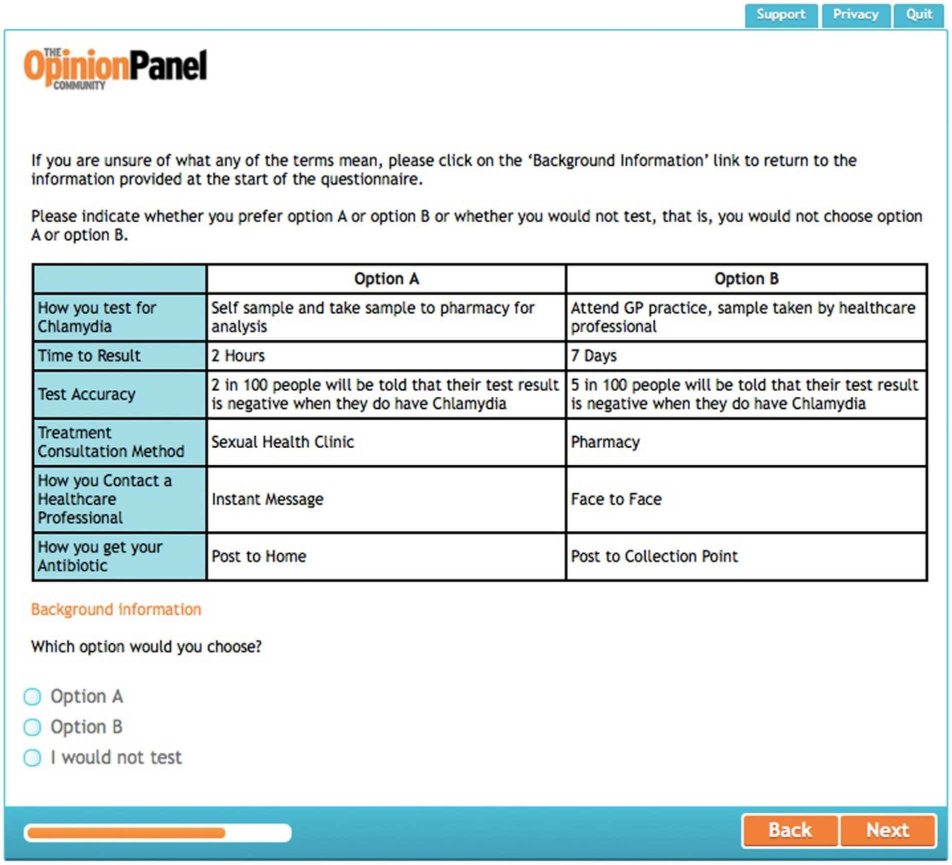

Support Privacy Quit

THE OpinionPanel COMMUNITY

If you are unsure of what any of the terms mean, please click on the ‘Background Information’ link to return to the information provided at the start of the questionnaire.

Please indicate whether you prefer option A or option B or whether you would not test, that is, you would not choose option A or option B.

	Option A	Option B
How you test for Chlamydia	Self sample and take sample to pharmacy for analysis	Attend GP practice, sample taken by healthcare professional
Time to Result	2 Hours	7 Days
Test Accuracy	2 in 100 people will be told that their test result is negative when they do have Chlamydia	5 in 100 people will be told that their test result is negative when they do have Chlamydia
Treatment Consultation Method	Sexual Health Clinic	Pharmacy
How you Contact a Healthcare Professional	Instant Message	Face to Face
How you get your Antibiotic	Post to Home	Post to Collection Point

Background information

Which option would you choose?

- Option A
- Option B
- I would not test

Back Next

Figure 2 – Sample Choice Set from the Discrete Choice Experiment

99x90mm (300 x 300 DPI)

Supplementary File 1 – Selection of Attributes and Levels

Four research stages were used to determine the attributes and associated levels included in the Discrete Choice Experiment.

1. LITERATURE REVIEWS

1.1. Overarching Objectives

- To identify a long list of attributes
- To identify potential levels
- To identify gaps in knowledge
- To inform the development of the focus groups

1.2. Literature Review 1 - Use of Stated Preference Studies for STI testing & treatment services

1.2.1. Objective

To identify and appraise published studies exploring which attributes influence patient and clinician preferences for the testing and treatment of STIs.

1.2.2. Inclusion/ Exclusion Criteria

The inclusion criteria were identified as:

- any stated preference study within the scope of STI testing and treatment services. This included but was not limited to products (e.g. tests, drugs, condoms, microbicides) and services (e.g. screening and screening programmes, and service providers e.g. GPs, CaSH clinics and GUM clinics);
- There was no date limiter, with all published studies included to end of 2014.

Exclusion criteria included studies:

- not related to humans;
- not published in English;
- from outside of the OECD High Income Countries;
- not related to the diagnosis or treatment of STIs (e.g. vaccinations).

1.2.3. Search Strategy

The following databases were searched on 28 April 2014 to identify studies published to the end of 2013, the saved database searches were re-run in April 2015 to search for any studies meeting the inclusion criteria published between 1 January and 31 December 2014 and no further studies were identified:

- Medline
- EMBASE
- CINAHL
- Web of Science
- Econlit

For peer review only - http://bmjopen.bmj.com/site/about/guidelines.xhtml

- PsycINFO
- Cochrane Library (incorporating the following databases):
  - Cochrane Database of Systematic Reviews
  - Cochrane Central Register of Controlled Trials
  - Cochrane Methodology Register
  - Database of Abstracts of Reviews of Effects
  - Health Technology Assessment Database
  - NHS Economic Evaluation Database

Searches were structured to meet the search requirements of the respective database and terms expanded where the facility existed to do this.

1.3. Literature Review 2 – Preferences for and acceptability of mainstream sexual health services

1.3.1. Objective

The objective of this literature review was to identify which factors might influence individuals' decisions to access testing and treatment services for STIs.

1.3.2. Inclusion/ Exclusion Criteria

The inclusion criteria were identified as:

- any study which indicates individuals' preferences or acceptability of STI testing and/ or treatment services;
- studies published between Jan 2004-Sept 2014;
- conference abstracts, where the abstract enabled the extraction of information on study focus and key findings.

The date range for the literature review was selected to limit the volume of results identified to the last 10 years. This recognised the fact that the previous literature review only identified one stated preference study which met the inclusion criteria pre-2004, and that none of the studies identified included relevant new technologies e.g. internet or smartphone based services.

The exclusion criteria were identified as any study:

- not published in English
- not related to humans
- not related to preferences for sexual health services
- from outside of the OECD High Income Country List
- not related directly to testing and/ or treatment provision e.g. drug characteristics, health promotion interventions
- not offering a perspective provided by service user/ potential service user e.g. clinician
- focused on non-mainstream service provision e.g. STI testing in A&E, dedicated service provision for specific high risk groups such as men recently released from prison, sex workers, injecting drug users.

For peer review only - http://bmjopen.bmj.com/site/about/guidelines.xhtml

1.3.3. Search Strategy

The key search terms included were:

- Sexually transmitted infections, sexually transmitted diseases, sexual health
- Test, treatment, service
- Patient preference, acceptability, choice, uptake, access.

Individual STIs (e.g. Chlamydia) were not included in the search strategy, as it was determined that attributes and levels could potentially be identified from any STI/ sexual health product or service.

A smaller selection of databases was chosen for this scoping review, with the databases selected being those which had generated a high return rate of relevant articles in the first literature review and encompassing journals where research relating to nursing, AHPs and psychology is published.
The three databases searched were:

- Medline
- CINAHL
- PsycINFO.

2. FOCUS GROUPS

2.1. Objectives

Primary Objective:

- To identify which the themes and factors young people consider important when choosing whether to test for sexually transmitted infections

Secondary Objectives:

- To gain insight into:
    - Reasons for importance of factors
    - Rationale for trading between factors and prioritisation
    - How participants articulate views and opinions on these themes/ factors.

2.2. Inclusion/ Exclusion Criteria

Participant inclusion criteria set for the focus groups were:

- Between the ages of 16 and 24
- Ability to speak English
- Able to consent.

Exclusion criteria were:

- People under the age of 16 and over the age of 25
- People requiring any form of interpreting/ translation services to participate
- People unable to give consent.

For peer review only - http://bmjopen.bmj.com/site/about/guidelines.xhtml

2.3. Methods

2.3.1. Sampling

Convenience sampling was ultimately chosen for this research due to the challenges with accessing the population and being able to undertake purposive sampling within the time constraints of this phase of the research.

2.3.2. Data Management & Analysis

Thematic analysis was selected as the core approach to analyse the focus group data recognising that this is one of the three 'main methods' for analysing focus group data and is the method most closely aligned to that used to design the focus group.

3. EXPERT GROUPS

3.1. Objectives

The objectives of the expert groups were to consider whether:

- The potential attributes met best practice requirements as defined by ISPOR Conjoint Analysis working group (Bridges et al., 2011) as "a subset of all possible attributes can be determined on the basis of three criteria:
  - Relevance to the research question;
  - Relevance to the decision context;
  - Whether attributes are related to one another (Bridges et al., 2011:406);
- Any key attributes omitted that may lead response bias;
- Levels are sufficiently reflective of current and potential future technology developments.

3.2. Methods

Expert groups were run as focus groups to enable discussion around a series of key questions. The key issues raised by each of the expert groups were summarised by attribute using a matrix. The expert groups were not recorded, fully transcribed or analysed thematically as their purpose was to build on the outputs of the focus groups and capture the salient points for consideration, which was achievable through the matrix coding.

4. NARRATIVE SYNTHESIS

4.1. Objectives

The purpose of the focus and expert groups was to inform the selection of attributes and levels for the DCE. Achieving a balance between attributes of importance to the study population and attributes which will deliver impact to services and technology developers was the key consideration in selection. This led to the decision that a formal ranking or consensus method should not be used with either the focus or expert groups as a combined view of both was required. Instead, the approach adopted was a narrative synthesis developed for implementation reviews to enable

For peer review only - http://bmjopen.bmj.com/site/about/guidelines.xhtml

the outputs from the focus groups, expert groups and literature review to be synthesised against each potential attribute (Pope et al., 2007).

4.2. Methods

The approach taken in the narrative synthesis is outlined below, adapted from the key elements of synthesis identified by Pope and colleagues (2007):

Element of Synthesis	Approach Taken
**Developing a Theoretical Model**	Identification of a checklist of properties against which attributes can be considered to inform the selection process
**Developing a Preliminary Synthesis**	Tabulation against the checklist – to create a matrix of checklist criteria against attributes to enable visual comparison of perspective
**Exploring Relationships in the Data**	Conceptual mapping and triangulation against the checklist
**Assessing the Robustness of the Synthesis Product**	Critical reflection on the synthesis process

For peer review only

Supplementary File 2 – DCE Questionnaire Introduction and Background Information

Developing Chlamydia Testing & Treatment Services

This survey is designed to find out more about your views on what is important to you in accessing services to get tested for chlamydia, and treatment if your test result is positive (i.e. you have chlamydia). This forms part of a research project being undertaken at the University of Warwick, please click here to access further information about the study and how your information will be used [insert url to participant information leaflet]. By continuing to complete the survey you are consenting for your responses to be used for research in accordance with the information set out in the study information leaflet. Your responses will help us understand what is important to young people to shape how services are developed in the future.

Please read through the following background information before completing the survey. It contains information about chlamydia and the terms that are used in the survey.

What is Chlamydia?

Chlamydia is the most common sexually transmitted infection in England and the majority of infections are found in young people aged 15-24. Both men and women can get chlamydia, but most people with chlamydia have no symptoms and do not know they have an infection. The test for chlamydia is usually a urine sample for men and a vaginal swab for women. Once diagnosed, chlamydia can be treated with a single dose (2 or 4 tablets) of an antibiotic called Azithromycin.

In England there is a national chlamydia screening programme which recommends that people aged 16-24 are tested for chlamydia annually or when they change their sexual partner.

What happens if I don’t get treatment?

If left undiagnosed chlamydia can lead to serious health problems.

In women this includes a condition called Pelvic Inflammatory Disease, which is an infection of the womb, fallopian tubes or ovaries. This can cause severe pain and in some cases (around 1 in 10) lead to infertility or ectopic pregnancy (where the pregnancy occurs outside of the womb in the fallopian tube). If you are pregnant and have chlamydia you can pass it on to your baby when you give birth and this can lead to the baby getting an eye infection or pneumonia.

For peer review only - http://bmjopen.bmj.com/site/about/guidelines.xhtml

In men chlamydia can lead to a condition called epididymitis, which causes soreness and swelling in the scrotum.

For further information

If you would like further information on chlamydia and how to prevent it, or any of the other conditions described please visit:
http://www.nhs.uk/conditions/chlamydia/Pages/Introduction.aspx

About the Survey…

In completing the survey you will be presented with a number of choices for getting tested and treatment for chlamydia. Please consider each set of choices and indicate whether you prefer option A or option B or whether you would not test, that is, you would not choose option A or option B.

Each choice involves two different scenarios for testing and getting treatment and there are differences between how you test, how long you have to wait for the result, how accurate the test is, how you have a consultation to get treatment for chlamydia, how you access a healthcare professional to get that treatment and how you get your antibiotic.

The following sections provide you with an explanation of the process of getting tested and treatment and the key terms used in the survey:

The diagram below shows the stages up until you find out our result:

For peer review only - http://bmjopen.bmj.com/site/about/guidelines.xhtml

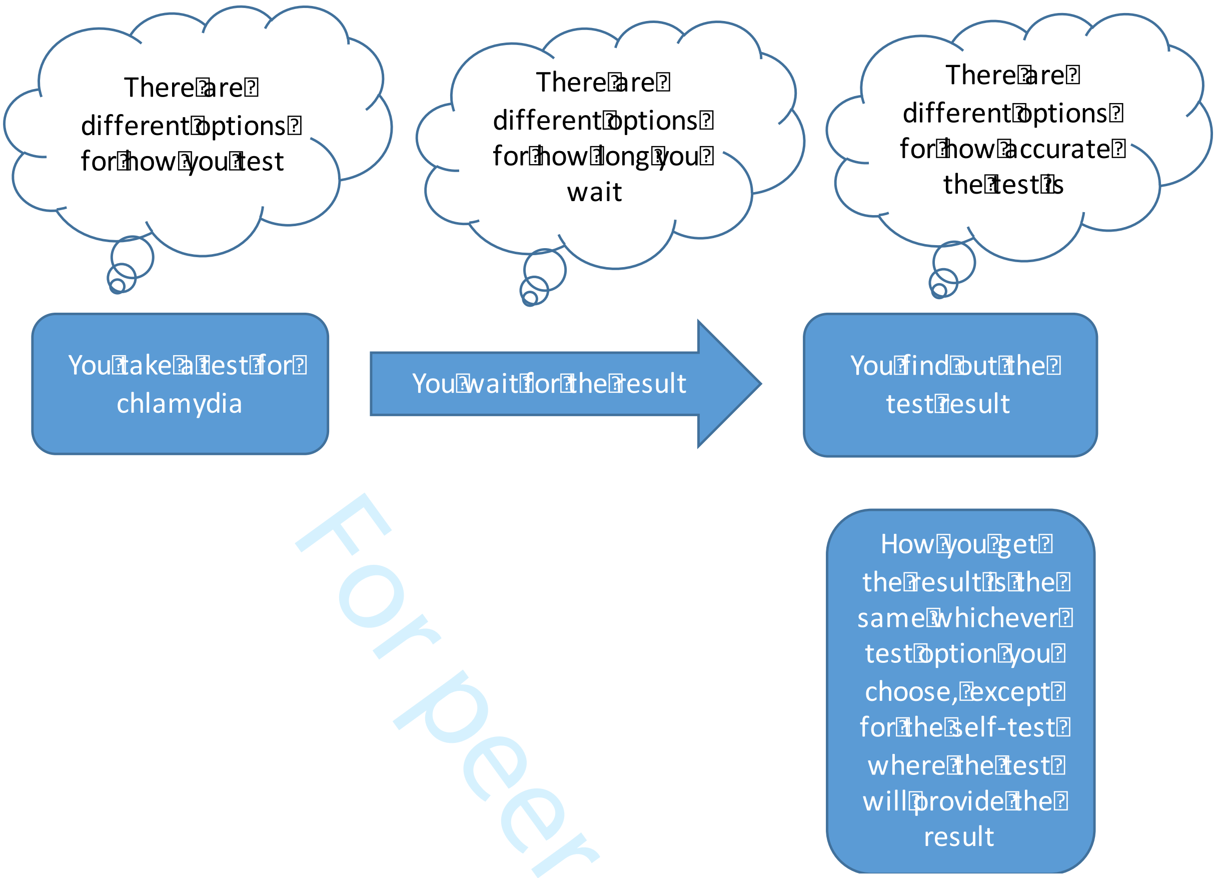

For peer review only

The following sections provide more information on how you test, how long you wait for the result and the accuracy of the test.

How you test for Chlamydia

This focuses on how you get your test, how the sample is taken and what happens to the sample once you've taken it. There are six options in the questionnaire, an explanation of these is provided below:

- **Self-Test -** Order a test kit online or collect one from a community location e.g. pharmacy or supermarket, provide the sample yourself and interpret the result yourself (like a pregnancy test). This is different to the self-sample options below because you interpret the result yourself rather than it being interpreted by a healthcare professional.
- **Self-Sample and post off for analysis -** Order a test kit online or collect one from a community location e.g. pharmacy or supermarket, provide the sample yourself and send the sample in the freepost envelope to the laboratory for analysis. This is different to the self-test option above because the test is interpreted by a healthcare professional.
- **Self-Sample and take to pharmacy for analysis -** Order a test kit online or collect one from a community location e.g. pharmacy or supermarket, provide the sample yourself and then take the sample to a pharmacy for analysis. This is different to the self-test option above because the test is interpreted by a

For peer review only - http://bmjopen.bmj.com/site/about/guidelines.xhtml

healthcare professional. Opening hours vary between pharmacies but many in towns and cities are open evenings and weekends as well as weekdays.

- **Self-Sample and take to your place of education/ workplace for analysis -** Order a test kit online or collect one from a community location e.g. pharmacy or supermarket, provide the sample yourself and take the sample to your place of education/ workplace for analysis by an outreach nurse. This is different to the self-test option above because the test is interpreted by a healthcare professional. Outreach services in education and workplaces are usually available one day a week.
- **Attend GP Practice, sample taken by a GP or Nurse -** Book an appointment at your GP practice with a GP or practice nurse. Your sample will be taken by the healthcare professional you see. GP practices are routinely open Monday to Friday 8am – 6.30pm.
- **Attend sexual health clinic, sample taken by a Doctor or Nurse -** Drop in or book an appointment with a doctor or nurse at a sexual health clinic. Your sample will be taken by the health care professional you see. Sexual Health Clinics are located in towns and cities, opening hours vary but clinics are generally open during the working day Monday to Friday, some evenings during the week and Saturdays.

Taking a sample yourself is straightforward and does not affect the accuracy of the test result.

Time to Result

How long it takes to get the test result. For the self-test option, this is how long it takes from you doing the test to you being able to read the result. For the other options this is how long it takes from you posting or dropping off your sample to when you are given your result. There are four options:

- 30 Mins
- 2 Hours
- 7 Days
- 14 Days

The time it takes to get the result does not affect the accuracy of the test.

Test Accuracy

This tells you how accurate your test result is. In this survey we want you to consider the likelihood of the test telling you that you don't have chlamydia when in fact you do. There are two options:

1. 2 in 100 people will be told that their test result is negative when they do have chlamydia
2. 5 in 100 people will be told their test result is negative when they do have chlamydia

For peer review only - http://bmjopen.bmj.com/site/about/guidelines.xhtml

Another way of describing this is shown below:

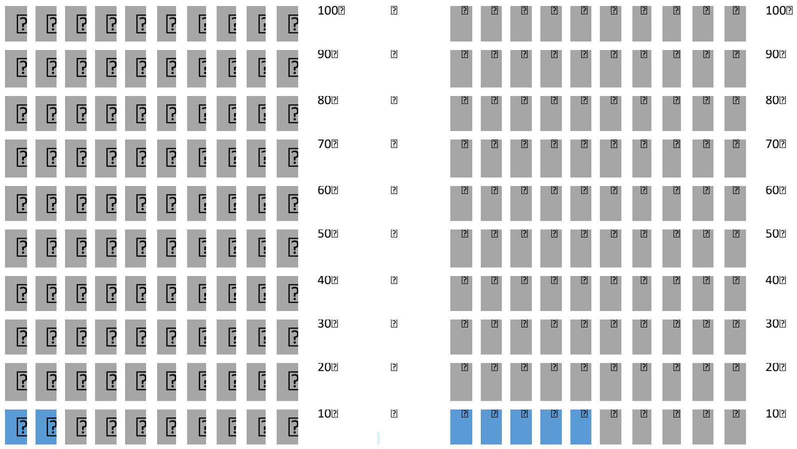

Each graph represents 100 people

= People who are told their test result is negative when they do have chlamydia

We would also like you to think about how you would choose to get treatment if your test result is positive. The diagram below shows the stages to get treatment:

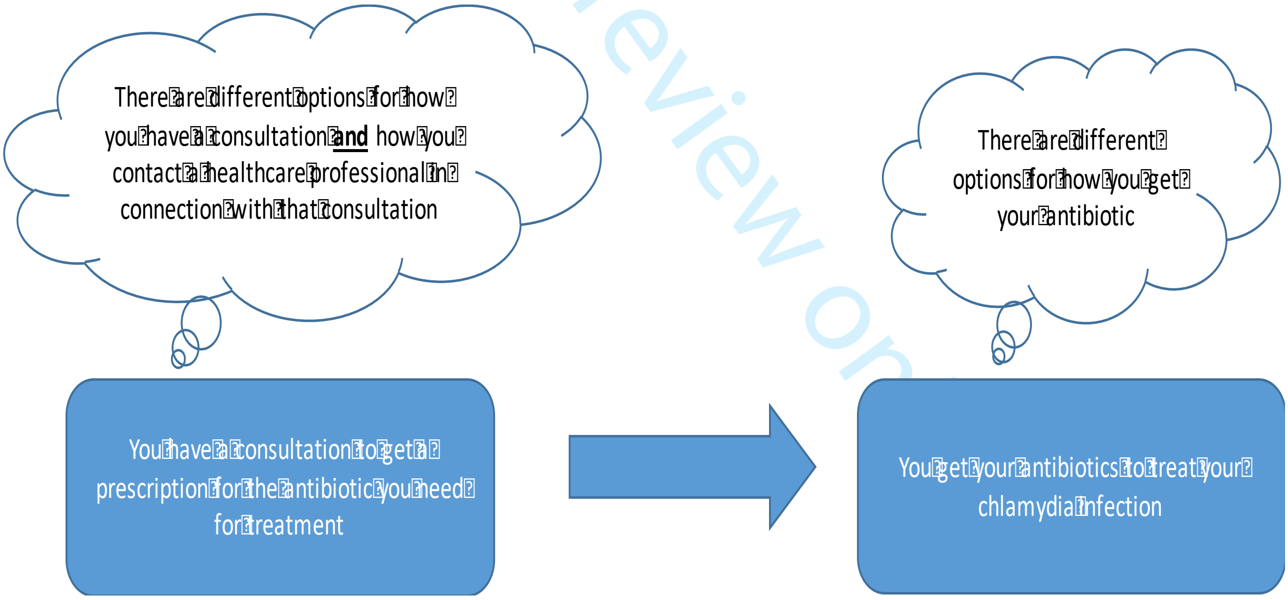

The following sections provide more information on how you get your treatment, how you contact a healthcare professional and how you get your antibiotics.

How you get your treatment

If your test result is positive there are a number of different options for you to get your treatment for chlamydia. There are four options in this survey:

For peer review only - http://bmjopen.bmj.com/site/about/guidelines.xhtml

- Complete an online consultation – this is available 24 hours a day, 7 days a week. You can access a healthcare professional if you need any help or advice between 9am and 5pm Monday to Friday.
- Have a consultation with a pharmacist - Opening hours vary between pharmacies but many in towns and cities are open evenings and weekends as well as weekdays.
- Have a consultation with a GP - GP practices are routinely open Monday to Friday 8am – 6.30pm.
- Have a consultation with a doctor or nurse at a Sexual Health Clinic - Sexual Health Clinics are located in towns and cities, opening hours vary but clinics are generally open during the working day Monday to Friday, some evenings during the week and Saturdays.

How you Contact a Healthcare Professional

New technology means that there are now more options for completing your consultation to get treatment, or in the case of the online consultation, accessing a healthcare professional for advice. There are four options within the questionnaire:

- Telephone – speak to a healthcare professional on the telephone.
- Instant Messaging – have a discussion with a healthcare professional via an instant messaging service online
- Email – have a discussion with a healthcare professional via email. During opening hours you will receive a response within 2 hours, and for emails sent out of hours you will receive a response the next day.
- Face-to-face - have a face-to-face discussion with a healthcare professional.

How you get your antibiotics

Once you've completed your consultation you need to get your antibiotic tablets. In this survey there are four options available to you:

- Deliver to home address – provide your home address for your antibiotic to be posted to you. Your antibiotic will arrive within 1-2 working days.
- Deliver to collection point – nominate an address other than your home address, for example a friend's address or a collection point for your antibiotic to be posted to. Your antibiotic will arrive within 1-2 working days.
- Collect from Pharmacy – attend a pharmacy of your choice with your prescription and the pharmacist gives you the antibiotic tablets
- Collect from Sexual Health Clinic – attend a sexual health clinic and the doctor or nurse gives you the antibiotic tablets

In all of the choices available to you there are a number of things that you should assume are the same:

- The service is provided to you free of charge although there may be a cost to you, for example in making phone calls, accessing the internet, or taking time off work to go to an appointment.

For peer review only - http://bmjopen.bmj.com/site/about/guidelines.xhtml

- The healthcare professional who is providing your treatment is trained to be able to provide the service.
- How you get your results is the same whichever option you choose, except for the self-test where the test will provide the result.
- Your personal data is managed securely in whichever option you choose

More choices for an option does not mean that it is more or less important, it just reflects that there are more choices within the scenarios you are being asked to consider.

For peer review only

For peer review only - http://bmjopen.bmj.com/site/about/guidelines.xhtml

Supplementary File 3 – DCE Results

Part 1 - Demographic Characteristics for Sub-Group Analysis

Demographic Characteristic	n	%	National %[1]
**Age**			
16	8	1%	10%
17	113	9%	10%
18	294	24%	11%
19	183	15%	11%
20	132	11%	11%
21	91	7%	11%
22	162	13%	11%
23	135	11%	12%
24	112	9%	12%
Total	1230	100%	99.00%
**Gender**			
Male	607	49%	51%
Female	623	51%	49%
Total	1230	100%	100%
**Ethnicity**			
White – English, Welsh, Scottish, Northern Irish, British	932	76%	80%
White – Irish	7	1%	1%
White – Gypsy or Irish Traveller	1	0%	0%
White – Any other white background	37	3%	5%
Mixed/ Multiple Ethnic Groups – White & Black Caribbean	10	1%	1%
Mixed/ Multiple Ethnic Groups – White & Black African	6	0%	0%
Mixed/ Multiple Ethnic Groups – White & Asian	13	1%	1%
Mixed/ Multiple Ethnic Groups – Any other mixed/ multiple ethnic background	8	1%	1%
Asian/ Asian British – Indian	50	4%	3%
Asian/ Asian British - Pakistani	38	3%	2%
Asian/ Asian British - Bangladeshi	18	1%	1%
Asian/ Asian British - Chinese	22	2%	1%
Asian/ Asian British – Any other Asian background	21	2%	2%
Black/ African/ Caribbean/ Black British – African	26	2%	2%

[1] - National Data for Age and Region taken from ONS Mid-Year Population Estimates 2015 (ONS, 2016b). Percentages derived from the total 16-24 population. National Data for Ethnicity taken from Census 2011 data, percentages derived from total England population (ONS, 2012). Percentages may not sum due to rounding

Demographic Characteristic	n	%	National %[1]
Black/ African/ Caribbean/ Black British – Caribbean	10	1%	1%
Black/ African/ Caribbean/ Black British – Any other black/ African/ Caribbean background	2	0%	1%
Other – Arab	5	0%	0%
Other – Any other ethnic group	9	1%	1%
I would prefer not to say	15	1%	0%
Total	1230	100%	103.00%
**Region**			
East Midlands	117	10%	9%
London	225	18%	15%
North East	74	6%	5%
North West	123	10%	13%
Eastern	55	4%	10%
South East	222	18%	16%
South West	143	12%	10%
West Midlands	144	12%	11%
Yorkshire and The Humber	113	9%	11%
I would prefer not to say	14	1%	0%
Total	1230	100%	100.00%
**Sexual Preference**			
Heterosexual (partner of opposite sex)	1030	84%	
Homosexual (partner of same sex) & Male	13	1%	
Homosexual (partner of same sex) & Female	39	3%	
Bisexual (partner of either sex)	98	8%	
I would prefer not to say	50	4%	
Total	1230	100%	
**Previous STI Test**			
Yes	393	32%	
No	790	64%	
I would prefer not to say	47	4%	
Total	1230	100%	
**Relationship Status**			
Single	615	50%	
In a non-sexual relationship with one person	36	3%	
In a non-sexual relationship with more than one person	2	0%	
In a sexual relationship with one person	512	42%	
In a sexual relationship with more than one person	36	3%	
I would prefer not to say	29	2%	
Total	1230	100%	

Part 2 - Validity Checks

Table 2.1 – Comparison of Full Dataset Coefficients and Odds Ratios for the Full Dataset for Dataset including Choice Set 1 and Dataset including Choice Set 25. Data highlighted in blue indicate results which are not statistically significant.

	**Dataset Including Choice Set 1, *n=1,230***						**Dataset Including Choice Set 25, *n=1,230***					
	**Coefficient**	**Lower 95% CI**	**Upper 95% CI**	**Odds Ratio**	**Lower 95% CI**	**Upper 95% CI**	**Coefficient**	**Lower 95% CI**	**Upper 95% CI**	**Odds Ratio**	**Lower 95% CI**	**Upper 95% CI**
**How you Test**												
Self-Test	0.498	0.432	0.564	1.646	1.540	1.758	0.481	0.415	0.547	1.618	1.514	1.729
Post	0.308	0.239	0.376	1.360	1.270	1.456	0.306	0.239	0.373	1.358	1.271	1.452
Pharmacy	0.116	0.056	0.175	1.123	1.057	1.192	0.144	0.085	0.203	1.155	1.088	1.226
Education/ Work	-0.206	-0.266	-0.146	0.814	0.766	0.864	-0.197	-0.257	-0.137	0.821	0.773	0.872
GP Practice	0.011	-0.050	0.073	1.011	0.951	1.075	0.019	-0.042	0.081	1.019	0.959	1.084
Sexual Health Clinic	0.000	-	-	1.000	-	-	0.000	-	-	1.000	-	-
**Time to Result**												
30 Mins	0.579	0.524	0.633	1.784	1.688	1.884	0.591	0.537	0.645	1.806	1.711	1.906
2 Hours	0.340	0.283	0.398	1.406	1.328	1.488	0.338	0.281	0.395	1.402	1.324	1.485
7 Days	0.000	-	-	1.000	-	-	0.000	-	-	1.000	-	-
14 Days	-0.149	-0.200	-0.099	0.861	0.819	0.906	-0.148	-0.198	-0.098	0.862	0.820	0.907
**Accuracy**												
2 in 100 False Negative	1.192	1.157	1.226	3.292	3.179	3.409	1.176	1.141	1.212	3.242	3.130	3.359
5 in 100 False Negative	0.000	-	-	1.000	-	-	0.000	-	-	1.000	-	-

For peer review only

	Dataset Including Choice Set 1, *n=1,230*						Dataset Including Choice Set 25, *n=1,230*					
	Coefficient	Lower 95% CI	Upper 95% CI	Odds Ratio	Lower 95% CI	Upper 95% CI	Coefficient	Lower 95% CI	Upper 95% CI	Odds Ratio	Lower 95% CI	Upper 95% CI
**Consultation Method**												
Online Consultation	0.185	0.133	0.236	1.203	1.142	1.266	0.192	0.140	0.245	1.212	1.150	1.277
Pharmacy Consultation	0.150	0.099	0.202	1.162	1.104	1.224	0.147	0.095	0.199	1.158	1.100	1.220
GP Consultation	0.146	0.095	0.197	1.157	1.100	1.218	0.168	0.116	0.220	1.183	1.123	1.246
Sexual Health Clinic Consultation	0.000	-	-	1.000	-	-	0.000	-	-	1.000	-	-
**Access to Healthcare Professional**												
Phone	-0.058	-0.108	-0.008	0.943	0.897	0.992	-0.052	-0.102	-0.002	0.949	0.903	0.998
Instant Messenger	0.020	-0.029	0.070	1.020	0.971	1.072	0.027	-0.023	0.076	1.027	0.977	1.079
Email	0.027	-0.023	0.076	1.027	0.978	1.079	0.047	-0.002	0.096	1.048	0.998	1.101
Face to Face	0.000	-	-	1.000	-	-	0.000	-	-	1.000	-	-
**How you get Antibiotics**												
Post to Home	0.038	-0.013	0.089	1.039	0.987	1.093	0.011	-0.041	0.063	1.011	0.960	1.065
Post to Collection Point	0.052	0.002	0.101	1.053	1.002	1.106	0.030	-0.020	0.081	1.031	0.980	1.085
Collect from Pharmacy	0.091	0.038	0.143	1.095	1.039	1.154	0.072	0.018	0.126	1.075	1.018	1.134
Collect from Sexual Health Clinic	0.000	-	-	1.000	-	-	0.000	-	-	1.000	-	-
**Choice**												
Option A	0.000	-	-	1.000	-	-	0.000	-	-	1.000	-	-
Option B	-0.341	-0.381	-0.302	0.711	0.683	0.740	-0.360	-0.400	-0.320	0.698	0.670	0.726
I would not test	-1.697	-1.797	-1.596	0.183	0.166	0.203	-1.682	-1.781	-1.583	0.186	0.168	0.205

For peer review only

Table 2.2 – Coefficients and Odds Ratios for the Full Dataset and the Dataset Excluding Different Responses to Choice Sets 1 and 25. Data highlighted in blue indicate results which are not statistically significant.

	**Full Dataset *n=1,230***						**Excl Incorrect Answer to Validity Check *n=912***					
	**Coefficient**	**Lower 95% CI**	**Upper 95% CI**	**Odds Ratio**	**Lower 95% CI**	**Upper 95% CI**	**Coefficient**	**Lower 95% CI**	**Upper 95% CI**	**Odds Ratio**	**Lower 95% CI**	**Upper 95% CI**
**How you Test**												
Self-Test	0.481	0.415	0.547	1.618	1.514	1.729	0.507	0.429	0.584	1.660	1.535	1.794
Post	0.306	0.239	0.373	1.358	1.271	1.452	0.333	0.254	0.411	1.395	1.290	1.508
Pharmacy	0.144	0.085	0.203	1.155	1.088	1.226	0.124	0.054	0.194	1.132	1.056	1.214
Education/ Work	-0.197	-0.257	-0.137	0.821	0.773	0.872	-0.253	-0.323	-0.183	0.777	0.724	0.833
GP Practice	0.019	-0.042	0.081	1.019	0.959	1.084	0.019	-0.053	0.091	1.019	0.948	1.095
Sexual Health Clinic	0.000	-	-	1.000	-	-	0.000	-	-	1.000	-	-
**Time to Result**												
30 Mins	0.591	0.537	0.645	1.806	1.711	1.906	0.573	0.510	0.637	1.774	1.665	1.890
2 Hours	0.338	0.281	0.395	1.402	1.324	1.485	0.335	0.268	0.402	1.397	1.307	1.494
7 Days	0.000	-	-	1.000	-	-	0.000	-	-	1.000	-	-
14 Days	-0.148	-0.198	-0.098	0.862	0.820	0.907	-0.148	-0.177	-0.236	-0.117	0.790	0.889
**Accuracy**												
2 in 100 False Negative	1.176	1.141	1.212	3.242	3.130	3.359	1.323	1.282	1.364	3.755	3.602	3.913
5 in 100 False Negative	0.000	-	-	1.000	-	-	0.000	-	-	1.000	-	-
**Consultation Method**												
Online Consultation	0.192	0.140	0.245	1.212	1.150	1.277	0.197	0.136	0.258	1.218	1.145	1.294
Pharmacy Consultation	0.147	0.095	0.199	1.158	1.100	1.220	0.173	0.112	0.234	1.189	1.119	1.264
GP Consultation	0.168	0.116	0.220	1.183	1.123	1.246	0.179	0.119	0.240	1.196	1.126	1.271
Sexual Health Clinic Consultation	0.000	-	-	1.000	-	-	0.000	-	-	1.000	-	-

For peer review only

	Full Dataset *n=1,230*						Excl Incorrect Answer to Validity Check *n=912*					
	**Coefficient**	**Lower 95% CI**	**Upper 95% CI**	**Odds Ratio**	**Lower 95% CI**	**Upper 95% CI**	**Coefficient**	**Lower 95% CI**	**Upper 95% CI**	**Odds Ratio**	**Lower 95% CI**	**Upper 95% CI**
**Access to Healthcare Professional**												
Phone	-0.052	-0.102	-0.002	0.949	0.903	0.998	-0.084	-0.143	-0.026	0.919	0.867	0.975
Instant Messenger	0.027	-0.023	0.076	1.027	0.977	1.079	-0.026	-0.084	0.031	0.974	0.919	1.032
Email	0.047	-0.002	0.096	1.048	0.998	1.101	-0.005	-0.063	0.052	0.995	0.939	1.054
Face to Face	0.000	-	-	1.000	-	-	0.000	-	-	1.000	-	-
**How you get Antibiotics**												
Post to Home	0.011	-0.041	0.063	1.011	0.960	1.065	0.014	-0.047	0.075	1.014	0.954	1.078
Post to Collection Point	0.030	-0.020	0.081	1.031	0.980	1.085	0.023	-0.036	0.083	1.024	0.964	1.086
Collect from Pharmacy	0.072	0.018	0.126	1.075	1.018	1.134	0.065	0.001	0.128	1.067	1.001	1.137
Collect from Sexual Health Clinic	0.000	-	-	1.000	-	-	0.000	-	-	1.000	-	-
**Choice**												
Option A	0.000	-	-	1.000	-	-	0.000	-	-	1.000	-	-
Option B	-0.360	-0.400	-0.320	0.698	0.670	0.726	-0.327	-0.374	-0.279	0.721	0.688	0.756
I would not test	-1.682	-1.781	-1.583	0.186	0.168	0.205	-1.674	-1.790	-1.557	0.188	0.167	0.211

For peer review only

Table 2.3 – Coefficient and Odds Ratios for the Full Dataset and the Dataset Containing Five Minutes or Longer to Complete. Data highlighted in blue indicate results which are not statistically significant.

	Full Dataset *n=1,230*						Data Excluding Responses taking less than 5 min to complete *n= 997*					
	Coefficient	Lower 95% CI	Upper 95% CI	Odds Ratio	Lower 95% CI	Upper 95% CI	Coefficient	Lower 95% CI	Upper 95% CI	Odds Ratio	Lower 95% CI	Upper 95% CI
**How you Test**												
Self-Test	0.481	0.415	0.547	1.618	1.514	1.729	0.542	0.467	0.616	1.719	1.595	1.852
Post	0.306	0.239	0.373	1.358	1.271	1.452	0.344	0.269	0.419	1.411	1.309	1.521
Pharmacy	0.144	0.085	0.203	1.155	1.088	1.226	0.168	0.101	0.234	1.183	1.106	1.264
Education/ Work	-0.197	-0.257	-0.137	0.821	0.773	0.872	-0.245	-0.312	-0.177	0.783	0.732	0.837
GP Practice	0.019	-0.042	0.081	1.019	0.959	1.084	-0.008	-0.077	0.061	0.992	0.925	1.062
Sexual Health Clinic	0.000	-	-	1.000	-	-	0.000	-	-	1.000	-	-
**Time to Result**												
30 Mins	0.591	0.537	0.645	1.806	1.711	1.906	0.642	0.581	0.703	1.900	1.788	2.019
2 Hours	0.338	0.281	0.395	1.402	1.324	1.485	0.372	0.308	0.436	1.451	1.361	1.547
7 Days	0.000	-	-	1.000	-	-	0.000	-	-	1.000	-	-
14 Days	-0.148	-0.198	-0.098	0.862	0.820	0.907	-0.147	-0.204	-0.091	0.863	0.816	0.913
**Accuracy**												
2 in 100 False Negative	1.176	1.141	1.212	3.242	3.130	3.359	1.308	1.269	1.348	3.700	3.556	3.850
5 in 100 False Negative	0.000	-	-	1.000	-	-	0.000	-	-	1.000	-	-
**Consultation Method**												
Online Consultation	0.192	0.140	0.245	1.212	1.150	1.277	0.217	0.159	0.276	1.243	1.172	1.318
Pharmacy Consultation	0.147	0.095	0.199	1.158	1.100	1.220	0.160	0.102	0.219	1.174	1.108	1.245
GP Consultation	0.168	0.116	0.220	1.183	1.123	1.246	0.171	0.113	0.229	1.187	1.120	1.258
Sexual Health Clinic Consultation	0.000	-	-	1.000	-	-	0.000	-	-	1.000	-	-
**Access to Healthcare Professional**												
Phone	-0.052	-0.102	-0.002	0.949	0.903	0.998	-0.042	-0.098	0.014	0.959	0.906	1.014
Instant Messenger	0.027	-0.023	0.076	1.027	0.977	1.079	0.043	-0.013	0.098	1.044	0.987	1.103
Email	0.047	-0.002	0.096	1.048	0.998	1.101	0.042	-0.013	0.097	1.043	0.987	1.102
Face to Face	0.000	-	-	1.000	-	-	0.000	-	-	1.000	-	-

For peer review only

	Full Dataset *n=1,230*						Data Excluding Responses taking less than 5 min to complete *n= 997*					
	**Coefficient**	**Lower 95% CI**	**Upper 95% CI**	**Odds Ratio**	**Lower 95% CI**	**Upper 95% CI**	**Coefficient**	**Lower 95% CI**	**Upper 95% CI**	**Odds Ratio**	**Lower 95% CI**	**Upper 95% CI**
**How you get Antibiotics**												
Post to Home	0.011	-0.041	0.063	1.011	0.960	1.065	0.001	-0.057	0.060	1.001	0.944	1.062
Post to Collection Point	0.030	-0.020	0.081	1.031	0.980	1.085	0.013	-0.044	0.070	1.013	0.957	1.072
Collect from Pharmacy	0.072	0.018	0.126	1.075	1.018	1.134	0.085	0.024	0.145	1.089	1.025	1.157
Collect from Sexual Health Clinic	0.000	-	-	1.000	-	-	0.000	-	-	1.000	-	-
**Choice**												
Option A	0.000	-	-	1.000	-	-	0.000	-	-	1.000	-	-
Option B	-0.360	-0.400	-0.320	0.698	0.670	0.726	-0.368	-0.413	-0.323	0.692	0.662	0.724
I would not test	-1.682	-1.781	-1.583	0.186	0.168	0.205	-1.776	-1.889	-1.662	0.169	0.151	0.190

For peer review only

Table 2.4 – Comparison of Full Dataset and Dataset Excluding Opt Out Responses. Data highlighted in blue indicate results which are not statistically significant.

	**Full Dataset** ***n=29,520 Choice Responses***						**Removal of 'I would not test' Data** ***n=27,727 Choice Responses***					
	**Coefficient**	**Lower 95% CI**	**Upper 95% CI**	**Odds Ratio**	**Lower 95% CI**	**Upper 95% CI**	**Coefficient**	**Lower 95% CI**	**Upper 95% CI**	**Odds Ratio**	**Lower 95% CI**	**Upper 95% CI**
**How you Test**												
Self-Test	0.481	0.415	0.547	1.618	1.514	1.729	0.501	0.433	0.568	1.650	1.542	1.765
Post	0.306	0.239	0.373	1.358	1.271	1.452	0.320	0.252	0.387	1.377	1.287	1.473
Pharmacy	0.144	0.085	0.203	1.155	1.088	1.226	0.152	0.092	0.212	1.164	1.096	1.236
Education/ Work	-0.197	-0.257	-0.137	0.821	0.773	0.872	-0.202	-0.262	-0.141	0.817	0.769	0.868
GP Practice	0.019	-0.042	0.081	1.019	0.959	1.084	0.027	-0.035	0.089	1.027	0.965	1.093
Sexual Health Clinic	0.000	-	-	1.000	-	-	0.000	-	-	1.000	-	-
**Time to Result**												
30 Mins	0.591	0.537	0.645	1.806	1.711	1.906	0.612	0.558	0.667	1.845	1.746	1.949
2 Hours	0.338	0.281	0.395	1.402	1.324	1.485	0.350	0.292	0.408	1.419	1.339	1.503
7 Days	0.000	-	-	1.000	-	-	0.000	-	-	1.000	-	-
14 Days	-0.148	-0.198	-0.098	0.862	0.820	0.907	-0.145	-0.196	-0.094	0.865	0.822	0.910
**Accuracy**												
2 in 100 False Negative	1.176	1.141	1.212	3.242	3.130	3.359	1.205	1.170	1.241	3.338	3.221	3.460
5 in 100 False Negative	0.000	-	-	1.000	-	-	0.000	-	-	1.000	-	-
**Consultation Method**												
Online Consultation	0.192	0.140	0.245	1.212	1.150	1.277	0.200	0.147	0.253	1.222	1.159	1.288
Pharmacy Consultation	0.147	0.095	0.199	1.158	1.100	1.220	0.159	0.106	0.211	1.172	1.112	1.235
GP Consultation	0.168	0.116	0.220	1.183	1.123	1.246	0.174	0.122	0.227	1.190	1.130	1.254
Sexual Health Clinic Consultation	0.000	-	-	1.000	-	-	0.000	-	-	1.000	-	-
**Access to Healthcare Professional**												
Phone	-0.052	-0.102	-0.002	0.949	0.903	0.998	-0.046	-0.097	0.004	0.955	0.908	1.004
Instant Messenger	0.027	-0.023	0.076	1.027	0.977	1.079	0.029	-0.021	0.079	1.029	0.979	1.082
Email	0.047	-0.002	0.096	1.048	0.998	1.101	0.050	0.000	0.100	1.051	1.000	1.105
Face to Face	0.000	-	-	1.000	-	-	0.000	-	-	1.000	-	-

For peer review only

	Full Dataset *n=29,520 Choice Responses*						Removal of 'I would not test' Data *n=27,727 Choice Responses*					
	**Coefficient**	**Lower 95% CI**	**Upper 95% CI**	**Odds Ratio**	**Lower 95% CI**	**Upper 95% CI**	**Coefficient**	**Lower 95% CI**	**Upper 95% CI**	**Odds Ratio**	**Lower 95% CI**	**Upper 95% CI**
**How you get Antibiotics**												
Post to Home	0.011	-0.041	0.063	1.011	0.960	1.065	0.012	-0.041	0.065	1.012	0.960	1.067
Post to Collection Point	0.030	-0.020	0.081	1.031	0.980	1.085	0.035	-0.016	0.086	1.036	0.984	1.090
Collect from Pharmacy	0.072	0.018	0.126	1.075	1.018	1.134	0.076	0.021	0.131	1.079	1.022	1.139
Collect from Sexual Health Clinic	0.000	-	-	1.000	-	-	0.000	-	-	1.000	-	-
**Choice**												
Option A	0.000	-	-	1.000	-	-	0.000	-	-	1.000	-	-
Option B	-0.360	-0.400	-0.320	0.698	0.670	0.726	-0.368	-0.409	-0.327	0.692	0.664	0.721
I would not test	-1.682	-1.781	-1.583	0.186	0.168	0.205	-	-	-	1.000		

For peer review only

Part 3 – DCE Results

(Cells highlighted in blue indicate result which is not statistically significant)

Table 3.1- Full Dataset Coefficients, Odds Ratios and Respective 95% Confidence Interval

	Full Dataset *n=1,230*					
	Coefficient	Lower 95% CI	Upper 95% CI	Odds Ratio	Lower 95% CI	Upper 95% CI
**How you Test**						
Self-Test	0.481	0.415	0.547	1.618	1.514	1.729
Post	0.306	0.239	0.373	1.358	1.271	1.452
Pharmacy	0.144	0.085	0.203	1.155	1.088	1.226
Education/ Work	-0.197	-0.257	-0.137	0.821	0.773	0.872
GP Practice	0.019	-0.042	0.081	1.019	0.959	1.084
Sexual Health Clinic	0.000	-	-	1.000	-	-
**Time to Result**						
30 Mins	0.591	0.537	0.645	1.806	1.711	1.906
2 Hours	0.338	0.281	0.395	1.402	1.324	1.485
7 Days	0.000	-	-	1.000	-	-
14 Days	-0.148	-0.198	-0.098	0.862	0.820	0.907
**Accuracy**						
2 in 100 False Negative	1.176	1.141	1.212	3.242	3.130	3.359
5 in 100 False Negative	0.000	-	-	1.000	-	-
**Consultation Method**						
Online Consultation	0.192	0.140	0.245	1.212	1.150	1.277
Pharmacy Consultation	0.147	0.095	0.199	1.158	1.100	1.220
GP Consultation	0.168	0.116	0.220	1.183	1.123	1.246
Sexual Health Clinic Consultation	0.000	-	-	1.000	-	-
**Access to Healthcare Professional**						
Phone	-0.052	-0.102	-0.002	0.949	0.903	0.998
Instant Messenger	0.027	-0.023	0.076	1.027	0.977	1.079
Email	0.047	-0.002	0.096	1.048	0.998	1.101
Face to Face	0.000	-	-	1.000	-	-
**How you get Antibiotics**						
Post to Home	0.011	-0.041	0.063	1.011	0.960	1.065
Post to Collection Point	0.030	-0.020	0.081	1.031	0.980	1.085
Collect from Pharmacy	0.072	0.018	0.126	1.075	1.018	1.134
Collect from Sexual Health Clinic	0.000	-	-	1.000	-	-

For peer review only

Table 3.2 - Gender Subgroup Analysis Coefficients and Odds Ratios with Associated 95% Confidence Intervals

	**Males *n=607***						**Females *n=623***					
	**Coefficient**	**Lower 95% CI**	**Upper 95% CI**	**Odds Ratio**	**Lower 95% CI**	**Upper 95% CI**	**Coefficient**	**Lower 95% CI**	**Upper 95% CI**	**Odds Ratio**	**Lower 95% CI**	**Upper 95% CI**
**How you Test**												
Self-Test	0.438	0.344	0.531	1.549	1.410	1.701	0.526	0.432	0.620	1.693	1.541	1.860
Post	0.269	0.174	0.363	1.308	1.190	1.438	0.344	0.250	0.439	1.411	1.284	1.551
Pharmacy	0.123	0.039	0.208	1.131	1.040	1.231	0.165	0.081	0.249	1.180	1.085	1.283
Education/ Work	-0.084	-0.169	0.001	0.919	0.845	1.001	-0.310	-0.395	-0.225	0.733	0.674	0.798
GP Practice	0.048	-0.039	0.135	1.049	0.962	1.145	-0.010	-0.097	0.077	0.990	0.908	1.080
Sexual Health Clinic	0.000	-	-	1.000	-	-	0.000	-	-	1.000	-	-
**Time to Result**												
30 Mins	0.603	0.527	0.680	1.828	1.694	1.973	0.580	0.503	0.656	1.786	1.654	1.928
2 Hours	0.311	0.230	0.392	1.365	1.259	1.479	0.366	0.285	0.447	1.442	1.329	1.563
7 Days	0.000	-	-	1.000	-	-	0.000	-	-	1.000	-	-
14 Days	-0.170	-0.241	-0.098	0.844	0.786	0.906	-0.127	-0.198	-0.056	0.881	0.820	0.945
**Accuracy**												
2 in 100 False Negative	1.082	1.032	1.132	2.951	2.807	3.101	1.273	1.223	1.322	3.570	3.396	3.753
5 in 100 False Negative	0.000	-	-	1.000	-	-	0.000	-	-	1.000	-	-
**Consultation Method**												
Online Consultation	0.171	0.097	0.245	1.187	1.102	1.278	0.214	0.140	0.288	1.239	1.151	1.334
Pharmacy Consultation	0.100	0.027	0.173	1.106	1.028	1.189	0.194	0.121	0.268	1.215	1.129	1.307
GP Consultation	0.159	0.085	0.232	1.172	1.089	1.262	0.178	0.104	0.251	1.194	1.110	1.285
Sexual Health Clinic Consultation	0.000	-	-	1.000	-	-	0.000	-	-	1.000	-	-
**Access to Healthcare Professional**												
Phone	-0.065	-0.136	0.005	0.937	0.873	1.005	-0.039	-0.110	0.032	0.962	0.896	1.032
Instant Messenger	-0.006	-0.076	0.063	0.994	0.927	1.065	0.060	-0.010	0.130	1.062	0.990	1.139
Email	0.033	-0.036	0.103	1.034	0.965	1.108	0.060	-0.009	0.130	1.062	0.991	1.139
Face to Face	0.000	-	-	1.000	-	-	0.000	-	-	1.000	-	-
**How you get Antibiotics**												
Post to Home	-0.004	-0.078	0.070	0.996	0.925	1.072	0.027	-0.046	0.101	1.028	0.955	1.106
Post to Collection Point	0.079	0.007	0.151	1.082	1.007	1.163	-0.017	-0.089	0.055	0.983	0.915	1.056
Collect from Pharmacy	0.062	-0.014	0.138	1.064	0.986	1.149	0.083	0.007	0.160	1.087	1.007	1.173
Collect from Sexual Health Clinic	0.000	-	-	1.000	-	-	0.000	-	-	1.000	-	-

For peer review only

Table 3.3 - Age Range Subgroup Analysis Coefficients and Odds Ratios, including Respective 95% Confidence Intervals

	**16-18 *n=415***						**19-21 *n=406***						**22-24 *n=409***					
	**Coeffi-cient**	**Lower 95% CI**	**Upper 95% CI**	**Odds Ratio**	**Lower 95% CI**	**Upper 95% CI**	**Coeffi-cient**	**Lower 95% CI**	**Upper 95% CI**	**Odds Ratio**	**Lower 95% CI**	**Upper 95% CI**	**Coeffi-cient**	**Lower 95% CI**	**Upper 95% CI**	**Odds Ratio**	**Lower 95% CI**	**Upper 95% CI**
**How you Test**																		
Self-Test	0.509	0.395	0.623	1.664	1.485	1.865	0.297	0.182	0.412	1.346	1.200	1.510	0.639	0.523	0.755	1.895	1.687	2.128
Post	0.390	0.275	0.505	1.477	1.316	1.657	0.129	0.013	0.245	1.138	1.013	1.277	0.397	0.281	0.513	1.487	1.324	1.670
Pharmacy	0.239	0.137	0.342	1.270	1.147	1.408	0.021	-0.082	0.124	1.021	0.921	1.132	0.170	0.067	0.273	1.185	1.069	1.314
Education/ Work	-0.218	-0.322	-0.114	0.804	0.725	0.892	-0.215	-0.319	-0.112	0.806	0.727	0.894	-0.160	-0.265	-0.056	0.852	0.768	0.945
GP Practice	0.081	-0.025	0.187	1.085	0.975	1.206	0.035	-0.072	0.141	1.035	0.931	1.152	-0.061	-0.168	0.046	0.941	0.846	1.047
Sexual Health Clinic	0.000	-	-	1.000	-	-	0.000	-	-	1.000	-	-	0.000	-	-	1.000	-	-
**Time to Result**																		
30 Mins	0.567	0.474	0.661	1.763	1.606	1.936	0.636	0.542	0.730	1.888	1.719	2.074	0.572	0.478	0.666	1.772	1.613	1.947
2 Hours	0.282	0.183	0.381	1.326	1.201	1.464	0.437	0.338	0.536	1.548	1.402	1.709	0.295	0.196	0.395	1.344	1.216	1.484
7 Days	0.000	-	-	1.000	-	-	0.000	-	-	1.000	-	-	0.000	-	-	1.000	-	-
14 Days	-0.130	-0.217	-0.044	0.878	0.805	0.957	-0.125	-0.213	-0.038	0.882	0.808	0.963	-0.189	-0.277	-0.102	0.828	0.758	0.903
**Accuracy**																		
2 in 100 False Negative	1.196	1.135	1.257	3.307	3.112	3.514	1.151	1.090	1.212	3.161	2.974	3.361	1.188	1.127	1.250	3.282	3.086	3.489
5 in 100 False Negative	0.000	-	-	1.000	-	-	0.000	-	-	1.000	-	-	0.000	-	-	1.000	-	-
**Consultation Method**																		
Online Consultation	0.171	0.081	0.262	1.187	1.085	1.299	0.182	0.092	0.273	1.200	1.096	1.314	0.226	0.135	0.317	1.253	1.144	1.373
Pharmacy Consultation	0.154	0.065	0.244	1.167	1.067	1.276	0.190	0.100	0.279	1.209	1.105	1.322	0.098	0.007	0.188	1.102	1.007	1.206
GP Consultation	0.246	0.157	0.335	1.279	1.170	1.398	0.133	0.043	0.223	1.142	1.044	1.250	0.125	0.035	0.215	1.133	1.036	1.240
Sexual Health Clinic Consultation	0.000	-	-	1.000	-	-	0.000	-	-	1.000	-	-	0.000	-	-	1.000	-	-

For peer review only

	16-18 *n=415*						19-21 *n=406*						22-24 *n=409*					
	**Coeffi-cient**	**Lower 95% CI**	**Upper 95% CI**	**Odds Ratio**	**Lower 95% CI**	**Upper 95% CI**	**Coeffi-cient**	**Lower 95% CI**	**Upper 95% CI**	**Odds Ratio**	**Lower 95% CI**	**Upper 95% CI**	**Coeffi-cient**	**Lower 95% CI**	**Upper 95% CI**	**Odds Ratio**	**Lower 95% CI**	**Upper 95% CI**
**Access to Healthcare Professional**																		
Phone	0.001	-0.085	0.087	1.001	0.918	1.091	-0.104	-0.190	-0.017	0.902	0.827	0.983	-0.055	-0.142	0.031	0.946	0.868	1.032
Instant Messenger	0.102	0.017	0.188	1.108	1.017	1.207	-0.024	-0.109	0.062	0.977	0.897	1.064	0.001	-0.085	0.087	1.001	0.918	1.091
Email	0.075	-0.009	0.160	1.078	0.991	1.174	0.037	-0.048	0.122	1.038	0.954	1.130	0.029	-0.056	0.115	1.030	0.945	1.122
Face to Face	0.000	-	-	1.000	-	-	0.000	-	-	1.000	-	-	0.000	-	-	1.000	-	-
**How you get Antibiotics**																		
Post to Home	-0.024	-0.114	0.066	0.977	0.893	1.068	0.010	-0.081	0.100	1.010	0.922	1.106	0.047	-0.043	0.137	1.048	0.957	1.147
Post to Collection Point	0.010	-0.078	0.097	1.010	0.925	1.102	0.035	-0.053	0.123	1.036	0.948	1.131	0.046	-0.042	0.135	1.047	0.959	1.144
Collect from Pharmacy	0.039	-0.054	0.132	1.039	0.947	1.141	0.101	0.008	0.195	1.106	1.008	1.215	0.077	-0.017	0.171	1.080	0.983	1.186
Collect from Sexual Health Clinic	0.000	-	-	1.000	-	-	0.000	-	-	1.000	-	-	0.000	-	-	1.000	-	-

For peer review only

Table 3.4 - Relationship Status Subgroup Analysis Coefficients and Odds Ratios, including Respective 95% Confidence Intervals

	Single *n=615*						Sexual Relationship with One Partner *n=512*					
	**Coefficient**	**Lower 95% CI**	**Upper 95% CI**	**Odds Ratio**	**Lower 95% CI**	**Upper 95% CI**	**Coefficient**	**Lower 95% CI**	**Upper 95% CI**	**Odds Ratio**	**Lower 95% CI**	**Upper 95% CI**
**How you Test**												
Self-Test	0.490	0.396	0.584	1.632	1.486	1.792	0.538	0.434	0.641	1.712	1.543	1.899
Post	0.322	0.227	0.416	1.380	1.255	1.516	0.322	0.218	0.425	1.380	1.244	1.530
Pharmacy	0.199	0.115	0.283	1.220	1.122	1.327	0.104	0.011	0.196	1.109	1.011	1.217
Education/ Work	-0.162	-0.247	-0.077	0.851	0.781	0.926	-0.251	-0.344	-0.157	0.778	0.709	0.854
GP Practice	-0.006	-0.093	0.081	0.994	0.911	1.085	0.063	-0.032	0.159	1.066	0.969	1.172
Sexual Health Clinic	0.000	-	-	1.000	-	-	0.000	-	-	1.000	-	-
**Time to Result**												
30 Mins	0.548	0.471	0.625	1.730	1.602	1.867	0.640	0.556	0.724	1.896	1.743	2.062
2 Hours	0.271	0.190	0.352	1.311	1.209	1.422	0.416	0.327	0.505	1.516	1.387	1.657
7 Days	0.000	-	-	1.000	-	-	0.000	-	-	1.000	-	-
14 Days	-0.148	-0.220	-0.077	0.862	0.803	0.926	-0.149	-0.227	-0.071	0.862	0.797	0.932
**Accuracy**												
2 in 100 False Negative	1.186	1.137	1.236	3.275	3.116	3.443	1.196	1.141	1.250	3.305	3.129	3.492
5 in 100 False Negative	0.000	-	-	1.000	-	-	0.000	-	-	1.000	-	-
**Consultation Method**												
Online Consultation	0.145	0.071	0.219	1.156	1.074	1.245	0.251	0.169	0.332	1.285	1.185	1.394
Pharmacy Consultation	0.108	0.035	0.182	1.115	1.036	1.199	0.203	0.123	0.284	1.225	1.130	1.328
GP Consultation	0.150	0.077	0.223	1.162	1.080	1.250	0.173	0.092	0.253	1.189	1.097	1.288
Sexual Health Clinic Consultation	0.000	-	-	1.000	-	-	0.000	-	-	1.000	-	-
**Access to Healthcare Professional**												
Phone	-0.059	-0.130	0.011	0.942	0.878	1.011	-0.077	-0.155	0.000	0.926	0.856	1.000
Instant Messenger	0.025	-0.045	0.095	1.025	0.956	1.099	-0.009	-0.086	0.068	0.991	0.918	1.071
Email	0.066	-0.003	0.136	1.069	0.997	1.145	0.001	-0.076	0.077	1.001	0.927	1.080
Face to Face	0.000	-	-	1.000	-	-	0.000	-	-	1.000	-	-
**How you get Antibiotics**												
Post to Home	-0.044	-0.118	0.030	0.957	0.889	1.030	0.053	-0.028	0.134	1.055	0.973	1.144
Post to Collection Point	-0.032	-0.104	0.040	0.968	0.901	1.041	0.070	-0.009	0.149	1.072	0.991	1.160
Collect from Pharmacy	0.067	-0.009	0.143	1.070	0.991	1.154	0.055	-0.029	0.139	1.056	0.971	1.149
Collect from Sexual Health Clinic	0.000	-	-	1.000	-	-	0.000	-	-	1.000	-	-

For peer review only

Table 3.5 - Previous STI Testing Subgroup Analysis Coefficients and Odds Ratios, including Respective 95% Confidence Interval

	Previous Test n=393						No Previous Test *n=790*					
	**Coefficient**	**Lower 95% CI**	**Upper 95% CI**	**Odds Ratio**	**Lower 95% CI**	**Upper 95% CI**	**Coefficient**	**Lower 95% CI**	**Upper 95% CI**	**Odds Ratio**	**Lower 95% CI**	**Upper 95% CI**
**How you Test**												
Self-Test	0.422	0.305	0.539	1.524	1.356	1.714	0.518	0.434	0.601	1.678	1.544	1.824
Post	0.164	0.047	0.280	1.178	1.048	1.323	0.382	0.298	0.466	1.465	1.347	1.594
Pharmacy	-0.056	-0.160	0.048	0.945	0.852	1.049	0.245	0.170	0.320	1.277	1.185	1.377
Education/ Work	-0.273	-0.377	-0.168	0.761	0.686	0.845	-0.150	-0.225	-0.074	0.861	0.798	0.928
GP Practice	-0.122	-0.229	-0.014	0.885	0.795	0.986	0.097	0.019	0.174	1.101	1.019	1.190
Sexual Health Clinic	0.000	-	-	1.000	-	-	0.000	-	-	1.000	-	-
**Time to Result**												
30 Mins	0.592	0.498	0.687	1.808	1.645	1.988	0.586	0.518	0.654	1.797	1.679	1.923
2 Hours	0.305	0.205	0.406	1.357	1.227	1.500	0.353	0.281	0.425	1.423	1.325	1.529
7 Days	0.000	-	-	1.000	-	-	0.000	-	-	1.000	-	-
14 Days	-0.125	-0.213	-0.037	0.882	0.808	0.963	-0.156	-0.219	-0.093	0.856	0.803	0.912
**Accuracy**												
2 in 100 False Negative	1.099	1.037	1.160	3.000	2.820	3.191	1.248	1.203	1.292	3.482	3.331	3.640
5 in 100 False Negative	0.000	-	-	1.000	-	-	0.000	-	-	1.000	-	-
**Consultation Method**												
Online Consultation	0.161	0.069	0.252	1.174	1.071	1.287	0.205	0.140	0.271	1.228	1.150	1.311
Pharmacy Consultation	0.094	0.003	0.184	1.098	1.003	1.203	0.168	0.103	0.233	1.183	1.108	1.262
GP Consultation	0.106	0.015	0.197	1.112	1.015	1.217	0.195	0.130	0.260	1.215	1.139	1.297
Sexual Health Clinic Consultation	0.000	-	-	1.000	-	-	0.000	-	-	1.000	-	-
**Access to Healthcare Professional**												
Phone	-0.054	-0.142	0.034	0.947	0.868	1.034	-0.048	-0.110	0.015	0.953	0.895	1.015
Instant Messenger	0.044	-0.042	0.131	1.045	0.958	1.140	0.025	-0.037	0.087	1.025	0.963	1.091
Email	0.048	-0.038	0.134	1.049	0.962	1.144	0.053	-0.009	0.114	1.054	0.991	1.121
Face to Face	0.000	-	-	1.000	-	-	0.000	-	-	1.000	-	-
**How you get Antibiotics**												
Post to Home	0.011	-0.081	0.102	1.011	0.922	1.107	0.020	-0.046	0.085	1.020	0.955	1.089
Post to Collection Point	0.034	-0.056	0.123	1.034	0.946	1.131	0.034	-0.029	0.098	1.035	0.971	1.103
Collect from Pharmacy	0.083	-0.012	0.177	1.086	0.988	1.194	0.075	0.008	0.143	1.078	1.008	1.153
Collect from Sexual Health Clinic	0.000	-	-	1.000	-	-	0.000	-	-	1.000	-	-

For peer review only

STROBE Statement—checklist of items that should be included in reports of observational studies

	Item No	Recommendation
**Title and abstract**	1	(*a*) Indicate the study's design with a commonly used term in the title or the abstract ✔ (page 1)
		(*b*) Provide in the abstract an informative and balanced summary of what was done and what was found ✔ (page 2)
**Introduction**		
Background/rationale	2	Explain the scientific background and rationale for the investigation being reported ✔ (page 4)
Objectives	3	State specific objectives, including any prespecified hypotheses ✔ (page 5)
**Methods**		
Study design	4	Present key elements of study design early in the paper ✔ (page 5-6)
Setting	5	Describe the setting, locations, and relevant dates, including periods of recruitment, exposure, follow-up, and data collection ✔ (page 7)
Participants	6	(*a*) *Cohort study*—Give the eligibility criteria, and the sources and methods of selection of participants. Describe methods of follow-up ✔ (page 7)<br>*Case-control study*—Give the eligibility criteria, and the sources and methods of case ascertainment and control selection. Give the rationale for the choice of cases and controls<br>*Cross-sectional study*—Give the eligibility criteria, and the sources and methods of selection of participants
		(*b*) *Cohort study*—For matched studies, give matching criteria and number of exposed and unexposed<br>*Case-control study*—For matched studies, give matching criteria and the number of controls per case
Variables	7	Clearly define all outcomes, exposures, predictors, potential confounders, and effect modifiers. Give diagnostic criteria, if applicable ✔ (page 8)
Data sources/ measurement	8*	For each variable of interest, give sources of data and details of methods of assessment (measurement). Describe comparability of assessment methods if there is more than one group ✔ (page 8)
Bias	9	Describe any efforts to address potential sources of bias ✔ (page 8)
Study size	10	Explain how the study size was arrived at ✔ (page 7)
Quantitative variables	11	Explain how quantitative variables were handled in the analyses. If applicable, describe which groupings were chosen and why ✔ (page 7)
Statistical methods	12	(*a*) Describe all statistical methods, including those used to control for confounding ✔ (page 7-8)
		(*b*) Describe any methods used to examine subgroups and interactions ✔ (page 8)
		(*c*) Explain how missing data were addressed
		(*d*) *Cohort study*—If applicable, explain how loss to follow-up was addressed<br>*Case-control study*—If applicable, explain how matching of cases and controls was addressed<br>*Cross-sectional study*—If applicable, describe analytical methods taking account of sampling strategy
		(*e*) Describe any sensitivity analyses
**Results**		
Participants	13*	(a) Report numbers of individuals at each stage of study—eg numbers potentially eligible,

For peer review only - http://bmjopen.bmj.com/site/about/guidelines.xhtml

		examined for eligibility, confirmed eligible, included in the study, completing follow-up, and analysed ✔ (page 8)
		(b) Give reasons for non-participation at each stage
		(c) Consider use of a flow diagram
Descriptive data	14*	(a) Give characteristics of study participants (eg demographic, clinical, social) and information on exposures and potential confounders ✔ (page 8 & Supplementary Information File 2)
		(b) Indicate number of participants with missing data for each variable of interest
		(c) *Cohort study*—Summarise follow-up time (eg, average and total amount)
Outcome data	15*	*Cohort study*—Report numbers of outcome events or summary measures over time ✔ (page 8-9)
		Case-control study—Report numbers in each exposure category, or summary measures of exposure
		Cross-sectional study—Report numbers of outcome events or summary measures
Main results	16	(*a*) Give unadjusted estimates and, if applicable, confounder-adjusted estimates and their precision (eg, 95% confidence interval). Make clear which confounders were adjusted for and why they were included ✔ (page 8-9)
		(*b*) Report category boundaries when continuous variables were categorized
		(*c*) If relevant, consider translating estimates of relative risk into absolute risk for a meaningful time period
Other analyses	17	Report other analyses done—eg analyses of subgroups and interactions, and sensitivity analyses ✔ (page 9)
**Discussion**		
Key results	18	Summarise key results with reference to study objectives ✔ (page 9-10)
Limitations	19	Discuss limitations of the study, taking into account sources of potential bias or imprecision. Discuss both direction and magnitude of any potential bias ✔ (page 10)
Interpretation	20	Give a cautious overall interpretation of results considering objectives, limitations, multiplicity of analyses, results from similar studies, and other relevant evidence ✔ (page 11)
Generalisability	21	Discuss the generalisability (external validity) of the study results ✔ (page 11)
**Other information**		
Funding	22	Give the source of funding and the role of the funders for the present study and, if applicable, for the original study on which the present article is based ✔ (page 12)

*Give information separately for cases and controls in case-control studies and, if applicable, for exposed and unexposed groups in cohort and cross-sectional studies.

For peer review only

For peer review only - http://bmjopen.bmj.com/site/about/guidelines.xhtml
